# “Activated Borane”: A Porous Borane Cluster Polymer as an Efficient Lewis Acid-Based Catalyst

**DOI:** 10.1021/acscatal.3c04011

**Published:** 2023-10-30

**Authors:** Martin Lamač, Béla Urbán, Michal Horáček, Daniel Bůžek, Lucie Leonová, Aleš Stýskalík, Anna Vykydalová, Karel Škoch, Matouš Kloda, Andrii Mahun, Libor Kobera, Kamil Lang, Michael G. S. Londesborough, Jan Demel

**Affiliations:** † Department of Molecular Electrochemistry and Catalysis, J. Heyrovsky Institute of Physical Chemistry of the Czech Academy of Sciences Dolejškova 2155, 182 00 Prague 8, Czech Republic; ‡ Department of Materials Chemistry, Institute of Inorganic Chemistry of the Czech Academy of Sciences, Husinec-Řež 1001, 250 68 Řež, Czech Republic; § Department of Chemistry, Masaryk University, Kotlářská 2, 611 37 Brno, Czech Republic; ∥ Department of Structural Analysis, Institute of Macromolecular Chemistry of the Czech Academy of Sciences, Heyrovského nám. 2, 162 06 Prague 6, Czech Republic

**Keywords:** activated borane, Lewis acid catalyst, heterogeneous catalysis, deoxygenation reaction, ethanol dehydration, Gutmann−Beckett method, solid-state NMR

## Abstract

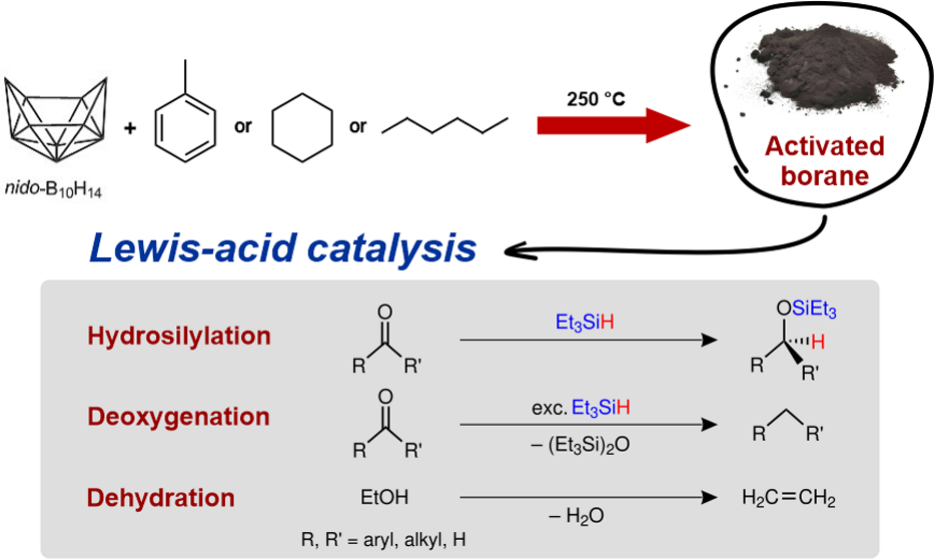

Borane cluster-based porous covalent networks, named activated borane (**ActB**), were prepared by cothermolysis of decaborane(14) (*nido*-B_10_H_14_) and selected hydrocarbons (toluene, **ActB-Tol;** cyclohexane, **ActB-*****cy*****Hx**; and *n*-hexane, **ActB-*****n*****Hx**) under anaerobic conditions. These amorphous solid powders exhibit different textural and Lewis acid (LA) properties that vary depending on the nature of the constituent organic linker. For **ActB-Tol**, its LA strength even approaches that of the commonly used molecular LA, B(C_6_F_5_)_3_. Most notably, **ActB**s can act as heterogeneous LA catalysts in hydrosilylation/deoxygenation reactions with various carbonyl substrates as well as in the gas-phase dehydration of ethanol. These studies reveal the potential of **ActB**s in catalytic applications, showing (a) the possibility for tuning catalytic reaction outcomes (selectivity) in hydrosilylation/deoxygenation reactions by changing the material’s composition and (b) the very high activity toward ethanol dehydration that exceeds the commonly used γ-Al_2_O_3_ by achieving a stable conversion of ∼93% with a selectivity for ethylene production of ∼78% during a 17 h continuous period on stream at 240 °C.

## Introduction

Lewis acid-catalyzed reactions are numerous and of considerable significance.^1^ In chemical and petrochemical industries, it is well established that heterogeneous catalysts offer an important advantage over their homogeneous counterparts that they allow the design of continuous production processes. For this and other advantageous reasons, significant resources have, in recent decades, been allocated to the development of novel acid-based heterogeneous catalysts.

Traditionally, heterogeneous catalysts such as inorganic oxides, aluminosilicates, sulfonated zirconia, zeolites, and alumina halides have all contained metals as the Lewis acid centers. Alternatively, the incorporation of boron into zeolites and amorphous silica has been thoroughly studied and found to promote the oxidative dehydrogenation of alkanes to alkenes (ODH).^2^ Interestingly, however, isolated tricoordinated BO_3_ sites in MCM-22 have been shown to be inactive.^3^ Instead, an amorphous oligomeric boron oxide/hydroxide layer was shown to be active in ODH, the activity and selectivity being beneficially influenced by the ability of the catalyst to form radicals.^4^ Mixed boria–alumina oxides represent another family of boron-containing inorganic catalysts. These materials can be described as mildly acidic solids and are used in alcohol dehydration,^5^ Beckmann rearrangement,^6^ and phenyl glyoxal conversion.^7^

The traditional inorganic catalysts described above are very robust and effective even at very high temperatures. However, the set of reactions catalyzed by these materials is different from those catalyzed by molecular tricoordinated boron catalysts, which can, e.g., utilize “frustrated Lewis pair” (FLP) reactivity.^8^ The first examples of such tricoordinated boron molecular catalysts were based on boron halides BX_3_ and perfluoroalkyl boranes; however, such compounds are difficult to handle and/or are thermolabile. Since the 1980s, B(C_6_F_5_)_3_ (commonly referred to as “BCF”) has become the homogeneous catalyst of choice for many applications because of the strong electron-withdrawing effect, bulkiness of the pentafluorophenyl substituents, and the general ease and convenience of its use.^9^ Indeed, the versatility of BCF provided the impetus to its heterogenization by linking it to nonreactive solid supports. Thus, silica,^10^ alumina,^11^ graphene oxide,^12^ organic polymers,^13^ and other substrates were modified with -B(C_6_F_5_)_2_ acid centers. However, these heterogenized catalysts are laborious to synthesize, and their catalytic activity is in most cases significantly lower than that of the homogeneous BCF counterpart.

Recently, borane clusters (usually represented by derivatives of icosahedral carborane C_2_B_10_H_12_) have been proposed as a viable alternative to the commonly used polyfluorinated aryl substituents at the tricoordinated boron atom sites of BCF.^14^ Kondo and co-workers have recently reported the catalytic dehydration of ethanol to ethylene by hydrogenated borophene, a 2D material with an empirical formula H_1_B_1_.^15^ These HB sheets likely possess Brønsted sites represented by protons on the edges of the 2D structure, but the precise nature of the catalytically active sites in these materials is still unknown. This development prompted us to cast a wider net in the borane cluster field and test the boron acid centers found in ***activated borane*** (abbreviated hereafter as **ActB**)^16^ for heterogeneous catalytic activity. **ActB** is a porous polymer that is formed by cothermolysis of decaborane(14) (*nido*-B_10_H_14_) and organic solvents such as toluene or hexane. We have shown that the structure of **ActB** is most probably composed of *closo* borane clusters linked together by organic linking moieties.^17^

Here, in this paper, we present an improved strategy for the synthesis of **ActB** and present evidence for the catalytic activity of its Lewis acidic boron sites as well as a comparison of its performance compared with contemporary catalysts. The nature of the Lewis acid sites in **ActB** was characterized by the Gutmann–Beckett method (^31^P solid-state NMR after the adsorption of Et_3_PO) and by temperature-programmed desorption of ammonia. Furthermore, we showcase the utility of **ActB** for hydrosilylation/deoxygenation reactions of various carbonyl compounds and also in the dehydration of ethanol into ethylene. In the latter reaction, **ActB** exerted a higher catalytic activity than commonly used alumina and excellent catalyst stability even after 17 h on stream.

## Results and Discussion

### Synthesis of Activated Borane

**Scheme 1:**
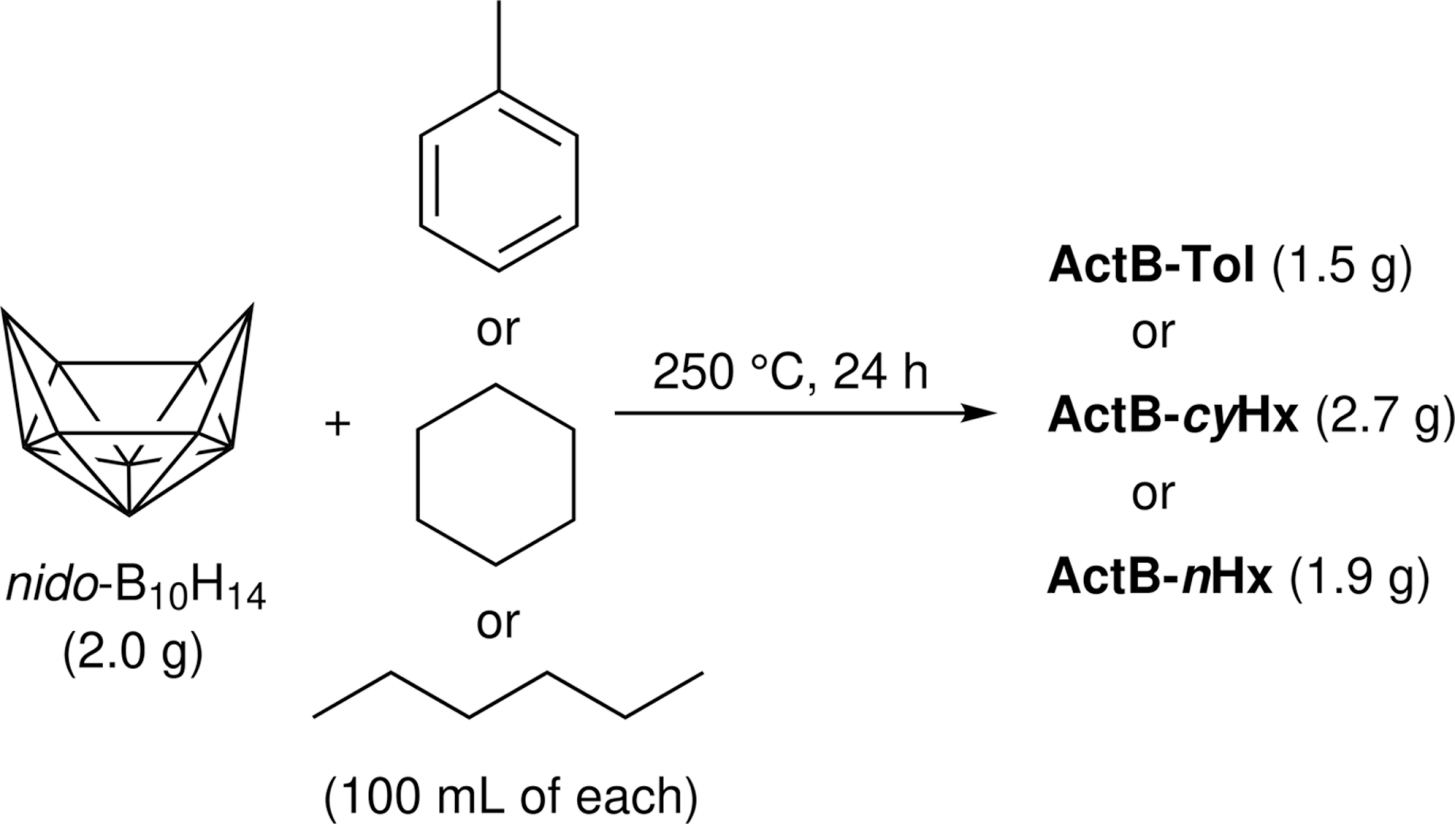
Synthesis of **ActB** Materials

Building on our previous findings,^16^ we have improved the synthesis of **ActB** and, by using an Ar-filled glovebox, a stainless steel autoclave (Berghof BR-300), and a Schlenk line, we have prepared pristine samples of **ActB** devoid of B–OH groups (see below IR and ssNMR). In order to compare the effect of aromatic, cyclic aliphatic, and acyclic aliphatic linking molecules, we have prepared **ActB**s by heating *nido*-B_10_H_14_ in toluene, cyclohexane, and *n*-hexane to 250 °C in a high-pressure reactor for 24 h (Scheme 1). The crude dark solids were Soxhlet-extracted with the same solvent as that used for the synthesis to remove all unreacted and partially unreacted impurities. The residual solvent was removed by heating to 100 °C under vacuum for 4 h. For details on synthesis, see the SI. The samples are denoted as **ActB-Tol** (toluene), **ActB-cyHx** (cyclohexane), and **ActB-*****n*****Hx** (*n*-hexane).

### Characterization

**Figure 1:**
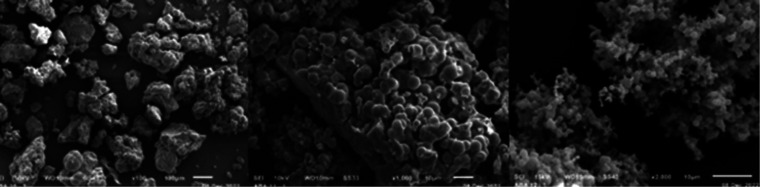
SEM images of **ActB-Tol** (left), **ActB-cyHx** (middle), and **ActB-nHx** (right). For additional images, see the SI.

All prepared **ActB**s are solids lacking long-range order. **ActB-Tol** and **ActB-*****cy*****Hx** are black powders, whereas **ActB-*****n*****Hx** is brown-colored. This crude observation is consistent with the materials’ UV–vis spectra, in which all **ActB**s absorb strongly below 1000 nm, with the absorption edge for **ActB-*****n*****Hx** being shifted to lower wavelengths in comparison with **ActB-Tol** and **ActB-*****cy*****Hx**. This translates to optical band gaps of 1.19, 1.20, and 1.34 eV for **ActB-Tol**, **ActB-*****cy*****Hx**, and **ActB-*****n*****Hx**, respectively, as derived from Tauc plots using the Kubelka–Munk-transformed reflectance spectra (for details, see the SI). The elemental analysis of **ActBs** was done by the standard CHN analysis, and the content of boron was determined by ICP-MS (see the SI for details). ICP-MS was also used to check for the presence of metal traces; from the comparison with blank samples, we did not observe elevated amounts of metals, which could be responsible for catalytic activity (such as Pd, Ni, Cu, Cr, Fe, Zn, Cr, etc.). Scanning electron microscopy (SEM) (Figure 1) shows larger particles of irregular shape for **ActB-Tol** and **ActB-*****cy*****Hx**, while **ActB-*****n*****Hx** forms spherical particles approximately 1 μm in diameter.

**Figure 2:**
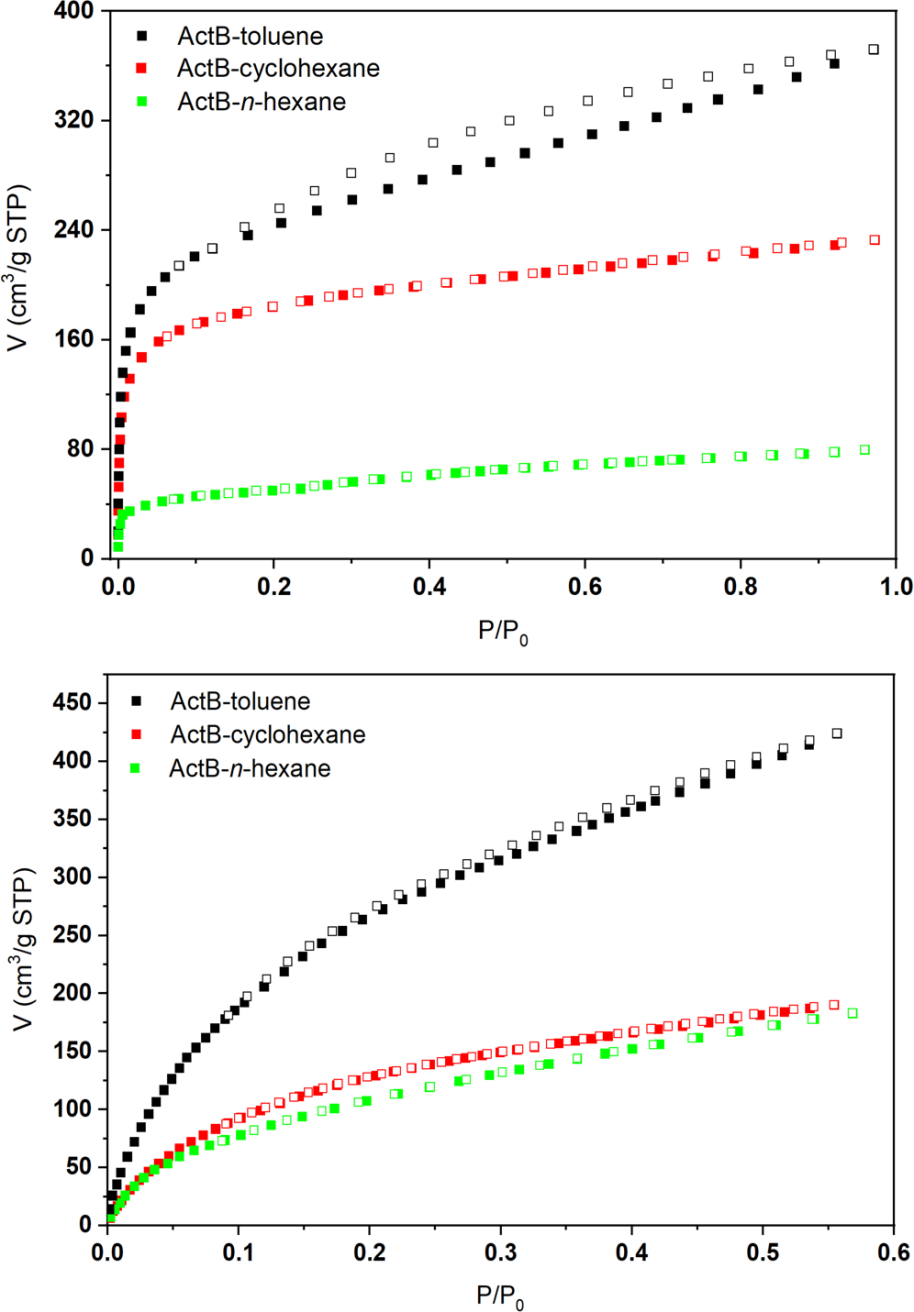
Adsorption isotherms of Ar at 87 K (top) and CO_2_ at 195 K (bottom) for **ActB-Tol**, **ActB-*cy*Hx**, and **ActB-*n*Hx**.

The Ar adsorption isotherms for all **ActB**s correspond well to those typical for microporous materials (Figure 2). **ActB-Tol** and **ActB-*****cy*****Hx** contain pore size maxima between 0.9 and 1.6 nm, while **ActB-*****n*****Hx** contains pore size maxima between 1.0 and 2.0 nm (Figure S4). The extent of porosity and specific surface area, however, varies greatly between the derivatives, with the clear trend **ActB-Tol** > **ActB-*****cy*****Hx** > **ActB-*****n*****Hx** (see Table 1). The adsorption of CO_2_ follows a similar pattern, with the highest adsorption achieved for **ActB-Tol** and a lower adsorption achieved for **ActB-*****cy*****Hx** and **ActB-*****n*****Hx**. In this case, however, no significant difference between the latter two was observed. To confirm the batch-to-batch reproducibility of the synthesis of **ActB-Tol**, we compared the adsorption isotherms of Ar and pore size distribution for individual batches (see Figures S5 and S6 in the SI).

**Table 1:** Specific Surface Areas, Pore Diameters, and Pore Volumes for **ActB-Tol**, **ActB-cyHx**, and **ActB-nHx**

sample	*S*_BET_ (m^2^g^–1^)^a^	pore size (nm)^b^	*V*_pore_ (cm^3^g^–1^)^c^
**ActB-Tol**	784	0.9–1.6	0.47
**ActB-*****cy*****Hx**	608	0.9–1.5	0.30
**ActB-*****n*****Hx**	160	1.0–2.0	0.10

a BET specific surface area.

b Pore size maxima calculated by the MDFT method.

c Total pore volume at p/p0 = 0.99.

**Figure 3:**
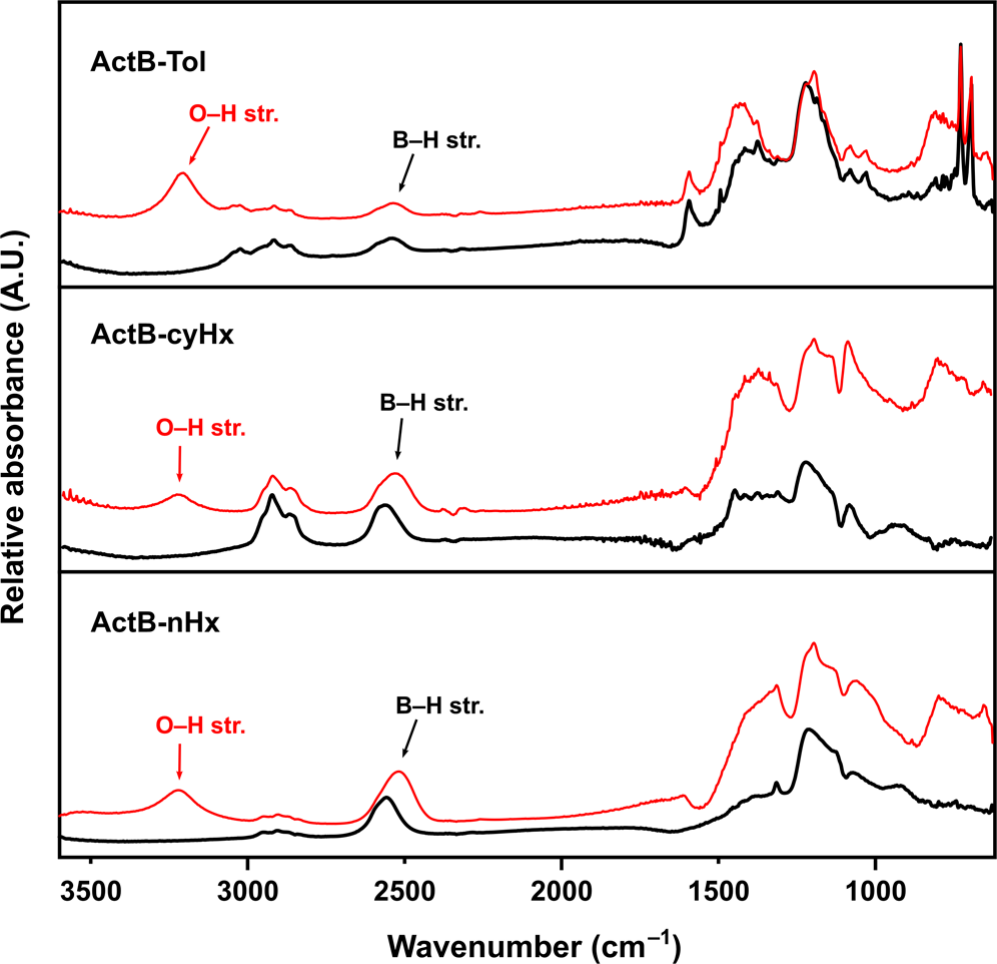
FTIR (ATR-Si) spectra of **ActB-Tol** (top), **ActB-cyHx** (middle), and **ActB-nHx** (bottom); red lines correspond to samples exposed to air for 1 min.

The FTIR spectra (Figure 3) for these species contain peaks corresponding to both constituent moieties—borane cluster and organic linker. Most notably, the peaks at approximately 2900 and 2550 cm^–1^ correspond to C–H and B–H stretchings, respectively. In all cases, the absence of any peak at around 1900 cm^–1^ suggests the absence of μ-H (B–H–B bridges) in the structure. As expected, the peak at approximately 1600 cm^–1^, which can be attributed to the aromatic stretching vibration, is present only in **ActB-Tol**. A significant difference from the original **ActB** spectrum published earlier^16^ is the absence of the BO–H stretching peak over 3000 cm^–1^, which can be explained by the different preparations and measurement procedures used that avoid oxygen contamination. To study the effect of humid air, we exposed **ActB**s for 1 min to air and remeasured FTIR, which in all cases revealed a quick water adsorption onto the material, manifested by the appearance of the O–H stretching band at around 3200 cm^–1^.

The thermal stabilities of **ActB**s were studied by TGA coupled with MS (Figures S10–S19). **ActB-Tol** showed the lowest stability with the onset of its main degradation step (*T*_onset_) at around 245 °C, compared to the higher limits measured for **ActB-*****cy*****Hx** and **ActB-*****n*****Hx** at 319 and 322 °C, respectively. For a detailed discussion, see the SI.

**Figure 4:**
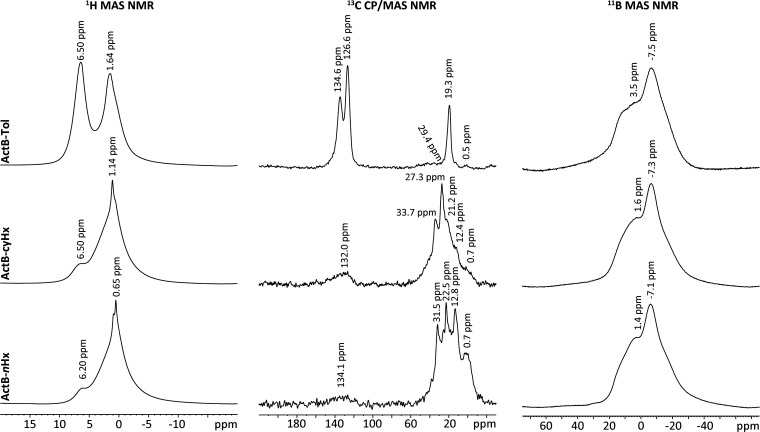
^1^H MAS NMR (left column), ^13^C CP/MAS NMR (middle column), and ^11^B MAS NMR (right column) spectra of **ActB-Tol**, **ActB-cyHx**, and **ActB-nHx** samples.

The ^1^H MAS NMR spectrum of **ActB-Tol** (Figure 4, left column) reveals two broad signals at 1.64 and 6.5 ppm, which can be attributed to the methyl group and aromatic protons of toluene, respectively, overlapped with signals from borane clusters.^16^ The ^1^H MAS NMR spectra of **ActB-*****cy*****Hx** and **ActB-*****n*****Hx** samples show a broad and asymmetric dominant peak at 1.14 and 0.65 ppm, respectively, assigned to aliphatic protons overlapped with signals from borane clusters. Interestingly, both spectra contain a broad shoulder at about 6.5 and 6.2 ppm, respectively, which indicate the presence of unsaturated (−CH=) species, suggesting that partial dehydrogenation of cyclohexane and *n*-hexane occurs during the synthesis (further confirmed by ^13^C CP/MAS NMR; see below). In all cases, signals from open-face “bridging” μ-hydrogens of *nido*-B_10_H_14_ are not detected (the expected range of signals between −4 and 0 ppm),^18^ confirming the transformation of open *nido*-borane clusters into closed cluster derivatives and/or full substitution of the μ-hydrogens^16^ indicated by FTIR. The ^13^C CP/MAS NMR spectrum of **ActB-Tol** (Figure 4, middle column) exhibits three major peaks at 19.3, 126.6, and 134.6 ppm and minor peaks at 0.5 and 29.4 ppm. The major peaks are assigned, respectively, to the methyl carbon and the nonequivalent groups of aromatic carbons from immobilized toluene. The minor peak at 0.5 ppm was attributed to carbon atoms bonded to the borane cluster forming nodal points, similar to the bonding system that was observed for boron carbide systems.^19^ The second minor broad peak at 29.4 ppm, ranging from about −10 to 70 ppm, can be assigned to methyl groups directly connected to borane clusters and/or carbons incorporated into these clusters possibly forming a substituted carborane cluster.^20^ The ^13^C CP/MAS NMR spectra of **ActB-*****cy*****Hx** and **ActB-*****n*****Hx** display a series of partially overlapping signals ranging from about 0 to 40 ppm that correspond well with aliphatic carbons (Figure 4, middle column). The presence of signals around 133 ppm confirms the formation of unsaturated (−CH=) species. Furthermore, the distribution of chemical shifts points to a wide range of chemical environments typical of solids lacking a long-range order. In addition, the signal at around 0.7 ppm on the respective spectra of **ActB-*****cy*****Hx** and **ActB-*****n*****Hx** indicates carbon atoms forming nodal points similarly to what was observed for **ActB-Tol**.

All ^11^B MAS NMR spectra (Figure 4, right column) possess two broad composite signals with maxima at around −7.3 and 1.5 ppm (3.5 ppm in the case of **ActB-Tol**) corresponding to the borane cluster molecules substituted at different positions forming a variety of chemical environments.^16^ Even 2D ^11^B 3Q/MAS NMR spectra did not reveal additional signals and clearly confirmed the structure lacking a long-range order (Figure S8).

### Lewis Acid Sites

The Gutmann–Beckett method is commonly used to assess the relative acidity of molecular Lewis acid-based catalysts. It uses the sensitivity of the ^31^P NMR chemical shift (δ_P_) in Et_3_PO (TEPO) to interactions with Lewis acids.^21^ As a result, the δ_P_ of TEPO can be shifted from 48 to over 90 ppm depending on the strength of the acid–base interaction. In recent years, the Gutmann–Beckett method was also used for porous solid acids using ssNMR. However, in this case, the NMR lines are broadened not only because of the measurement in solid state but also because of the varying chemical environments within the pores.^22^

**Figure 5:**
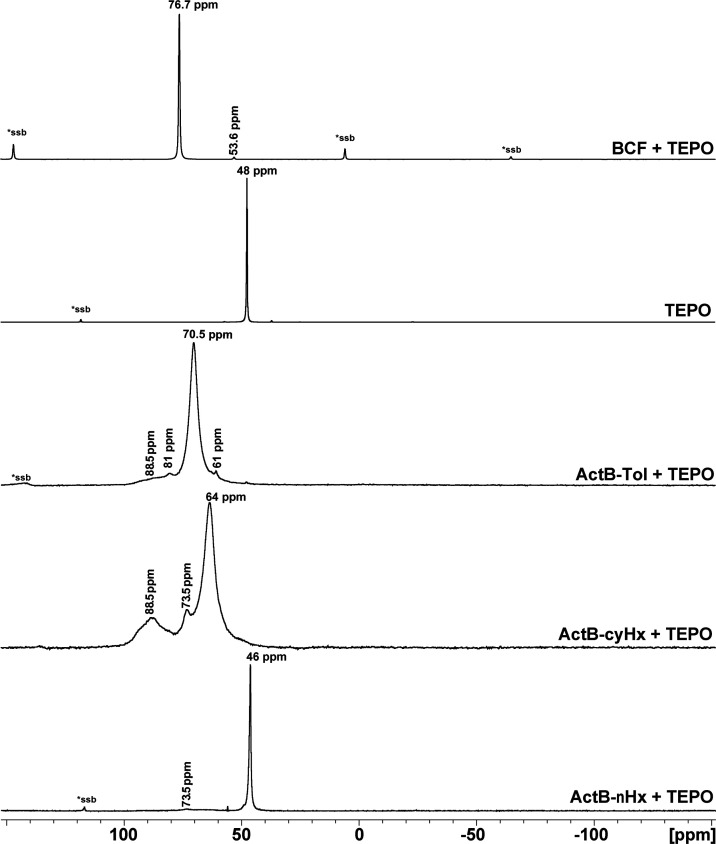
^31^P MAS ssNMR spectra from top: BCF+TEPO adduct, TEPO, **ActB-Tol**+TEPO, **ActB-cyHx**+TEPO, and **ActB-nHx**+TEPO.

From a comparison of all ^31^P MAS NMR spectra (Figure 5) of the investigated samples, it is evident that TEPO interacts only with **ActB-Tol** and **ActB-*****cy*****Hx** and forms LA-TEPO adducts. Interestingly, the signals are very narrow (half-width of dominant signals is ca. 1.3 ± 0.2 kHz), displaying similar sharpness as zeolite-Y-TEPO adduct and much higher sharpness than γ-alumina or micromesoporous zirconosilicates.^22^ Therefore, the LA strength can be compared: BCF > **ActB-Tol** > **ActB-*****cy*****Hx**. Given that **ActB**s do not contain any perfluorinated electron-withdrawing substituents, it points to a remarkable Lewis acidity of **ActB-Tol.** On the other hand, only one sharp signal, with almost unchanged position in comparison with pristine TEPO, was detected in the ^31^P MAS NMR spectrum of **ActB-*****n*****Hx**. This indicates the presence of TEPO molecules in the investigated system but without any detectable chemical interaction.

**Figure 6:**
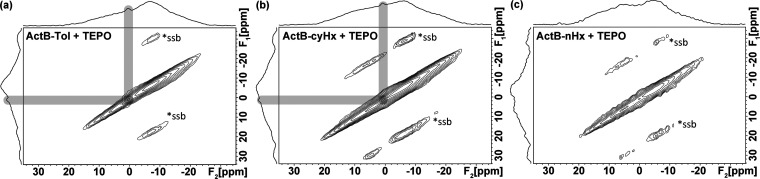
^11^B 3Q/MAS NMR spectra of **ActB-Tol**+TEPO (a), **ActB-cyHx**+TEPO (b), and **ActB-nHx**+TEPO (c). The newly detected signal is highlighted by gray boxes.

^11^B 3Q/MAS NMR spectra after the adsorption of TEPO (Figure 6) display one specific, relatively sharp signal with ^11^B δ_iso_ = 1 ppm (, where F_1_ = 1.3 ± 0.5 ppm and F_2_ = 0.5 ± 0.5 ppm) in **ActB-Tol**+TEPO and **ActB-*****cy*****Hx**+TEPO systems. The shape of the signal and ^11^B NMR shift suggest an almost ideal tetracoordinated geometry of affected boron atoms.^23^ This clearly confirms the Lewis acidity of **ActB-Tol** and **ActB-*****cy*****Hx** by the formation of tetracoordinated borons upon reaction with the Lewis base (TEPO). It should be noted that only a small fraction of borons in **ActBs** are LA sites, and therefore, the peaks are relatively small in comparison with the rest of the spectra. In ^13^C CP/MAS NMR spectra, **ActB**-TEPO samples contain additional signals corresponding to TEPO and toluene, which come from the TEPO treatment; otherwise, the spectra are unchanged in comparison with as-prepared **ActB**s, thus confirming the preserved disordered structure (see Figure S9).

**Figure 7:**
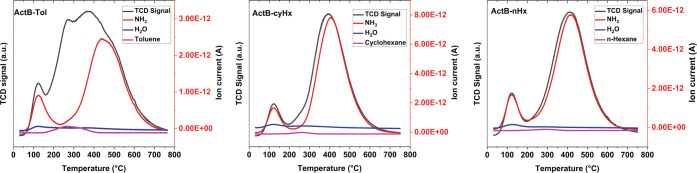
TPD curves for **ActB-Tol** (left), **ActB-cyHx** (middle), and **ActB-nHx** (right).

To quantify the number of acid sites, we employed temperature-programmed desorption of ammonia (TPD). All three samples provided a similar pattern in which ammonia desorbs first at ∼120 °C (weakly adsorbed NH_3_) and then at ∼420 °C (strongly chemisorbed NH_3_; Figure 7 and Table 2). The fraction of the weakly adsorbed NH_3_ out of the total number of NH_3_ molecules adsorbed is very uniform for all three samples and ranges from 7 to 8%. Of note are the high ammonia desorption temperatures (varying from 403 °C for **ActB-*****cy*****Hx** to 438 °C for **ActB-Tol**) that lie within the range of the strong Brønsted acid sites present in HZSM-5 zeolite (400–470 °C)^24^ and much higher than for the acid sites in amorphous aluminosilicates (250–300 °C).^25^ Importantly, **ActB-Tol** possesses the strongest acid sites according to ammonia TPD, which is in line with ^31^P MAS NMR after TEPO adsorption; see above.

**Table 2:** Summary of Ammonia TPD^a^

sample	weakly adsorbed NH_3_ (mmol g^–1^)	chemisorbed NH_3_ (mmol g^–1^)	*T*_des_ of chemisorbed NH_3_ (°C)
**ActB-Tol**	0.30	3.47	438
**ActB-*****cy*****Hx**	0.60	8.10	403
**ActB-*****n*****Hx**	0.66	7.31	415

a The amounts of adsorbed NH_3_ were estimated from the areas under the TPD curves.

The most significant difference between the **ActB**s samples is the total number of NH_3_ molecules adsorbed (Table 2). While **ActB-Tol** exhibited 3.77 mmol g^–1^ adsorbed NH_3_ in total, **ActB-*****n*****Hx** and **ActB-*****cy*****Hx** provided more than twice as much, with the maximum observed for **ActB-*****cy*****Hx** (8.70 mmol g^–1^). These results indicate that **ActB-*****cy*****Hx** and **ActB-*****n*****Hx** contain a significantly higher number of acid sites in comparison to **ActB-Tol**. Interestingly, **ActB-*****n*****Hx** contains a high number of acid sites according to NH_3_ TPD but virtually none according to ^31^P MAS NMR after TEPO adsorption. We assume that these differences originate in the different size and basicity of the probe molecules. Nevertheless, all obtained values are very high, suggesting that there might be multiple NH_3_ molecules adsorbed to each available LA site and/or strongly bound NH_3_ in other areas within the framework not only to the LA centers.

To analyze the ability of **ActB**s to adsorb medium-sized molecules, pyridine and benzene were preadsorbed onto each sample at RT followed by TGA measurement. The difference between the adsorption of neutral molecules (benzene) and basic molecules (pyridine) can be used as a proxy for the estimation of the number of accessible acid sites to medium-sized molecules. Thus, the amount of adsorbed pyridine followed the trend **ActB-Tol** > **ActB-*****cy*****Hx** > **ActB-*****n*****Hx**. Based on these data, we can assume that the number of LA sites accessible to medium-sized molecules is between 1 and 1.5 mmol g^–1^ for **ActB-Tol** and **ActB-*****cy*****Hx** and yet probably only around 0.5 mmol g^–1^ for **ActB-*****n*****Hx** (see Table 3). This correlates well with the specific surface area of the **ActB**s materials as determined by Ar adsorption and the Gutmann–Beckett method utilizing TEPO adsorption but not complementary with the ammonia TPD (for a detailed discussion, see the SI).

**Table 3:** Summary of Quantification of Probe Molecular Adsorption

sample	adsorbed benzene (mmol g^–1^)	adsorbed pyridine (mmol g^–1^)	chemisorbed NH_3_ (mmol g^–1^)	Δ*p*yridine–benzene (mmol g^–1^)^a^
**ActB-Tol**	0.8	2.1	3.47	1.3
**ActB-*****cy*****Hx**	0.3	1.3	8.10	1.0
**ActB-*****n*****Hx**	0.3	0.7	7.31	0.4

a The difference between absorbed benzene and absorbed pyridine.

The origin of the LA boron atoms probably lies in the solvothermal thermolysis of open clusters. It has been observed earlier (e.g., see the work of J. Kennedy^26^) that open borane clusters subject to high energy (e.g., thermolysis, photolysis, and electron bombardment) form clusters with different number of vertices. We made similar observations during the thermal treatment of *nido*-B_10_H_14_ in benzene at 200 °C where we detected substituted B_18_H_22_ clusters.^17^ This change in the number of boron vertices is probably accompanied by the formation of highly reactive lower boranes that are the seeds for LA centers.

### Catalytic Activity

#### Hydrosilylation and Deoxygenation Reactions

Lewis acidic boron molecules, e.g., BCF, are known to activate the Si–H bond.^27^ This can be utilized in hydrosilylation or reductive deoxygenation of carbonyl compounds in mild conditions.^28^ Such reactions cannot be catalyzed by conventional heterogeneous inorganic LA catalysts, e.g., zeolites, which are, on the other hand, known to promote hydrodeoxygenation (HDO) processes during biomass processing.^29^ These reactions, however, typically require high temperatures and pressures of hydrogen gas, as well as the presence of metal dopants, which are responsible for the hydrogenation reactivity. In addition, hydrosilylation reactions, related to those described herein, were only reported using solid-supported metal-based catalysts in several literature reports.^30^ For this reason, we endeavored to test the new **ActB**s described here for their ability to catalyze the reduction of selected substrates with silanes. Initial testing was carried out in batch reactions with an exclusion of air and moisture, typically employing 1 mmol of the corresponding substrate, 20 mg of the **ActB** catalyst, 2 mL of solvent, and an excess of Et_3_SiH (or other silanes).

**Scheme 2:**
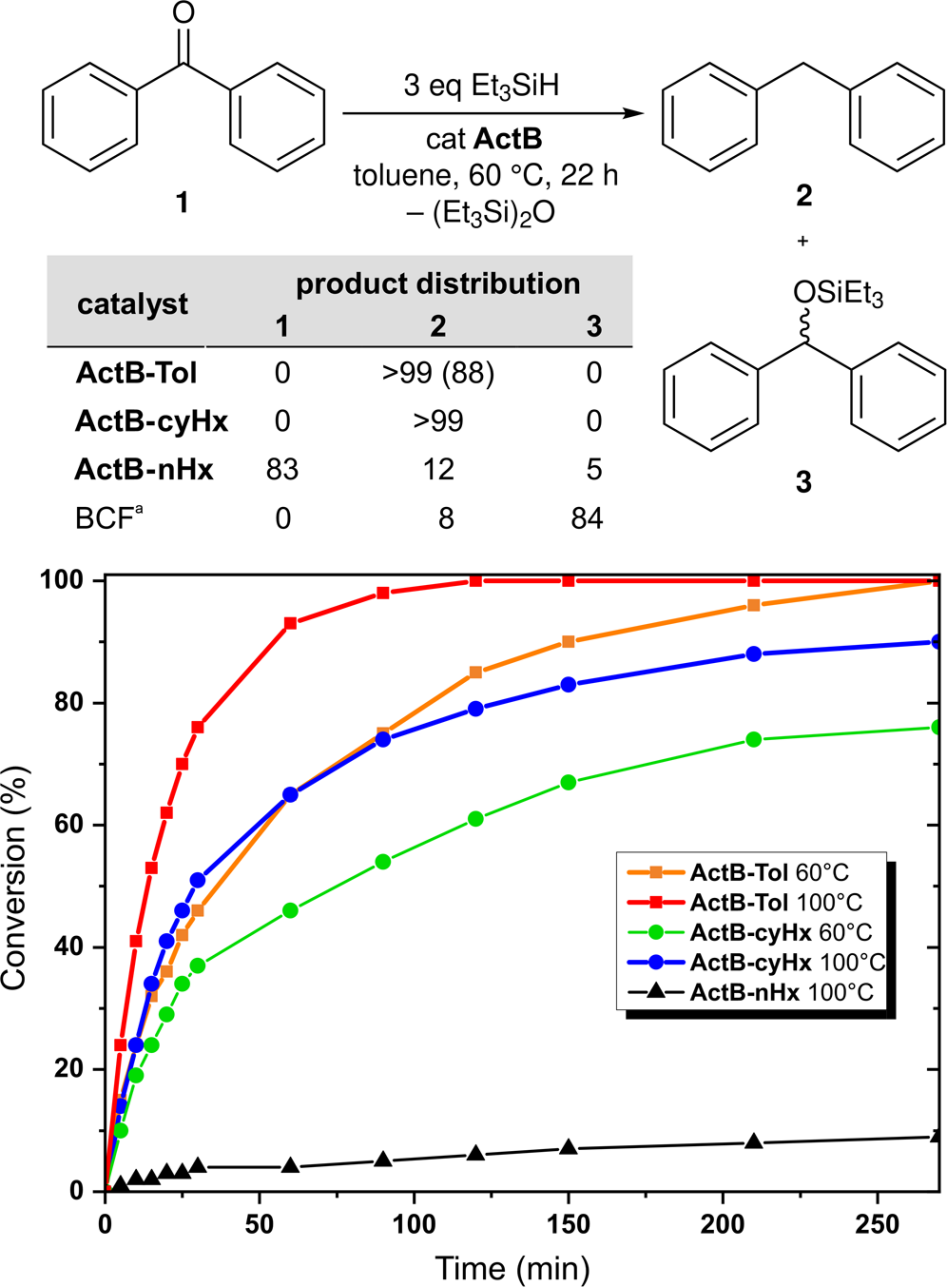
Deoxygenation of Benzophenone (**1**) Performed Using Different **ActB** Catalysts, Yields Determined by GC (Isolated Yield in Parentheses) 1 mol % B(C_6_F_5_)_3_ (BCF) used as a catalyst (reaction time 2 h). kinetic profiles (bottom) of the deoxygenation of **1** catalyzed by **ActB**s at 60 or 100 °C. Conversion of **1** determined by GC.

Benzophenone (**1**) as a model substrate was cleanly converted over both **ActB-Tol** and **ActB-*****cy*****Hx** materials to the corresponding deoxygenated product, diphenylmethane (**2**), by Et_3_SiH in toluene (Scheme 2), whereas the silyl ether intermediate **3** was only observed when using the **ActB-*****n*****Hx** material at the conditions applied. Using 1.5 equiv of Et_3_SiH with **ActB-Tol** gave incomplete conversion, while 3 equiv of Et_3_SiH afforded full conversion, and the product was subsequently isolated by column chromatography in an 88% yield. Expectedly, the siloxane (Et_3_Si)_2_O was identified as the only other product of deoxygenation. The kinetic profiles at 60 and 100 °C show that **ActB-Tol** is slightly more active than **ActB-*****cy*****Hx**, while **ActB-*****n*****Hx** exhibits only poor activity in this deoxygenation reaction.

These differences in performance are consistent with the observed relative LA strengths of the **ActB** materials, where **ActB-Tol** contained the most strongly acidic sites and **ActB-*****n*****Hx** contained the weakest or least accessible LA sites. To assess the relevance of these results, we performed a comparison test with the BCF catalyst. Thus, the exact same deoxygenation reaction was done but in the presence of 1 mol % of BCF as a catalyst. Here, the BCF converted **1** in 2 h, but in contrast to the **ActB-Tol** and **ActB-*****cy*****Hx** catalysts, BCF gave predominantly the silylated product **3** (84%) and only a small amount of **2**. We also tested the pristine *nido*-B_10_H_14_ as well as a sample prepared by thermolysis of *nido*-B_10_H_14_ without solvent at the same conditions as applied for the synthesis of **ActB** materials (dark brown powder without detectable porosity measured by Ar adsorption at 87 K). Neither of these, however, exhibited any catalytic activity in the above transformation (for details, see the SI).

**Scheme 3:**
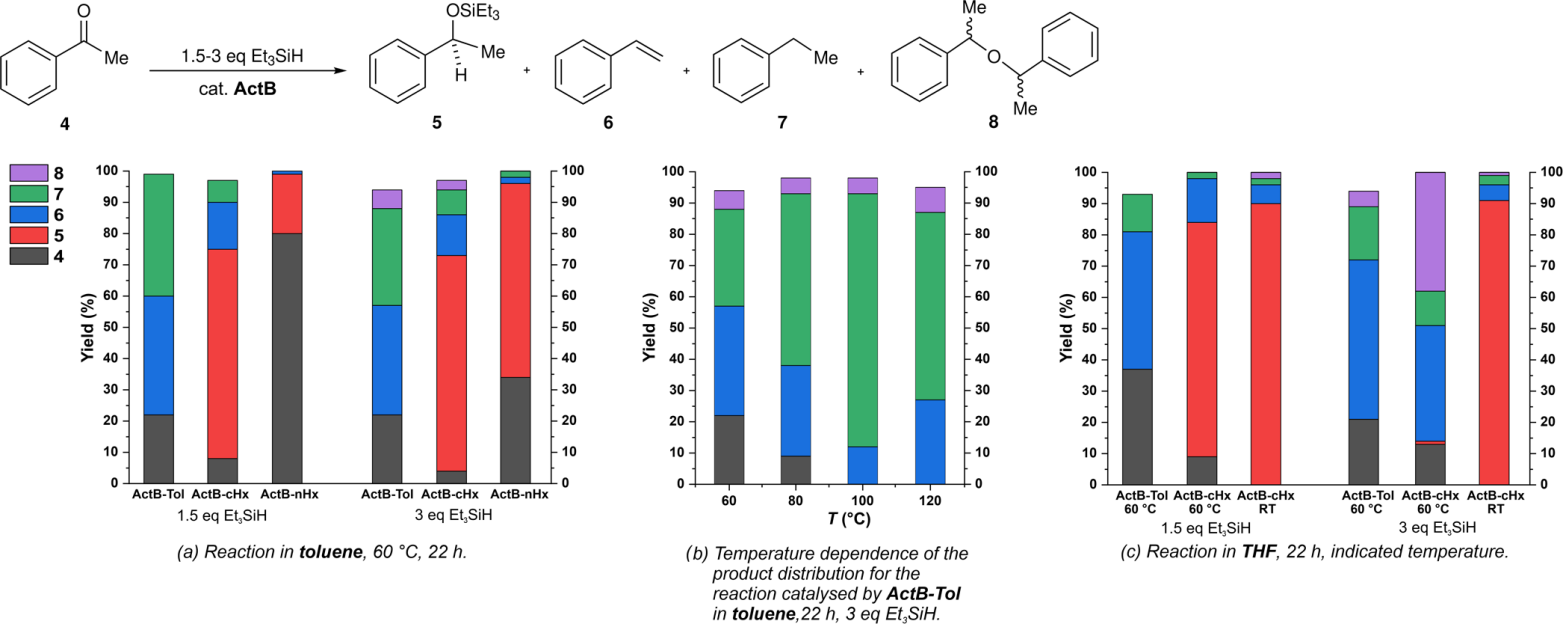
Hydrosilylation/deoxygenation of Acetophenone (**4**): (a) Screening of Different **ActB** Materials Using 1.5 or 3 equiv of Silane and Toluene as the Solvent; (b) Reaction Temperature Optimization Using **ActB-Tol** in Toluene; and (c) Variation of Reaction Conditions in THF Solvent. Yields Determined by GC

Next, we employed acetophenone (**4**) as a substrate for **ActB** catalytic assessment. In general, the reaction proceeded more slowly than for benzophenone, and the selectivity was greatly affected by conditions; see Scheme 3. **ActB-Tol** displayed a clear preference for deoxygenation under all tested conditions, with no detectable amounts of the silyl ether product **5**. Significant formation of styrene (**6**) was observed, accompanied by ethylbenzene (**7**). The relative amount of **7** was maximized at higher reaction temperatures (100 °C being the optimum) when an excess of silane was used (Scheme 3b), which also ensured full conversion of the starting material. **ActB-*****cy*****Hx**, in contrast, gave predominantly hydrosilylation product **5** irrespective of the amount of silane used. Prolonged reaction times with less available silane generally increased the amount of styrene formed. In contrast, **ActB-*****n*****Hx** was much less active but gave almost exclusively the hydrosilylation product **5**.

The styrene formation has been previously noted as a minor product in reactions of **4** catalyzed by BCF;^31^ its formation was significantly affected when 60 bar of H_2_ was applied combined with the presence of molecular sieves.^32^ In our hands, a control reaction employing BCF as a catalyst yielded only a mixture of ethylbenzene **7** (70%) and silyl ether **5** (25%), while no styrene was detected. Contrarily, the **ActB-Tol** catalyst under adjusted conditions (THF solvent, 60 °C; see Scheme 3c) afforded styrene (**6**) as the major product with a 65% selectivity (at an 89% conversion of **4**). It should be noted that the formation of olefins from ketones on acidic zeolite catalysts at temperatures above 250 °C yielding mixture of hydrocarbons is well-recognized.^33^

Reaction conditions were optimized for various silane hydrides, solvents, reaction times, temperatures, and type of **ActB** catalyst. The results can be summarized as follows: (a) best conversions were achieved with secondary silanes (Et_2_SiH_2_ or Ph_2_SiH_2_), yet it was still comparable with cheaper Et_3_SiH; (b) by means of conversion of acetophenone **4**, toluene appears to be the solvent of choice followed by THF; (c) selectivity is significantly affected by solvent and catalyst type: **ActB-Tol** showed increased selectivity toward the formation of styrene, whereas **ActB-cyHx** yields predominantly silyl ether **5**; (d) excess of silane favors the formation of symmetrical ether **8**; and (e) moderate temperature favors the formation of ethers **5** and **8**, whereas increased temperature deoxygenation products **6** and **7**. For a reaction overview, see Scheme 3, and for complete results, see the SI.

The effect of exposure of the **ActB-Tol** catalyst to air for a limited time (1 h) was studied. Interestingly, the activity does not seem to be affected much, which highlights the relative robustness of the **ActB** catalysts. Additionally, we tested the variability of catalytic properties of different batches of **ActB-Tol** in the hydrosilylation/deoxygenation of **1** and **4**. Results summarized in Table S16 confirmed the good reproducibility of activity as well as selectivity.

**Scheme 4:**
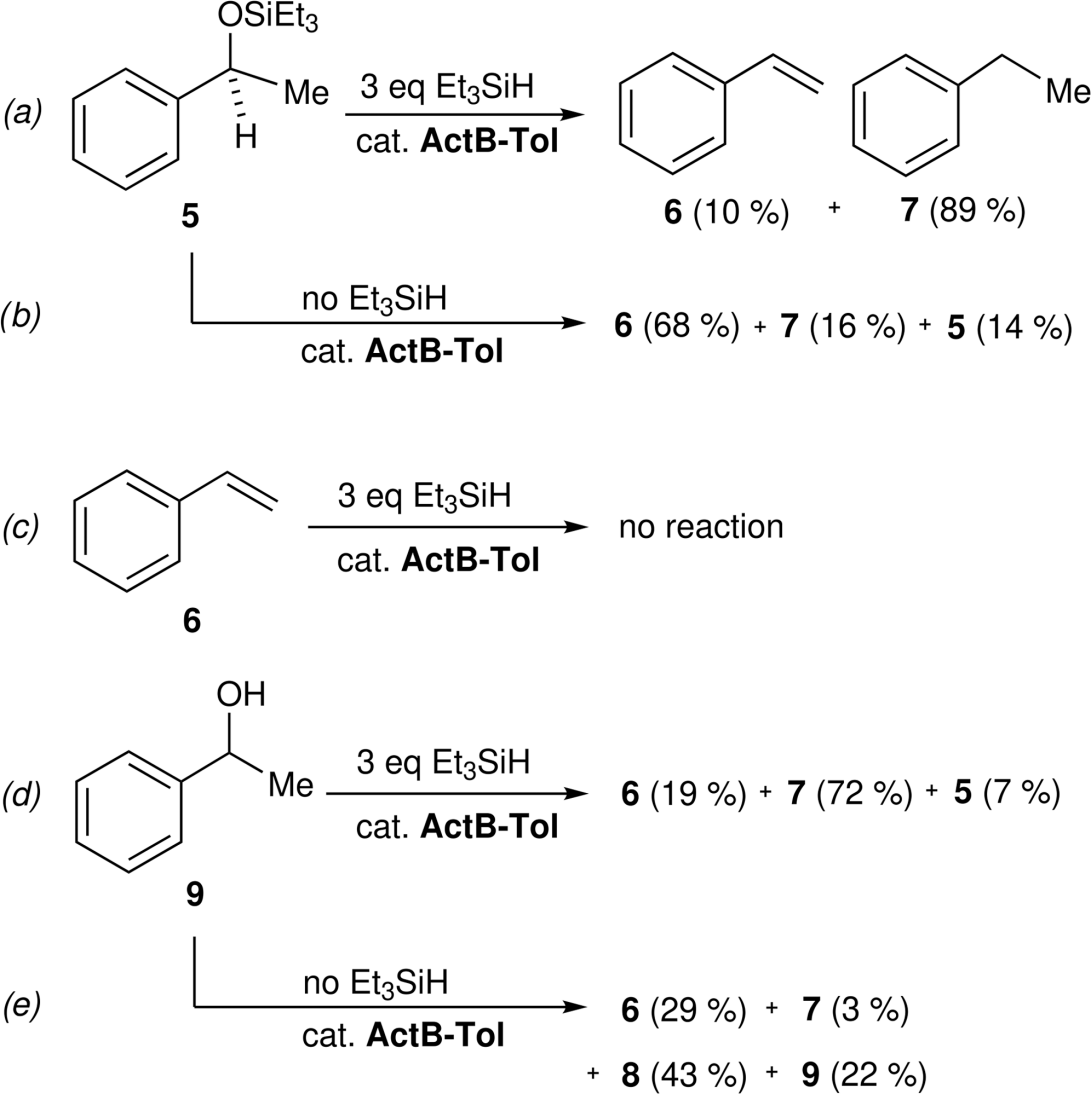
Reactions Involved in the Mechanism of 4-Hydrosilylation/Deoxygenation by **ActB-Tol**: (a) Deoxygenation of Silyl Ether **5** in the Presence of Silane, (b) without Silane, (c) Attempted Reaction of Styrene (**6**), (d) Reaction of Alcohol **9** in the Presence of Silane, and (e) without Silane. Conditions: 60 °C, 22 h, Toluene Solvent (1 mmol Substrate, 20 mg of **ActB**). GC Yields Given in Parentheses

In order to uncover the reaction mechanism of acetophenone transformation, we performed additional experiments employing products **5** and **6** and the related *rac*-1-phenylethanol (**9**) (Scheme 4). Silyl ether **5** is a plausible intermediate in the formation of both **6** and **7**, while **6** is the preferred product in the absence of silane as shown in (reaction *b*) in Scheme 4. When 3 equiv of Et_3_SiH was applied (reaction *a*) in (Scheme 4), **7** becomes the dominant product, as expected. Styrene (**6**), when exposed to the standard reaction conditions as depicted in (reaction *c*), is not converted to any other molecular products even in the presence of the silane. Alcohol **9** is also completely converted to a mixture of products **5**–**7** under the same conditions (reaction *d*). The formation of styrene under these conditions, and especially without the presence of silane (reaction *e*), caught our attention as this is formally a dehydration reaction.^34^ This transformation was further studied in the context of the technologically important dehydration of ethanol to ethylene; see below.

**Scheme 5:**
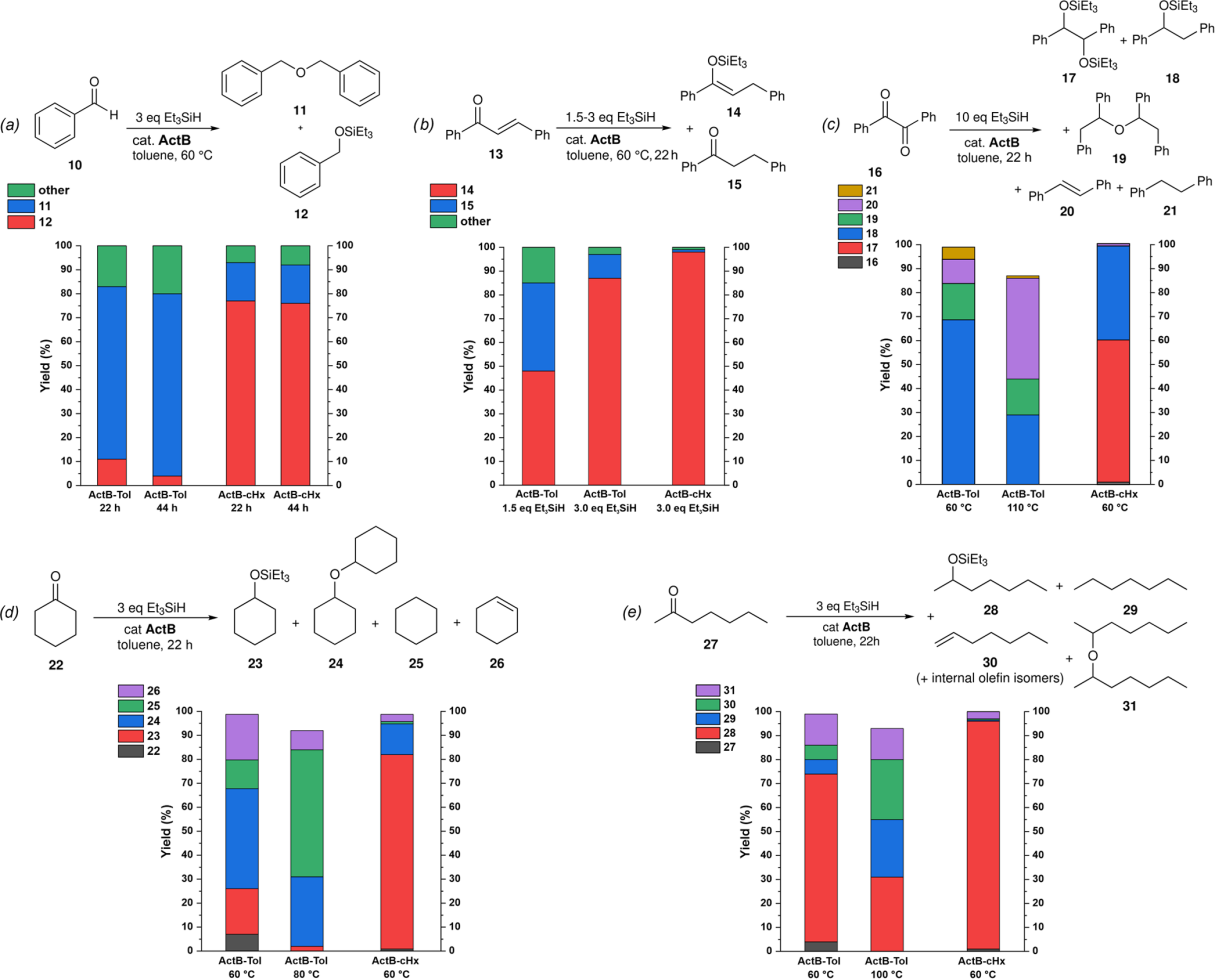
Hydrosilylation/Deoxygenation of (a) Benzaldehyde (**10**), (b) Trans-chalcone (**13**), (c) Benzil (**16**), (d) Cyclohexanone (**22**), and (e) 2-Heptanone (**27**), using **ActB-Tol** or **ActB-cyHx** as the Catalysts at Various Conditions (Silane Stoichiometry, Temperature, Time) as Indicated. All Reactions Performed in Toluene. Yields Determined by GC

**ActB-Tol** and **ActB-*****cy*****Hx** catalysts were further tested in the hydrosilylation/deoxygenation reaction of various other carbonyl-containing substrates (Scheme 5), namely, benzaldehyde (**10**), *trans*-chalcone (**13**), benzil (**16**), cyclohexanone (**22**), and 2-heptanone (**27**). It should be noted that in all cases, the reaction conversion was higher than 90%, often yielding a mixture of products, the composition of which is strongly dependent on the conditions used.

While each substrate behaves uniquely, it is evident that it follows similar trends as in the case of acetophenone: with **ActB-Tol**, higher temperature and excess of Et_3_SiH favoring deoxygenation products or the formation of ethers, while **ActB-*****cy*****Hx** affording predominantly hydrosilylated products. The formation of hydrosilylated products was obtained in much higher selectivities, which could be because of the milder conditions used. For full details of the optimization procedures and discussion of each substrate, see the SI.

#### Flow Conditions

**Figure 8:**
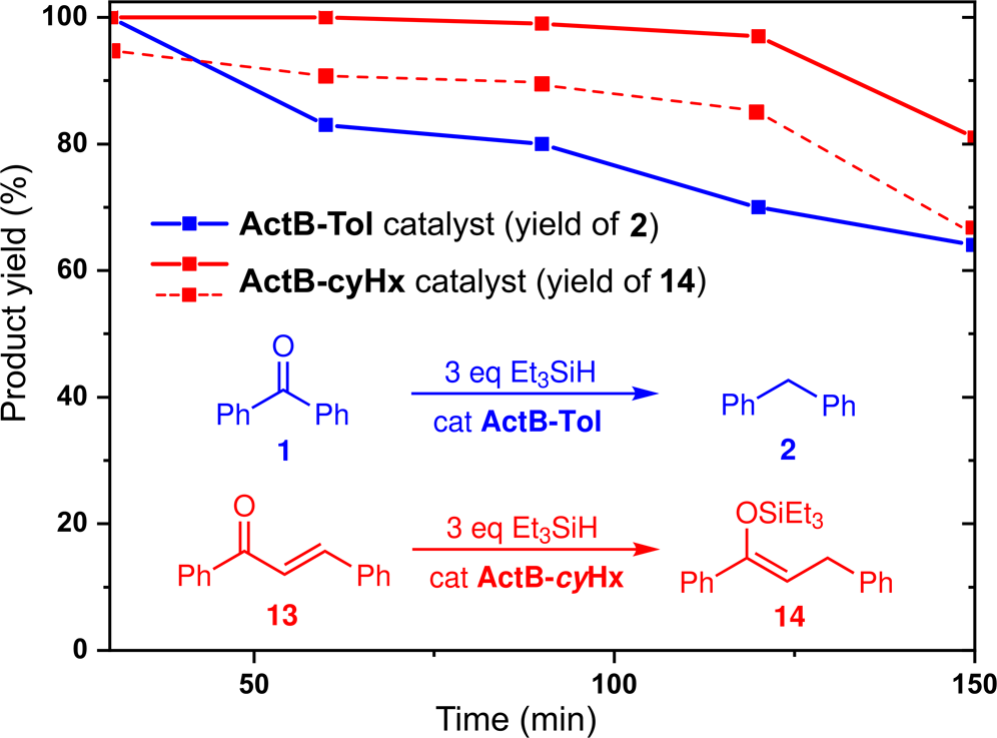
Time-dependence of the catalytic activity of **ActB-Tol** in benzophenone (**1**) deoxygenation and **ActB-cyHx** in trans-chalcone (**13**) 1,4-hydrosilylation under flow conditions (X-Cube, 100 °C, flow rate: 0.1 mL min^–1^—solid lines—or 0.2 mL min^–1^—segmented line, concentration of substrates 0.5 mM mL^–1^).

On the basis of the batch experiments, **ActB-Tol** and **ActB-*****cy*****Hx** catalysts were chosen for further investigation under flow conditions using a microfluidics-based flow reactor (X-Cube) in continuous flow mode. CatCart cartridges were loaded with the catalysts, and the catalyst bed was washed continuously with toluene solutions of the reactants and the resulting reaction mixtures were analyzed by GC. Based on the results from the preparative batch experiments described above, two reactions with high selectivity were chosen to test using flow conditions—the benzophenone (**1**) deoxygenation catalyzed by **ActB-Tol** and trans-chalcone (**13**) 1,4-hydrosilylation catalyzed by **ActB-*****cy*****Hx**. In both cases, the catalysts were able to operate in the flow mode, giving initially full conversion of the substrate to the corresponding product when running with a flow rate of 0.1 mL min^–1^. Gradual loss of activity was observed for the **ActB-Tol** catalyst, while the **ActB-*****cy*****Hx** also started losing its activity after ca. 2 h on stream (Figure 8). The same behavior was observed even when the flow rate was increased to 0.2 mL min^–1^. The leaching of boron moieties into the reaction mixture during the flow experiments was monitored by ICP-MS. The boron concentration in samples taken between 30 min and 2 h on stream was identical to the starting reaction mixture. For this reason, we conclude that the structure of **ActB**s is stable but its catalytic centers are gradually deactivated under hydrosilylation/deoxygenation reaction conditions.

#### Dehydration of Ethanol

As noted above, the reaction of 1-phenylethanol in the absence of silane produced mostly the products of dehydration (styrene and ether (**8**)). These results gave us reason to test **ActB**s for the industrially relevant gas-phase dehydration of ethanol and compare catalytic properties with traditional solid acid catalysts, namely, zeolite HZSM-5^ ^24b,24c,25a,35^^ and γ-alumina.^ ^25b,36^^ To validate the need for strong LA sites present in **ActB**s, we performed a blank experiment: impregnated activated charcoal with boric acid in B: C molar ratio is similar to **ActB**s.

**Figure 9:**
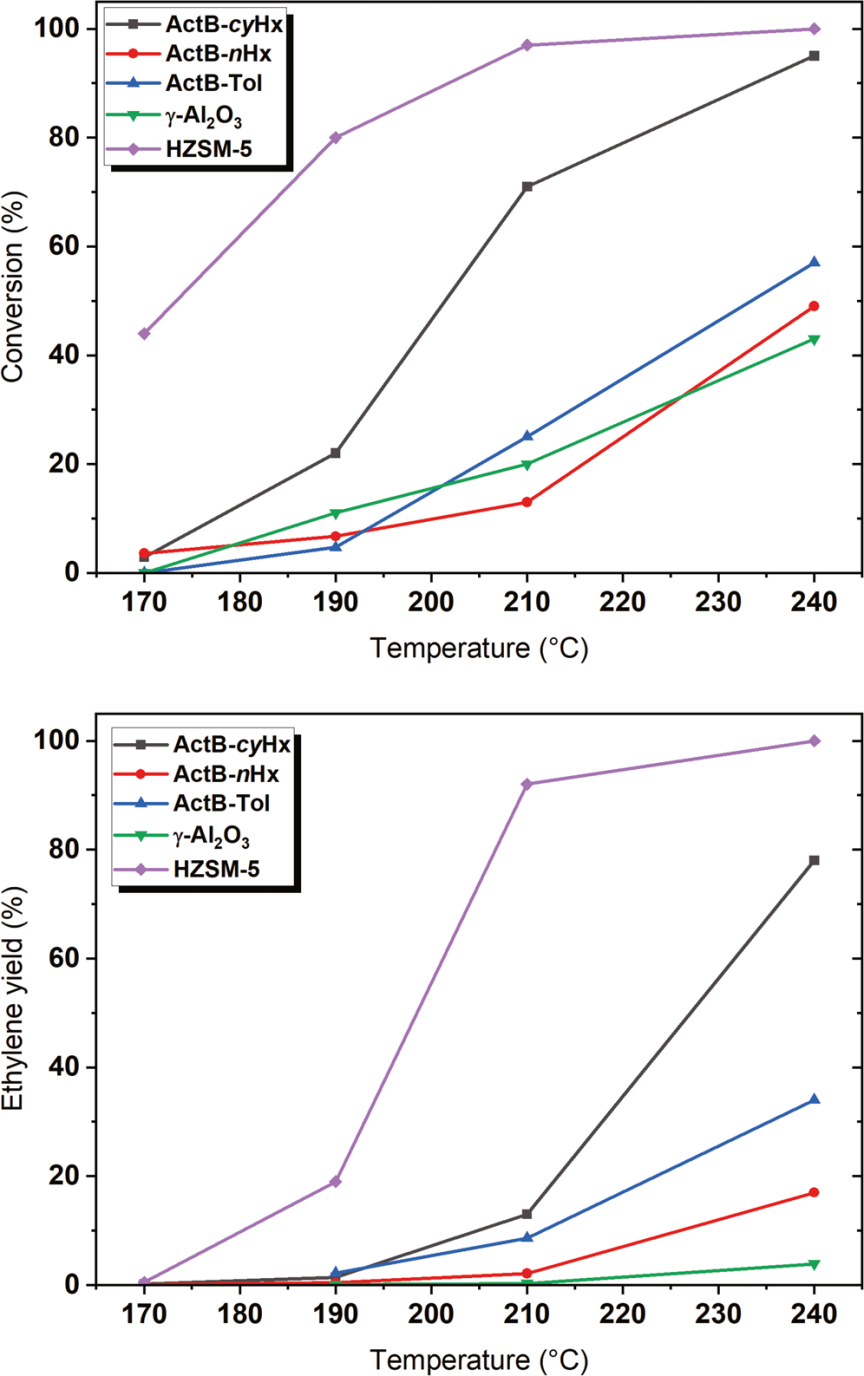
Ethanol conversion (top) and ethylene yield (bottom) during ethanol dehydration at 170, 190, 210, and 240 °C. Weight hour space velocity (WHSV) was kept for all measurements at 2.2 g g^–1^ h^–1^.

Ethanol dehydration was performed within the temperature range of 170 and 240 °C; see Figure 9. The conversion decreases in the following order: HZSM-5>**ActB-*****cy*****Hx**>**ActB-Tol** ≈ **ActB-*****n*****Hx** ≈ γ-Al_2_O_3_, with ethylene yields following a similar pattern. Thus, **ActB-*****cy*****Hx** achieved conversions over 90% and a ca. 80% yield of ethylene, while the control-activated charcoal–boric acid material was inactive throughout the whole temperature range.

The major products of the dehydration reactions were ethylene and diethyl ether, the ratio of which is dependent on the temperature. In this regard, **ActB**s behave similarly to many conventional catalysts. Only in the case of HZSM-5 were butenes observed as products of ethylene dimerization, which is in line with the literature.^24b^ In contrast to γ-Al_2_O_3_ and HZSM-5, all three **ActB**s produced only small amounts of ethane (**ActB-*****cy*****Hx** provided up to 3.6% ethane at 240 °C). We assume that ethane is formed via ethylene hydrogenation, with the hydrogen needed for this step coming from the B–H bonds in **ActB**s. This hypothesis is in agreement with the following observations: (i) ethane yield progressively decreased with time in all cases, (ii) addition of H_2_ gas to the reactant flow did not change the amount of formed ethane, and (iii) significant decrease in the intensity of B–H absorption bands in IR spectra (∼2550 cm^–1^) after the catalytic experiments; see Figure S33.

From all of the **ActB**s, **ActB-*****cy*****Hx** showed the most promising catalytic performance in ethanol dehydration. This behavior can be related to the high number of acidic sites as evidenced by ammonia TPD analysis and the materials’ porosity. **ActB-Tol** exhibited a similar porosity but a significantly lower number of acidic sites.

**Figure 10:**
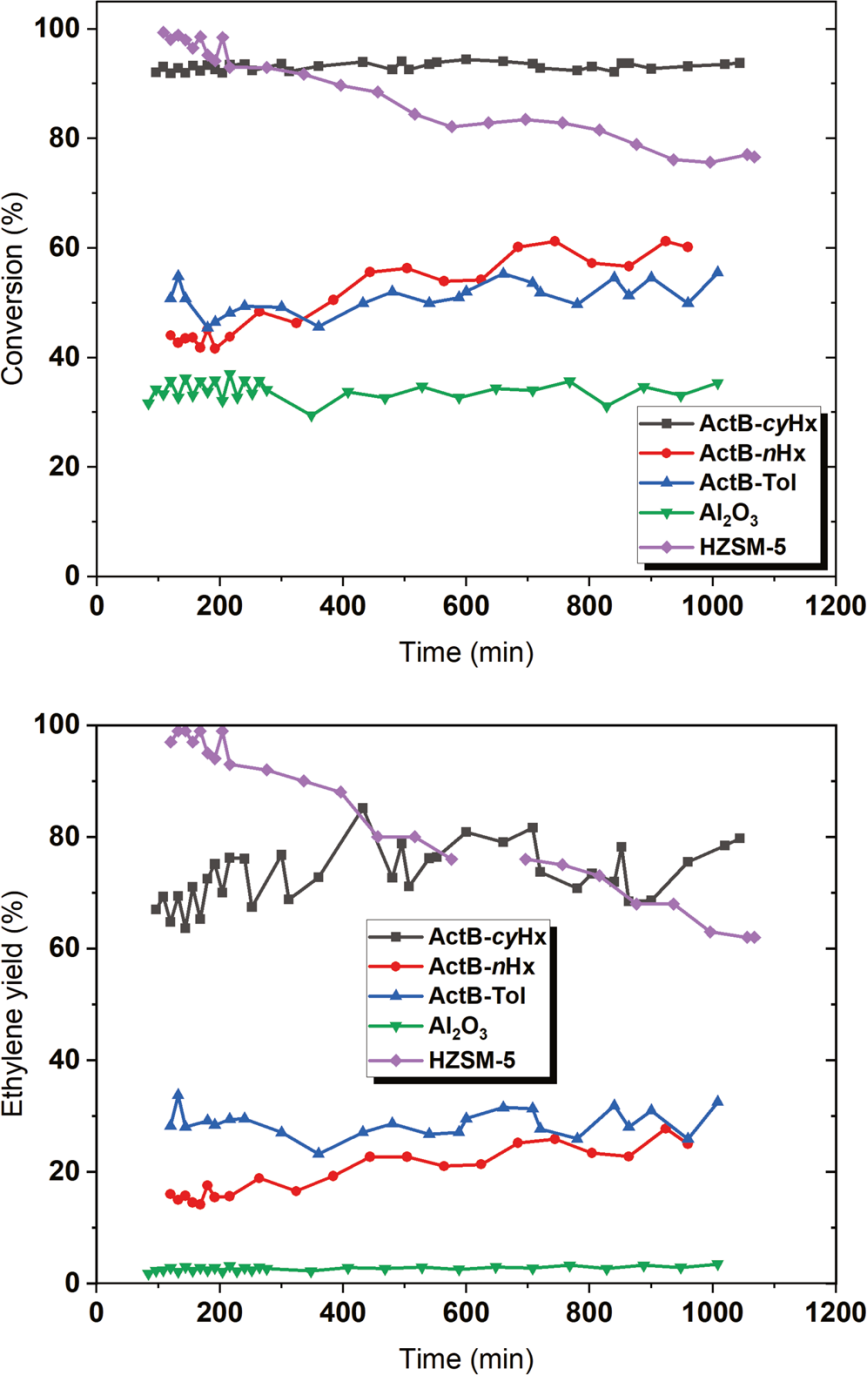
Ethanol conversion (top) and ethylene yield (bottom) during ethanol dehydration at 240 °C overnight (stability test). WHSV was kept at 4.4 g g^–1^ h^–1^ except for HZSM-5 due to its high activity. WHSV was set to 17.6 g g^–1^ h^–1^; for details, see the SI.

In an effort to probe the limits of the stability of **ActB**s, a 17 h time-on-stream experiment at 240 °C was conducted (Figure 10). Importantly, all **ActB**s proved to be stable, with **ActB-*****cy*****Hx** exerting a stable conversion of ∼93% and an ethylene selectivity of ∼78%. HZSM-5, in particular, has been shown to suffer from ethylene oligomerization,^35b^ coking, and deactivation.^37^ In line with the literature, HZSM-5 proved to be unstable with ethanol conversion decreasing from 98 to 76% and ethylene yield dropping from 99 to 62%. For a detailed discussion, all ethanol conversions, selectivities, and carbon balances, see the SI.

## Conclusions

We have demonstrated that controlled cothermolyses of decaborane with aromatic or aliphatic hydrocarbons in strictly anaerobic conditions give highly porous and halide-free solid materials, **ActB**s, containing high numbers of Lewis acidic (LA) sites in their structures. These sites give these new materials a level of catalytic activity that approaches that of the well-established molecular borane LA B(C_6_F_5_)_3_ but with the added benefit of being a heterogeneous catalyst, allowing the use in flow reactors. The strength and number of LA sites in **ActB**s seem to be controllable by the nature of the hydrocarbon linkers incorporated into the material. We proved **ActB**s to be valuable heterogeneous catalysts in batch as well as flow conditions for hydrosilylation/deoxygenation reactions of various carbonyl substrates using silanes as the reductants. More importantly, not only can **ActB**s be used for analogous reactions as catalyzed by B(C_6_F_5_)_3_ but they also show some unique reactivity, e.g., the formation of styrene from acetophenone. In general, the toluene-based **ActB-Tol** preferred deoxygenation pathways in comparison to the other two catalysts with aliphatic linkers, which were, in most cases, more selective toward the silylation products.

The potential for dehydration reactions was studied in detail in ethanol dehydration in the gas phase, which revealed the capability of **ActB** catalysts to operate efficiently under flow conditions at 240 °C with excellent stability over 17 h on stream. The activity and selectivity for ethylene exceed those of commonly used γ-alumina catalysts. Further investigation of the effects of the catalytic material composition on its LA and consequently catalytic properties as well as utilization in other types of catalytic reactions is currently under way.

## Supporting Information Available

Full experimental details of syntheses and catalytic experiments, additional characterization data for the **ActB** materials, detailed results of catalytic investigations (product distribution, characterization data) including an extended discussion. The authors have cited additional references within the Supporting Information.^38^ (PDF)
