# Supplementary material for: “Activated Borane”: A Porous Borane Cluster Polymer as an Efficient Lewis Acid-Based Catalyst
Source: ACS Catal. 2023 Oct 30;13(22):14614–26. doi: 10.1021/acscatal.3c04011 (PMC10660343; doi:10.1021/acscatal.3c04011)
Supplement: Supplementary file 1 — cs3c04011_si_001.pdf [file cs3c04011_si_001.pdf]

Supporting Information

"Activated Borane" – A Porous Borane Cluster polymer as an efficient Lewis acid-based catalyst

Martin Lamač,[a] Béla Urbán,[a] Michal Horáček,[a] Daniel Bůžek,[b] Lucie Leonová,[c] Aleš Stýskalík,[c] Anna Vykydalová,[b] Karel Škoch,[b] Matouš Kloda,[b] Andrii Mahun,[d] Libor Kobera,[d] Kamil Lang,[b] Michael G. S. Londesborough,[b] Jan Demel[b*]*

[a] Dr. M. Lamač, Dr. B Urbán, Dr. M. Horáček
Department of Molecular Electrochemistry and Catalysis
J. Heyrovsky Institute of Physical Chemistry of the Czech Academy of Sciences
Dolejškova 2155, 182 00, Prague 8, Czech Republic
E-mail: martin.lamac@jh-inst.cas.cz

[b] Dr. D. Bůžek, Dr. A. Vykydalová, Dr. K. Škoch, Dr. M. Kloda, Dr. K. Lang, Dr. M. G. S. Londesborough, Dr. J. Demel
Department of Materials Chemistry
Institute of Inorganic Chemistry of the Czech Academy of Sciences
Husinec-Řež 1001, 250 68, Řež, Czech Republic
E-mail: demel@iic.cas.cz

[c] L. Leonová, Dr. A. Stýskalík
Department of Chemistry
Masaryk University
Kotlářská 2, 611 37, Brno, Czech Republic

[d] A. Mahun, Dr. L. Kobera
Department of Structural Analysis
Institute of Macromolecular Chemistry of the Czech Academy of Sciences
Heyrovského nám. 2, 162 06, Prague 6, Czech Republic

Table of Contents

General Information for Experiments 6

Materials 6

Instrumental methods 6

Synthesis of ***ActBs*** 8

Adsorption of probe molecules 8

Characterisation of **ActB** materials 9

Figure S1 Absorption spectra of ***ActB-Tol**, **ActB-cyHx*** *and* ***ActB-nHx*** 9

Figure S2 Tauc plots of ***ActB-Tol**, **ActB-cyHx*** *and* ***ActB-nHx*** *for obtaining indirect band gap energies* 9

Figure S3. SEM images of ***ActB-Tol**, **ActB-cyHx*** *and* ***ActB-nHx*** 10

Figure S4. Pore size distribution for ***ActB*** *materials.* 11

Table S1. Data points for adsorption isotherms of Ar for ***ActB-Tol*** 11

Table S2. Data points for adsorption isotherms of Ar for ***ActB-cyHx*** 13

Table S3. Data points for adsorption isotherms of Ar for ***ActB-nHx*** 14

Table S4. Data points for adsorption isotherms of CO_2 *for* ***ActB-Tol*** 15

Table S5. Data points for adsorption isotherms of CO_2 *for* ***ActB-cyHx*** 17

Table S6. Data points for adsorption isotherms of CO_2 *for* ***ActB-nHx*** 19

Table S7. Elemental analysis of ***ActB-Tol**, **ActB-cyHx*** *and* ***ActB-nHx*** 20

Figure S5. Batch-to-batch comparison of Ar adsorption isotherms of ***ActB-Tol*** 21

Figure S6. Batch-to-batch comparison of pore size distribution of ***ActB-Tol*** 21

Figure S7. Batch-to-batch comparison of FTIR for ***ActB-Tol*** 22

Figure S8. ^{11}B *3Q/MAS NMR spectra of* ***ActB-Tol**, **ActB-cyHx*** *and* ***ActB-nHx*** 22

*Figure S9. Comparison of ^{13}C CP/MAS NMR spectra for as-prepared and TEPO loaded **ActB-Tol, ActB-cyHx**, and **ActB-nHx** samples* 23

Detailed discussion of TGA/DTA results 23

*Table S8. Parameters obtained from TGA records for **ActB-Tol, ActB-cyHx** and **ActB-nHx** with and without adsorbed benzene and pyridine* 24

*Figure S10. TGA records for as-prepared samples **ActB-Tol, ActB-cyHx** and **ActB-nHx*** 25

*Figure S11. TGA records for sample **ActB-Tol*** 25

*Figure S12. TGA records for sample **ActB-cyHx*** 26

*Figure S13. TGA records for sample **ActB-nHx*** 26

*Figure S14. Selected values of m/z for **ActB-Tol** and **ActB-Tol** adsorbed with benzene* 27

*Figure S15. Selected values of m/z for **ActB-Tol** and **ActB-Tol** adsorbed with pyridine* 27

*Figure S16. Selected values of m/z for **ActB-cyHx** and **ActB-cyHx** adsorbed with benzene* 28

*Figure S17. Selected values of m/z for **ActB-cyHx** and **ActB-cyHx** adsorbed with pyridine* 28

*Figure S18. Selected values of m/z for **ActB-nHx** and **ActB-nHx** adsorbed with benzene* 29

*Figure S19. Selected values of m/z for **ActB-nHx** and **ActB-nHx** adsorbed with pyridine* 29

Hydrosilylation/deoxygenation catalysis 30

General Details. 30

General procedure for the hydrosilylation/deoxygenation reactions 30

*Reaction of benzophenone (**1**) and kinetic profile screening* 30

*Figure S20. ^{1}H NMR spectra of a reaction mixture of **1** catalysed by **ActB-Tol**.* 31

*Reaction of acetophenone (**4**)* 32

*Table S9. Product distribution in PhAc (**4**) reaction using different **ActB** catalysts* 32

*Table S10. Product distribution in PhAc (**4**) reaction catalysed by $B(C_6F_5)_3$ (BCF)* 33

*Table S11. **ActB-Tol** catalysed PhAc (**4**) silylation/deoxygenation using different silanes* 33

*Table S12. Solvent screening for the PhAc (**4**) silylation/deoxygenation* 33

*Table S13. Temperature optimization for the PhAc (**4)** silylation/deoxygenation* 34

*Table S14. Effect of air-exposure on the PhAc (**4**) silylation/deoxygenation* 34

*Table S15. Product distribution in PhAc (**4**) silylation/deoxygenation reaction* 34

*Figure S21. ^{1}H NMR spectra of a reaction mixture of **4** catalysed by **ActB-Tol*** 35

*Figure S22. ^{1}H NMR spectra of a reaction mixture of **4** catalysed by **ActB-cyHx*** 35

*Table S16. Product distribution in PhAc (**4**) reaction using different batches of **ActB-Tol*** 37

*Reaction of benzaldehyde (**10**)* 37

*Table S17. Hydrosilylation of benzaldehyde (**10**) by Et_3SiH catalysed by **ActB** materials* 37

*Figure S23. ^{1}H NMR spectra of a crude reaction mixture of **10** catalysed by **ActB-Tol*** 38

*Figure S24. ^{1}H NMR spectra of a crude reaction mixture of **10** catalysed by **ActB-cyHx*** 38

*Reaction of trans-chalcone (**13**)* 39

*Table S18. Hydrosilylation of trans-chalcone (**13**) by Et_3SiH catalysed by **ActB** materials* 39

*Figure S25. ^{1}H NMR spectra of a reaction mixture of **13** catalysed by **ActB-Tol** at 60 °C* 39

*Figure S26. ^{1}H NMR spectra (500 MHz) of compound **15** in $CDCl_3$* 40

*Figure S27. ^{1}H NMR spectra of a reaction mixture of **13** catalysed by **ActB-cyHx** at 60 °C* 40

*Reaction of benzil (**16**)* 40

*Table S19. Hydrosilylation/deoxygenation of **16** by Et_3SiH catalysed by **ActB** materials* 41

*Figure S28. ^{1}H NMR spectra of a reaction mixture of **16** catalysed by **ActB-Tol** at 60 °C* 41

*Figure S29. ^{1}H NMR spectra of a reaction mixture of **16** catalysed by **ActB-cyHx** at 60 °C* 42

*Reaction of cyclohexanone (**22**)* 42

*Table S20. Hydrosilylation/deoxygenation of **22** by Et_3SiH catalysed by **ActB** materials* 42

*Figure S30. ^{1}H NMR spectra of a reaction mixture of **22** catalysed by **ActB-Tol** at 60 °C* 43

*Reaction of 2-heptanone (**27**)* 43

*Table S21. Hydrosilylation/deoxygenation of **27** by Et_3SiH catalysed by **ActB** materials* 44

*Figure S31. ^1H NMR spectra of a reaction mixture of **27** catalysed by **ActB-Tol** at 60 °C* 44

*Figure S32. ^1H NMR spectra of a reaction mixture of **27** catalysed by **ActB-cyHx** at 60 °C* 44

General procedure for the hydrosilylation/deoxygenation reaction in the flow reactor............45

Detailed discussion of the hydrosilylation/deoxygenation substrate scope 45

*Scheme S1. Hydrosilylation of benzaldehyde (**10**) catalysed by **ActB** materials* 45

*Scheme S2. Hydrosilylation of **13** catalysed by **ActB** materials* 46

*Scheme S3. Hydrosilylation/deoxygenation of **16** catalysed by **ActB** materials* 47

*Scheme S4. Hydrosilylation/deoxygenation of **22** catalysed by **ActB** materials* 48

*Scheme S5. Hydrosilylation/deoxygenation of **27** catalysed by **ActB** materials* 48

Ethanol dehydration (gas flow reactor)..49

*Figure S33. IR spectra of **ActB-cyHx** before, and after ethanol dehydration catalysis* 49

Figure S34. Ethylene selectivity in ethanol dehydration reaction at diferent temperatures.....50

Figure S35. Ethylene selectivity at 240 °C overnight (stability test)..50

Table S22. Ethanol conversion, selectivities and yields in ethanol dehydration reaction 51

Table S23. Ethanol conversion and selectivity at 240 °C overnight (stability test). 52

General Information for Experiments

Materials. *Nido*-$B_{10}H_{14}$ (Katchem, Czech Republic) was purified by column chromatography (silicagel, hexane) followed by recrystallization from hexane before use. Toluene, *n*-hexane and cyclohexane (all Lach:Ner, Czech Republic) were dried over Na/benzophenone and freshly distilled before use. Triethylphosphineoxide (Merck) was used as received. The synthesis of **ActB**s were done under Ar using standard Schlenk technique and Ar-filled glove box (PureLab, Inert corp.).

Instrumental methods. Thermogravimetric analysis with mass spectrometry (TGA/MS) was used to determine the thermal stability and the ability to adsorb benzene (B) and pyridine (P) at RT and atmospheric pressure. Measuring of samples were performed on a Netzsch STA449 F1 Jupiter (TGA) coupled with Agilent Technologies 5977B MSD (MS). Samples were measured in an open alumina pan and the purge gas was argon with a flow of 50 ml min^{-1}. The temperature program was in the range 35 – 800 °C with a heating rate of 10 °C min^{-1}. The mass of samples were ~ 4 mg. Indium, zinc and aluminium were used to calibrate the temperature. Two parallel runs were done for each sample.

Individual selected fragments of gas analysis were collected in multiple ion detection mode as intensity (A). Values of m/z were chosen depending on the composition of studied material (mass-to-charge ratios).

Solid-state ^{1}H, ^{11}B, and ^{31}P NMR spectra were recorded at 16.4 T using a Bruker Avance Neo spectrometer. A 3.2 mm cross-polarization magic angle spinning (CP/MAS) probe was used for the corresponding NMR experiments carried out at Larmor frequencies of $\nu(^{1}H)$ = 700.13 MHz, $\nu(^{11}B)$ = 224.63 MHz, and $\nu(^{31}P)$ = 283.42 MHz respectively. Solid-state ^{13}C NMR spectra were acquired at 11.7 T using a Bruker AVANCE III HD spectrometer equipped with a 3.2 mm CP/MAS probe operating at Larmor frequency of $\nu(^{13}C)$ = 125.76 MHz. All ssNMR spectra were recorded at the MAS rotation rate of 20 kHz. The ^{1}H NMR chemical shift was calibrated using adamantane (^{1}H: 1.85 ppm), the ^{11}B NMR spectra were referenced to H_3BO_3 in D_2O (^{11}B: 19.5 ppm), the ^{31}P

NMR chemical shift was referenced to solid $CaHPO_4$ (^{31}P: -0.6 ppm), and the ^{13}C NMR chemical shift was calibrated using α-glycine (^{13}C: 176.03 ppm, carbonyl signal) as external standards respectively.

The ^{1}H MAS experiments were performed using a 2.27 µs 90° pulse with 64 scans and 2s recycle delay. The ^{11}B MAS spectra were recorded by application of 1.7 µs 90° pulse with 32 scans and 2s recycle delay. The ^{11}B 3Q/MAS NMR spectra[1] were acquired with spectral width in both frequency dimensions of 62.5 kHz. The indirect detection period t_1 consisted of 96 increments, each made of 512 scans. The ^{13}C CP/MAS NMR experiments were carried out using a 90° pulse with a length of 2.27 µs, 1.5 ms cross-polarization contact time with 4096 scans and 2 s recycle delay. In the case of ^{31}P MAS NMR experiments, a 3 µs 90° pulse was used with 5 s recycle delay and 1024 scans. The SPINAL-64 decoupling sequence was used in ^{31}P and ^{11}B NMR experiments, and rCW^{ApA} sequence[2] was used in ^{13}C NMR experiments in order to remove heteronuclear interactions.

All the samples were packed into ZrO_2 rotors and subsequently kept under an inert atmosphere. All experiments were performed at 298 K.[3] Bruker TopSpin 3.2 pl5 software package was used for the processing of the spectra.

Scanning electron microscopy was performed using a JSM-6510 microscope (JEOL). The content of hydrogen and carbon was determined by standard combustion technique, the boron content by ICP-MS (Agilent 7900 ICP-MS instrument equipped with an Ar burner, an ORS 4 collision reaction cell and a hyperbolic quadrupole mass analyser with an orthogonal detection system). Before the ICP-MS measurement, 0.5 mL of liquid samples after the catalytical experiments (filtered through Whatman 0.2 µm PTFE microfilter) or 10 mg of **ActB** material were evaporated under 40°C in the dryer. Then the residue was added to a solution of HCl (12 mL), HNO_3 (4 mL) and HF (4 mL) and decomposed under microwave irradiation. The decomposed liquid sample was diluted to 30 mL and measured by ICP-MS. To determinate of boron background, the reaction mixture used for catalytical experiments, which was not in contact with **ActB** catalyst, was used (blank sample). Diffusion reflectance spectra were measured in the range from 1500 to 200 nm on a PerkinElmer Lambda750 spectrometer equipped with a 10 cm integration sphere. Recorded reflectance values were transformed into Kubelka-Munk units. FTIR spectra were recorded on a

Nicolet Avatar spectrometer using an ATR technique with a Si crystal on samples enclosed in a cuvette under inert atmosphere when necessary.

TPD was measured using a AMI-300 from ALTAMIRA Instruments Inc. instrument. Prior to measurement, the samples were treated with a flow of He (30 mL min^{-1}) at 100°C for 3 h. Saturation with NH_3 was done at 40°C in a flow of 10% of NH_3 in He for 1h followed by flushing with pure He at 60°C for 1.5 h. During TPD measurements, each sample was heated up to 750°C with a ramp of 3°C min^{-1} and the desorbing NH_3 was detected by a thermal conductivity detector. Simultaneously, a MS detector was used to distinguish between NH_3, water and residual solvents.

Synthesis of ActBs: A stainless steel autoclave (Berghof BR-300) was charged in an Ar-filled glovebox (PureLab, Inert corp., USA) with 2.0 g of *nido*-$B_{10}H_{14}$ and 100 mL of dry toluene, cyclohexane or *n*-hexane. The autoclave was heated for 24 h at 250°C with a 1 h heating ramp. After cooling to RT, the autoclave was opened in the glovebox and the dark suspension was transferred to glass frit that was inserted into an Ar-filled Soxhlet extractor and was extracted for 48 h using the same solvent as with which the synthesis was done. The solvents were removed under vacuum at 100 °C to yield **ActB-Tol** as a black powder (1.5 g, 19.6% boron utilization), **ActB-*cy*Hx** as a black powder (2.7 g, 52.5% boron utilization), or **ActB-*n*Hx** as a brown powder (1.9 g, 42.9% boron utilization). All samples were stored under an Ar atmosphere.

Adsorption of TEPO: 5 mg of triethylphosphine oxide was dissolved in 0.1 mL of dry toluene and mixed with 50 mg of **ActB**. The formed thick slurry was left to evaporate in a glovebox at RT.

Adsorption of probe molecules: 15 mg of **ActB** in an open vial was placed into a larger vial containing 1 mL of the probe molecules (benzene or pyridine) the larger vial was closed tightly and the system was left to equilibrate for 48 h.

Characterisation of ActB materials

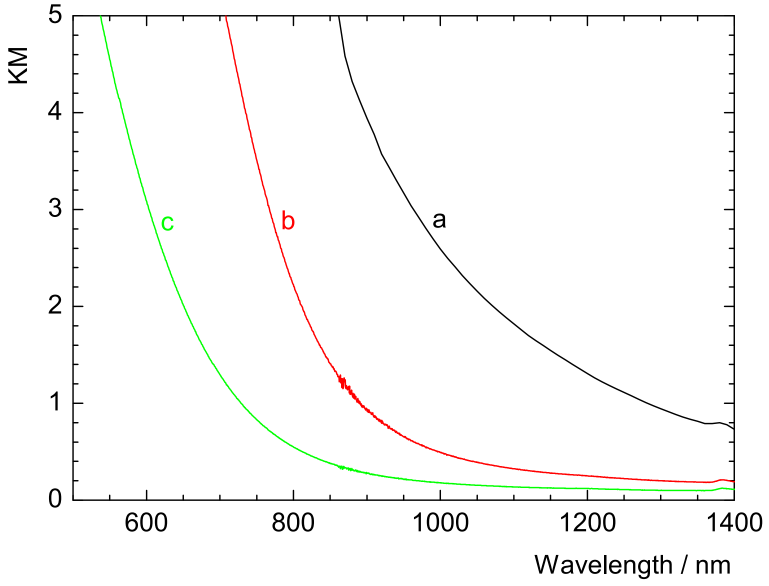

Figure S1. Absorption spectra of **ActB-Tol** (a)**, ActB-*cy*Hx** (b) and **ActB-*n*Hx** (c) in the Kubelka−Munk units.

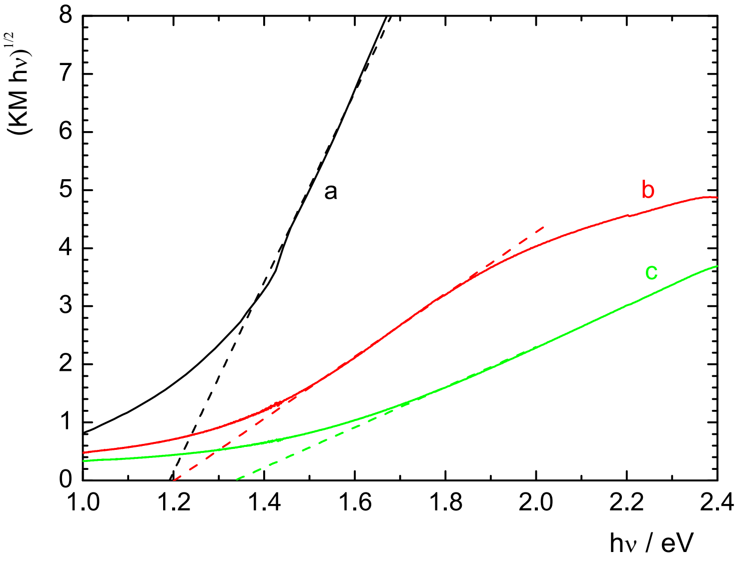

Figure S2. Tauc plots of **ActB-Tol** (a), **ActB-*cy*Hx** (b) and **ActB-*n*Hx** (c) for obtaining indirect band gap energies. Dashed lines represent linear fits extrapolated to the *x*-axis.

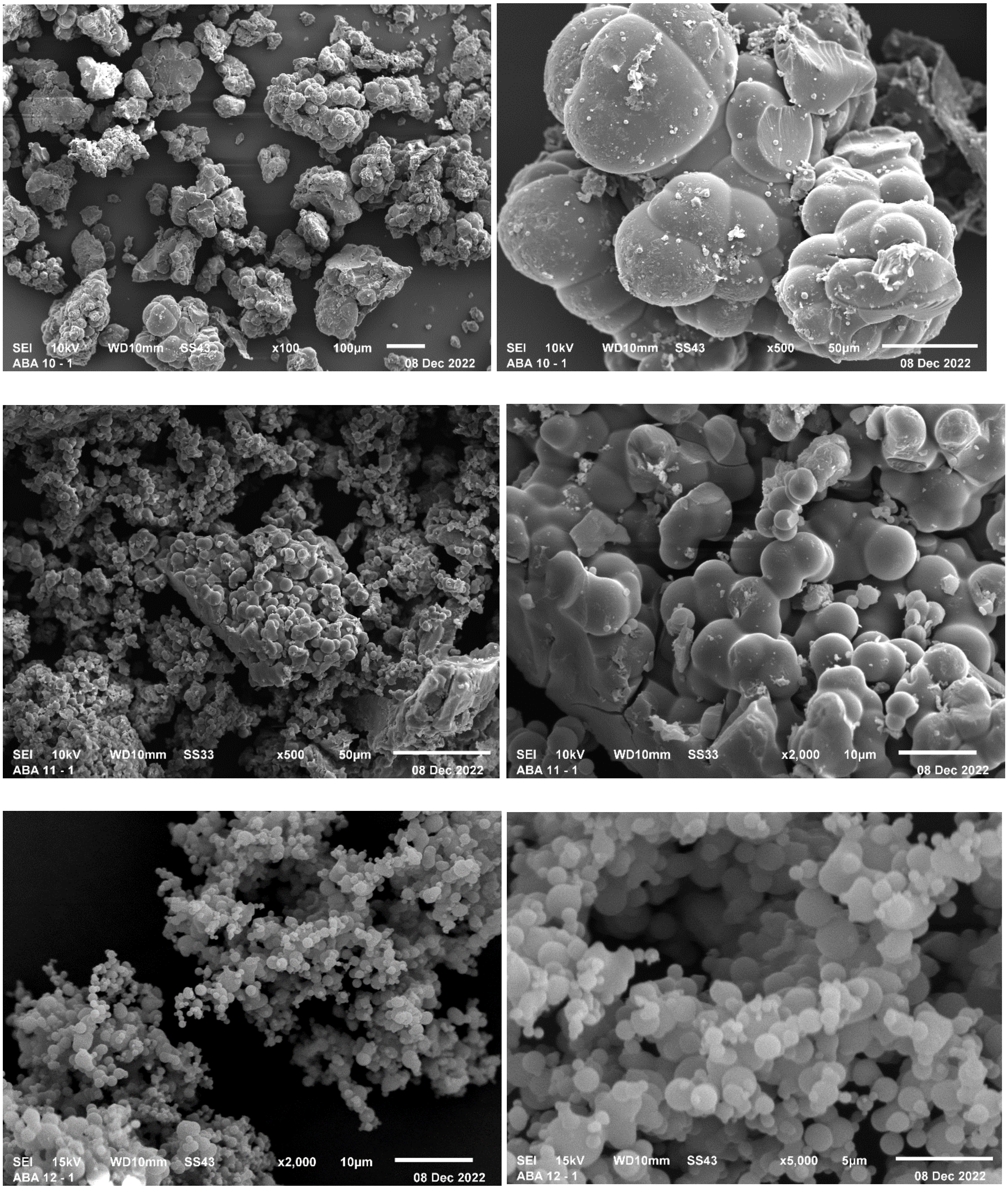

Figure S3. SEM images of **ActB-Tol** (top), **ActB-*cy*Hx** (middle) and **ActB-*n*Hx** (bottom). Left are overall views and right detailed snapshots.

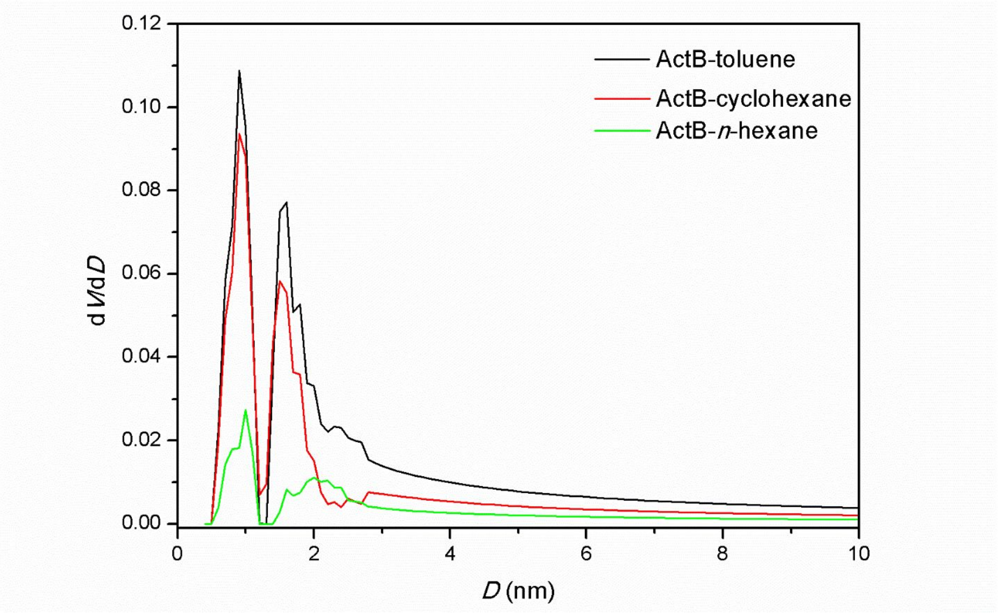

Figure S4. Pore size distribution calculated by MDFT method from adsorption isotherms of argon measured at 87 K.

Table S1. Argon adsorption and desorption isotherm data points for **ActB-Tol**.

**Adsorption**		**Desorption**	
P/P_0	V_{ads} (cm^3g^{-1} STP)	P/P_0	V_{ads} (cm^3g^{-1} STP)
$1.68 \cdot 10^{-5}$	20.30089	0.971205	371.8784
0.000103	40.53803	0.916447	367.7084
0.000317	60.6531	0.862994	363.0141
0.000996	80.18203	0.810706	357.8235
0.001807	99.64275	0.758498	352.0828
0.003221	118.3264	0.706933	346.5518
0.0057	135.9762	0.655689	340.5686
0.009616	152.0012	0.603823	334.2143
0.015803	165.2838	0.553422	326.8287
0.027329	182.361	0.503323	319.7502

0.042489	195.6866	0.453726	311.9349
0.059878	205.8188	0.404871	303.5757
0.078131	213.8654	0.349631	292.541
0.09793	220.719	0.299974	281.5913
0.121162	226.3026	0.252866	268.6508
0.166342	236.4352	0.207466	255.6724
0.209253	245.1528	0.162728	242.1703
0.255175	254.3671	0.120981	226.5434
0.300536	262.2285	0.078251	213.9071
0.3468	270.0655		
0.390574	276.9041		
0.434911	284.0592		
0.478022	289.657		
0.5226	296.3915		
0.56535	303.6367		
0.608762	309.9018		
0.650468	316.1613		
0.691753	322.3533		
0.731431	329.1961		
0.770667	335.3996		
0.822352	342.6926		
0.871769	351.976		
0.920704	361.7635		
0.971205	371.8784		

Table S2. Argon adsorption and desorption isotherm data points for **ActB-CyHx**.

**Adsorption**		**Desorption**	
P/P_0	V_{ads} (cm^3g^{-1} STP)	P/P_0	V_{ads} (cm^3g^{-1} STP)
$2.63\cdot10^{-5}$	17.68768	0.97249	232.7369
0.000141	35.32096	0.930302	230.7399
0.000398	52.81573	0.888194	228.7365
0.000919	70.12988	0.847372	226.6764
0.001969	87.14024	0.807634	224.5894
0.003966	103.4004	0.767213	222.3416
0.007597	118.6054	0.726832	220.1865
0.014478	131.6426	0.687315	217.9375
0.029879	147.4085	0.648541	215.6639
0.051686	158.7936	0.610048	213.3389
0.078051	167.031	0.572137	210.8498
0.109556	172.9519	0.533302	208.4817
0.152888	179.1793	0.495913	205.8855
0.197727	184.0817	0.458284	203.7326
0.243931	188.7083	0.421276	201.4899
0.28903	192.6706	0.384289	199.0023
0.334973	195.8773	0.348045	196.7005
0.379771	198.6984	0.308267	194.0132
0.423003	201.606	0.2714	191.1217
0.466476	204.2264	0.234814	187.7955
0.5076	206.5441	0.199373	184.2466
0.549748	209.0275	0.165197	180.5251
0.591594	211.3886	0.132507	176.2503
0.632597	213.5925	0.101584	171.4537
0.672737	216.0998	0.06273	162.2441

0.711792	218.0215		
0.76396	220.8687		
0.816449	223.2022		
0.869259	226.2826		
0.921005	229.1142		
0.97249	232.7369		

Table S3. Argon adsorption and desorption isotherm data points for **ActB-*n*Hx**.

**Adsorption**		**Desorption**	
P/P_0	V_{ads} (cm^3g^{-1} STP)	P/P_0	V_{ads} (cm^3g^{-1} STP)
0.000106	8.99146	0.959759	79.5808
0.000505	17.81165	0.920423	78.08102
0.002132	25.73438	0.876267	76.7078
0.005389	32.52527	0.838758	75.67995
0.014819	34.99409	0.798678	74.63678
0.034678	39.13442	0.756691	73.53321
0.055943	42.00253	0.712596	72.30302
0.07795	44.01171	0.674363	71.12972
0.099596	45.80363	0.635047	69.93621
0.124114	47.0692	0.597337	68.81978
0.1609	48.58855	0.559004	67.7
0.198028	49.80871	0.521495	66.44822
0.234393	51.34239	0.484106	64.92514
0.268408	54.17998	0.446135	63.40107
0.301741	56.4455	0.408887	61.80381
0.336238	58.3483	0.371538	60.04115
0.369932	59.96381	0.328547	57.9241
0.401799	61.49796	0.290114	55.6553

0.433344	62.85915	0.252505	53.07996
0.46483	64.19311	0.214734	51.40063
0.493986	65.3003	0.177928	49.6214
0.523463	66.53033	0.141704	47.7957
0.552699	67.51591	0.106403	46.155
0.591333	68.64389	0.071384	43.41225
0.629967	69.77571		
0.657456	70.64418		
0.695749	71.74384		
0.721732	72.60627		
0.761269	73.6546		
0.801509	74.71112		
0.84195	75.76299		
0.881909	76.86315		
0.921386	78.1115		
0.959759	79.5808		

Table S4. CO_2 adsorption and desorption isotherm data points for **ActB-Tol**.

**Adsorption**		**Desorption**	
P/P_0	V_{ads} (cm^3g^{-1} STP)	P/P_0	V_{ads} (cm^3g^{-1} STP)
0.001516	14.20841	0.556613	424.1022
0.003967	25.87589	0.536138	417.8487
0.007186	35.40579	0.515901	410.7565
0.010183	45.52284	0.495491	403.7906
0.015129	59.39037	0.475752	396.6155
0.020421	72.31109	0.456132	389.4774
0.025713	84.63216	0.436966	381.8727
0.031405	95.95562	0.417996	374.523

0.037097	106.5995	0.399209	366.6058
0.042941	116.7452	0.381201	359.4051
0.048742	126.3325	0.362696	351.5018
0.054629	135.5971	0.344905	343.6734
0.060473	144.693	0.327341	335.6807
0.067615	153.4524	0.309106	327.5929
0.074844	161.782	0.291618	319.558
0.082095	170.2527	0.274476	311.2822
0.089659	177.7395	0.256901	302.4153
0.096975	185.5465	0.239737	294.0353
0.10442	192.4561	0.222596	284.6822
0.11943	206.0639	0.205854	275.2917
0.134603	218.8994	0.189048	265.1795
0.14892	231.8503	0.171646	253.4592
0.163292	243.0501	0.154331	240.6725
0.179178	253.9398	0.137665	227.3803
0.194231	263.6142	0.121616	212.367
0.20988	272.4969	0.10679	197.0951
0.225171	280.8312	0.092592	180.8692
0.23964	287.7826		
0.253882	294.9572		
0.268978	302.0452		
0.283523	308.4873		
0.298457	314.7462		
0.312006	320.4821		
0.324938	326.7854		
0.339104	332.755		
0.357945	340.1401		

0.369838	345.4266		
0.382695	351.1282		
0.395161	356.3465		
0.407174	361.0933		
0.417952	365.8388		
0.436707	373.4948		
0.455905	380.9023		
0.475482	389.4105		
0.495286	397.9269		
0.514538	405.4414		
0.535435	414.2099		
0.556613	424.1022		

Table S5. CO_2 adsorption and desorption isotherm data points for **ActB-CyHx**.

**Adsorption**		**Desorption**	
P/P_0	V_{ads} (cm^3g^{-1} STP)	P/P_0	V_{ads} (cm^3g^{-1} STP)
0.00188	6.63735	0.554676	190.0769
0.004359	12.33076	0.539212	188.2889
0.007554	17.06207	0.523834	186.182
0.010725	21.80496	0.508413	183.9774
0.017077	30.84097	0.493413	181.7925
0.02397	39.18161	0.480795	179.8224
0.031373	46.59735	0.467365	177.7267
0.038786	53.39003	0.454217	175.6967
0.04661	60.17963	0.441328	173.6262
0.054748	66.30165	0.427887	171.5044
0.063773	72.34328	0.414998	169.4039
0.073124	77.83823	0.402499	167.376

0.082441	83.27071	0.38961	165.1819
0.092051	88.18065	0.375628	162.8621
0.101812	92.86817	0.362945	160.6602
0.116682	99.18336	0.350889	158.5486
0.131616	105.2269	0.338314	156.3673
0.146225	111.1929	0.325707	153.9951
0.160521	116.135	0.3139	151.828
0.175001	120.8463	0.301758	149.7815
0.189665	125.0892	0.289843	147.53
0.20461	129.1038	0.278133	145.2048
0.21818	132.5672	0.266587	143.0099
0.232108	135.759	0.255234	140.697
0.246166	138.8265	0.243395	138.2219
0.260613	141.8064	0.232	135.7342
0.273296	144.2293	0.220626	133.1277
0.285958	146.6904	0.209339	130.3961
0.299637	149.1774	0.198376	127.7692
0.312233	151.4493	0.187349	124.8016
0.325977	153.7276	0.176191	121.7722
0.344504	156.9989	0.164623	117.9887
0.356809	159.0414	0.153422	114.2999
0.369589	161.0768	0.14247	110.3551
0.382175	163.2278	0.131075	106.0913
0.400951	166.1287	0.120578	101.5501
0.420052	169.2298	0.110199	97.07956
0.438676	172.085	0.100113	92.3787
0.458794	175.2669	0.090503	87.38997
0.477841	178.1925		

0.498056	181.325		
0.516583	184.1036		
0.535922	187.1756		
0.554676	190.0769		

Table S6. CO_2 adsorption and desorption isotherm data points for **ActB-*n*Hx**.

**Adsorption**		**Desorption**	
P/P_0	V_{ads} (cm^3g^{-1} STP)	P/P_0	V_{ads} (cm^3g^{-1} STP)
0.002184	7.35972	0.568626	182.91126
0.005437	13.94188	0.537913	177.62274
0.009707	19.63823	0.507514	172.22024
0.013452	25.86501	0.476466	166.58934
0.020594	33.63805	0.44649	161.22503
0.027596	41.36614	0.416643	155.57511
0.035907	47.98373	0.386547	149.62305
0.045896	53.28418	0.358562	143.87556
0.054802	59.52724	0.330035	137.90619
0.065938	64.73345	0.301476	131.84417
0.077907	69.1786	0.273924	125.66825
0.089713	73.6478	0.246598	119.36433
0.101639	77.91918	0.218894	112.8692
0.124733	86.63688	0.191721	105.95004
0.148498	94.0289	0.163789	98.36512
0.17315	101.01964	0.137113	90.48773
0.197597	107.51019	0.111974	81.82049
0.221806	113.60171	0.087484	72.80393
0.245289	119.06148		
0.268112	124.34011		

0.291531	129.69222		
0.314192	134.59558		
0.336647	139.28787		
0.358389	143.84089		
0.379686	148.10517		
0.400345	152.30475		
0.421102	156.29317		
0.450883	161.84662		
0.481174	167.41273		
0.510328	172.73378		
0.539396	177.8855		
0.568626	182.91126		

Table S7. Elemental analysis of **ActB**s.

	C (w%)[a]	H (w%)[a]	B (w%)[b]
**ActB-Tol**	59.72	5.61	23.1
**ActB-*cy*Hx**	39.38	7.29	34.4
**ActB-*nHx***	31.28	7.47	39.9

[a] determined by standard combustion technique; [b] determined by ICP-MS

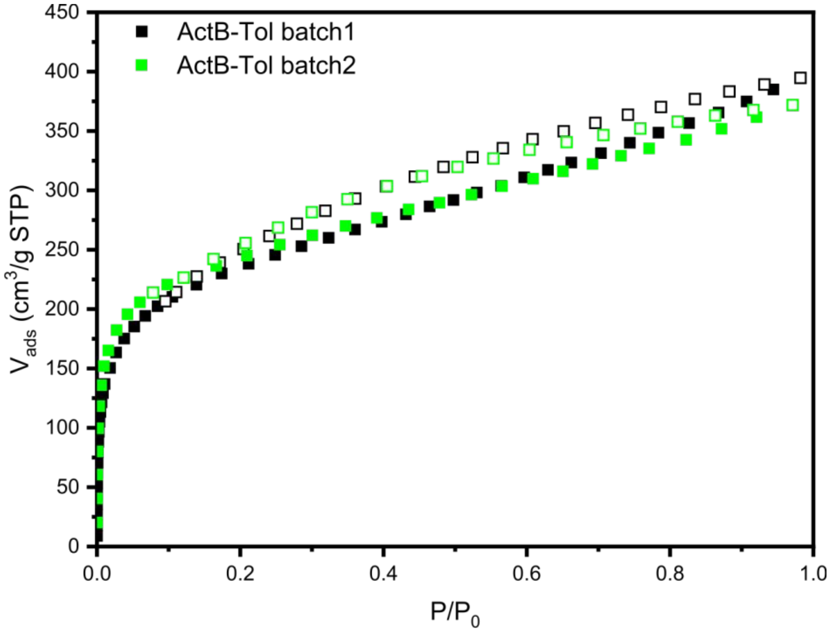

Figure S5. Adsorption isotherms for two different batches of **ActB-Tol** synthesized by the same procedure.

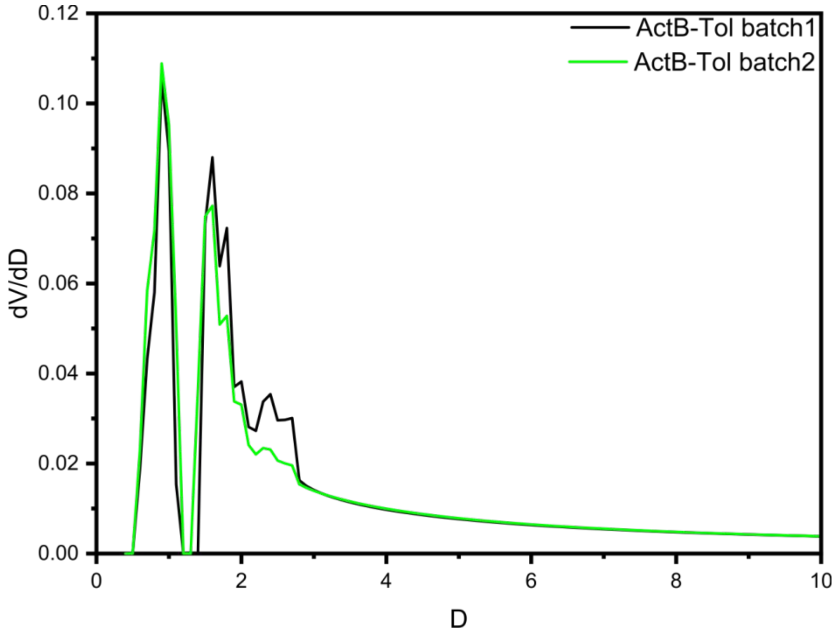

Figure S6. Pore size distribution calculated by MDFT method from adsorption isotherms of argon for two different batches of **ActB-Tol**.

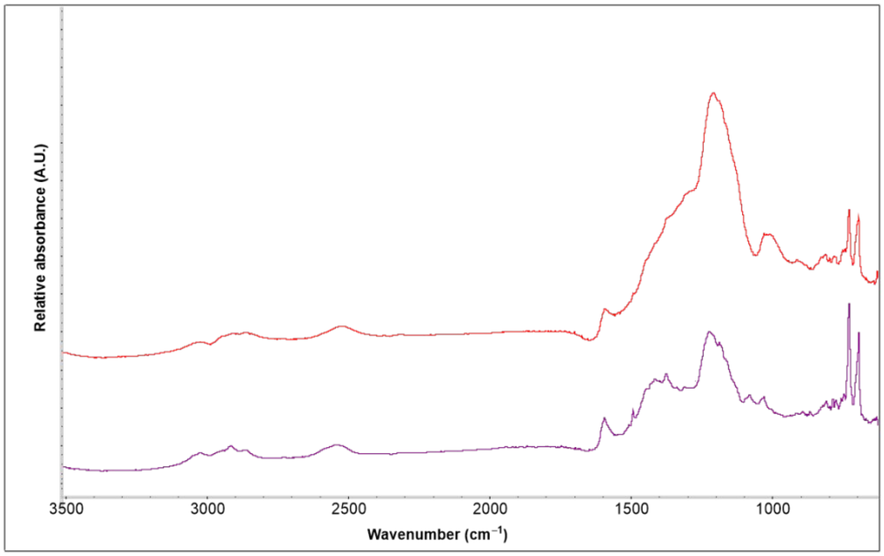

Figure S7. FT IR (ATR-Si) spectra for two different batches of **ActB-Tol**.

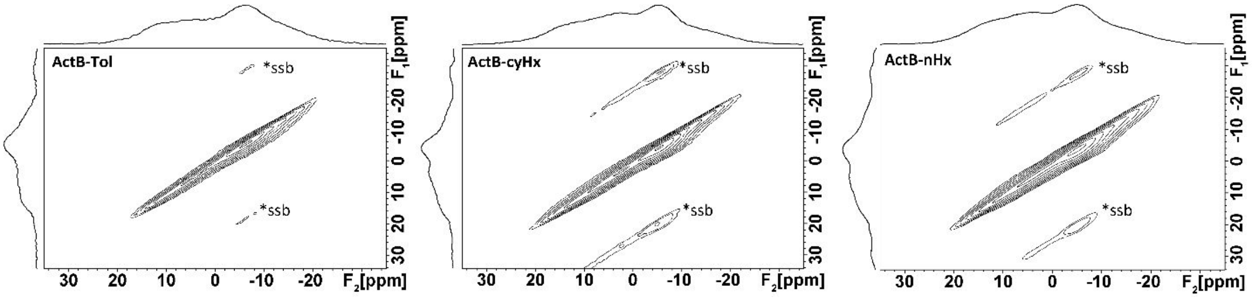

Figure S8. ^{11}B 3Q/MAS NMR spectra of **ActB-Tol** (left), **ActB-*cy*Hx** (middle) and **ActB-*n*Hx** (right).

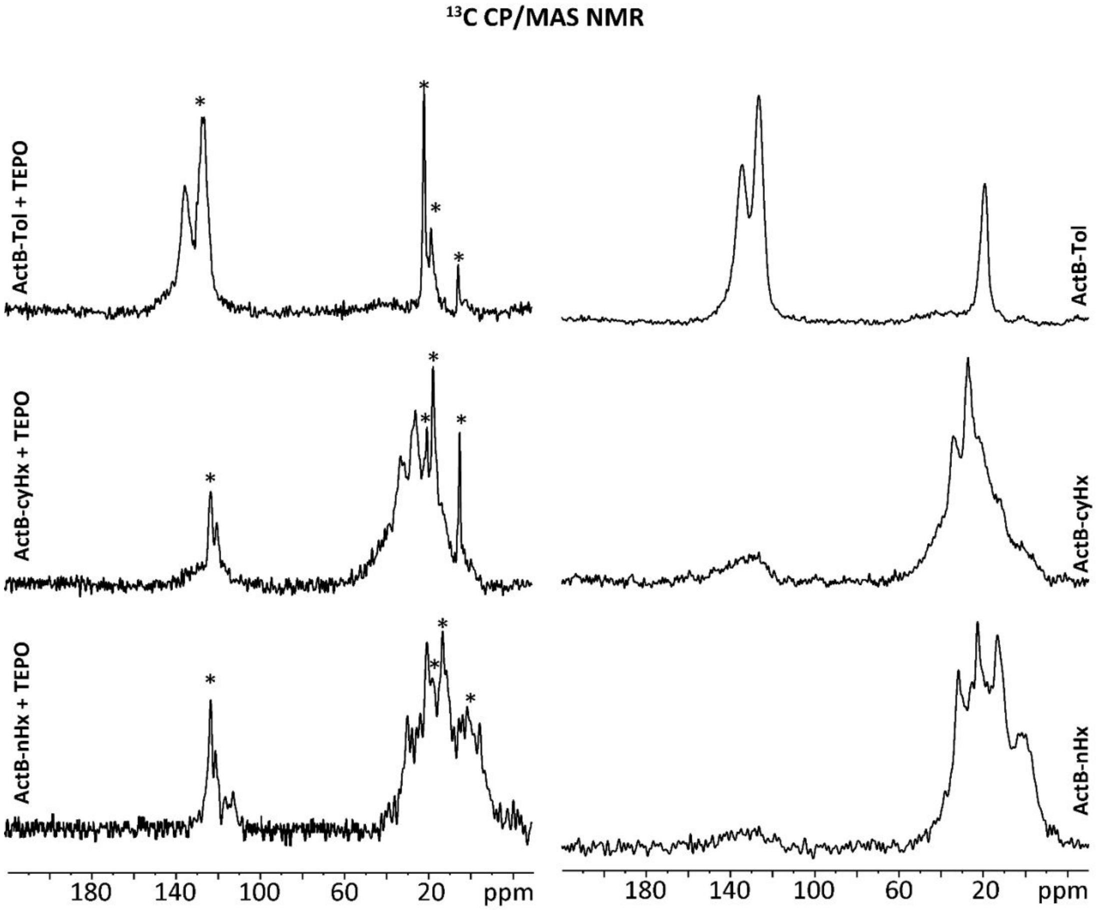

Figure S9. Experimental ^{13}C CP/MAS NMR spectra of **ActB-Tol**, **ActB-*cy*Hx**, and **ActB-*n*Hx** samples with loaded TEPO (left-hand column) and as-prepared samples (right-hand column). The asterisk denotes signals originated from TEPO and residual toluene from the TEPO adsorption (see above).

Detailed discussion of TGA/DTA results

The thermal stabilities of **ActB**s were studied by TGA coupled with MS (Figures S7-16). The lowest stability was obtained for sample **ActB-Tol** where the onset of the main degradation step (T_{onset}) is around 245 °C while for **ActB-*cy*Hx** and **ActB-*n*Hx** are higher at 319 °C and 322 °C, respectively. The mass residue (R) of the samples at their T_{onset} for each sample is more or less comparable, see Table S2.

Table S8. Parameters obtained from TGA records for **ActB-Tol, ActB-*cy*Hx** and **ActB-*n*Hx** with and without adsorbed benzene and pyridine.

Sample	T_{onset} (°C)[a]	$R_{244.4°C}$ (%)[b]	$R_{318.5°C}$ (%)[b]	$R_{321.7°C}$ (%)[b]	$R_{800°C}$ (%)[c]
**ActB-Tol**	244.4	98.71	-	-	89.95
**ActB-Tol** + benzene	261.5	93.02	-	-	80.46
**ActB-Tol** + pyridine	84.9/-	84.67	-	-	70.99
**ActB-*cy*Hx**	318.5	-	99.01	-	91.60
**ActB-*cy*Hx** + benzene	308.1	-	96.70	-	89.62
**ActB-*cy*Hx** + pyridine	80.8/325.5	-	89.72	-	83.46
**ActB-*n*Hx**	321.7	-	-	98.59	89.44
**ActB-*n*Hx** + benzene	332.5	-	-	96.25	88.68
**ActB-*n*Hx** + pyridine	78.8/348.3	-	-	93.44	87.47

[a] T_{onset} is the onset temperatures of the main degradation step. [b] R_T is the mass residues at onset temperatures for pure materials [c] $R_{800°C}$ is the mass residues at 800 °C.

To assess the proclivity of **ActB**s to adsorb neutral and basic molecules, benzene and pyridine were pre-adsorbed onto each sample at room temperature followed by TGA measurement (Figures S8-10). The difference between the adsorption of neutral molecule (benzene) and basic molecule (pyridine) can be used as a proxy for the estimation of the number of acid sites accessible to medium-sized molecules. It can be clearly seen (Figures S11-16) that the intensities of m/z values correspond with TG records so that first is desorbed the adsorbate (benzene or pyridine), followed by the degradation products of **ActB**s. This allows us to compare the mass residue at T_{onset} for the pure samples with adsorbed samples, see Table S8.

Interestingly, the amount of adsorbed pyridine was in the order **ActB-Tol** > **ActB-*cy*Hx** > **ActB-*n*Hx**. It correlates well with the specific surface area determined by Ar adsorption and not with the ammonia TPD. This could be due to the inaccessibility of part of the channel system to medium-sized molecules.

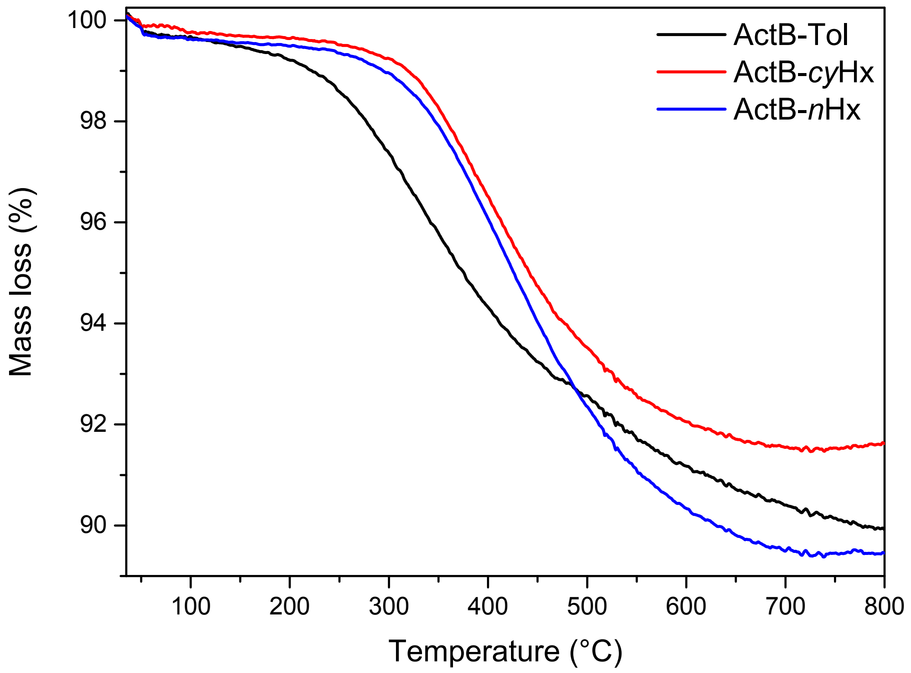

Figure S10. TGA records for as-prepared samples **ActB-Tol**, **ActB-*cy*Hx** and **ActB-*n*Hx**.

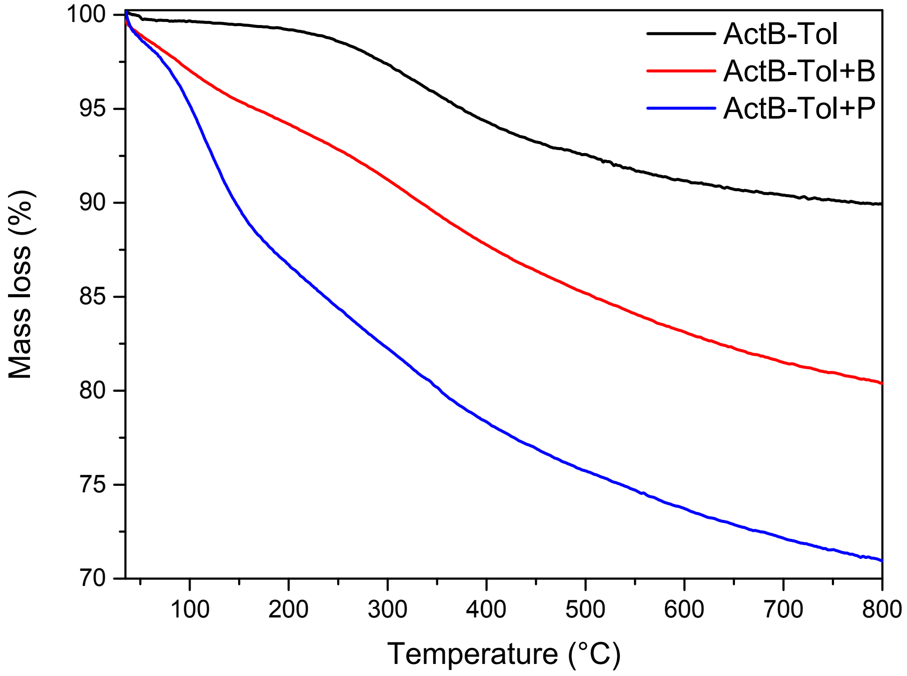

Figure S11. TGA records for sample **ActB-Tol** with and without adsorption of benzene (B) and pyridine (P).

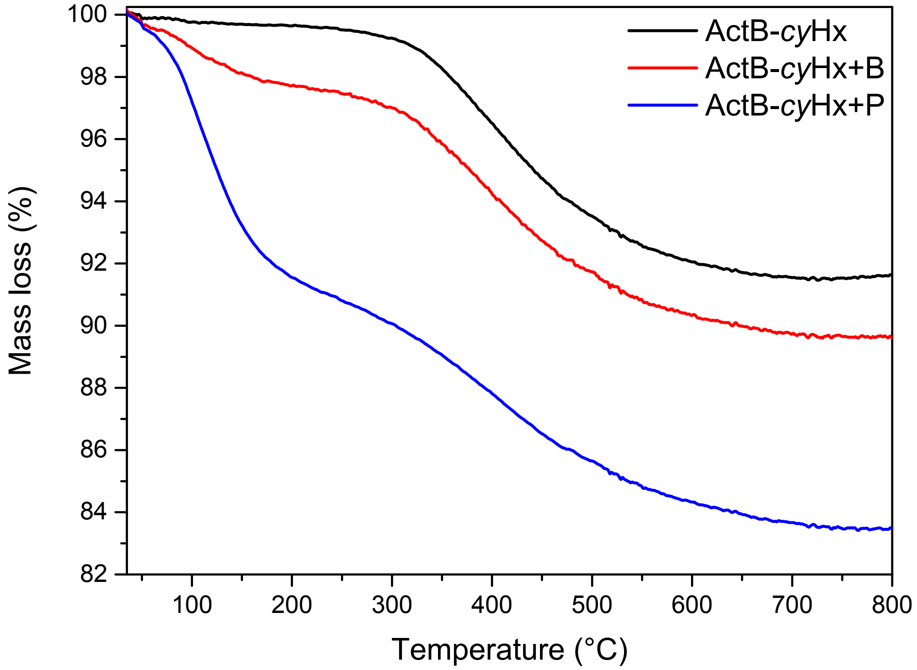

Figure S12. TGA records for sample **ActB-*cy*Hx** with and without adsorption benzene (B) and pyridine (P).

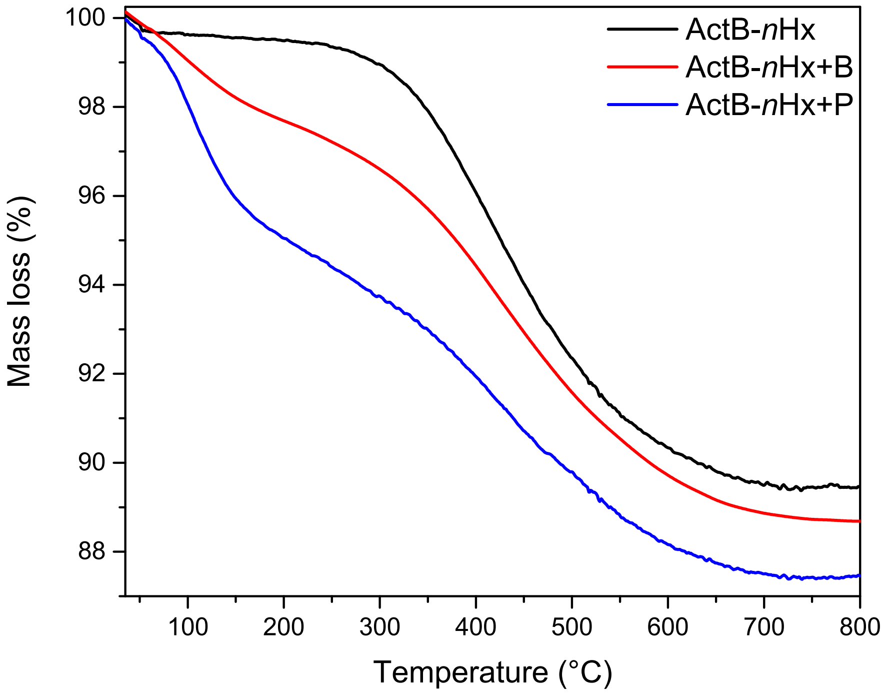

Figure S13. TGA records for sample **ActB-*n*Hx** with and without adsorption benzene (B) and pyridine (P).

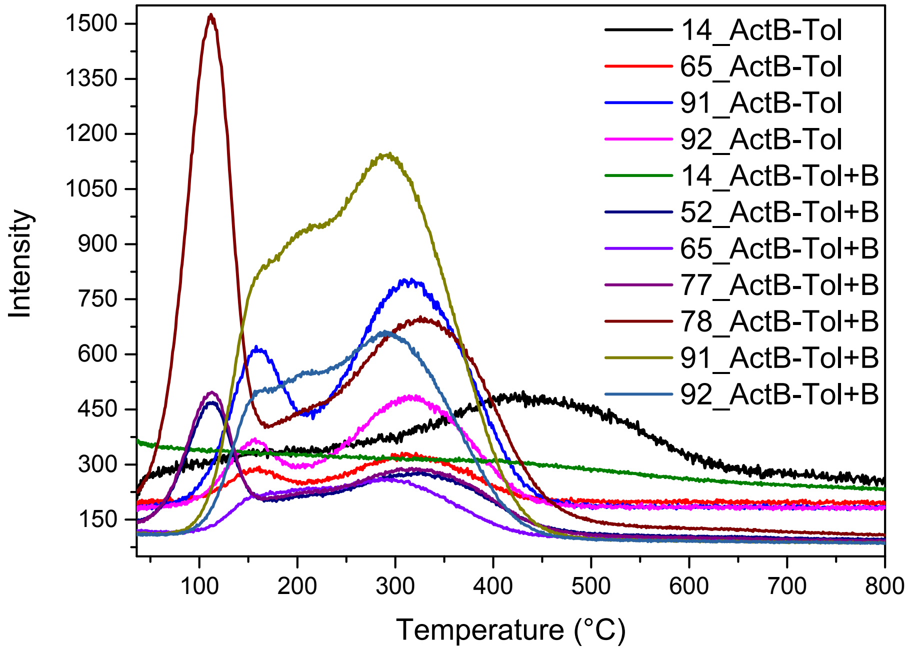

Figure S14. The records of selected values of m/z for **ActB-Tol** and **ActB-Tol** adsorbed with benzene.

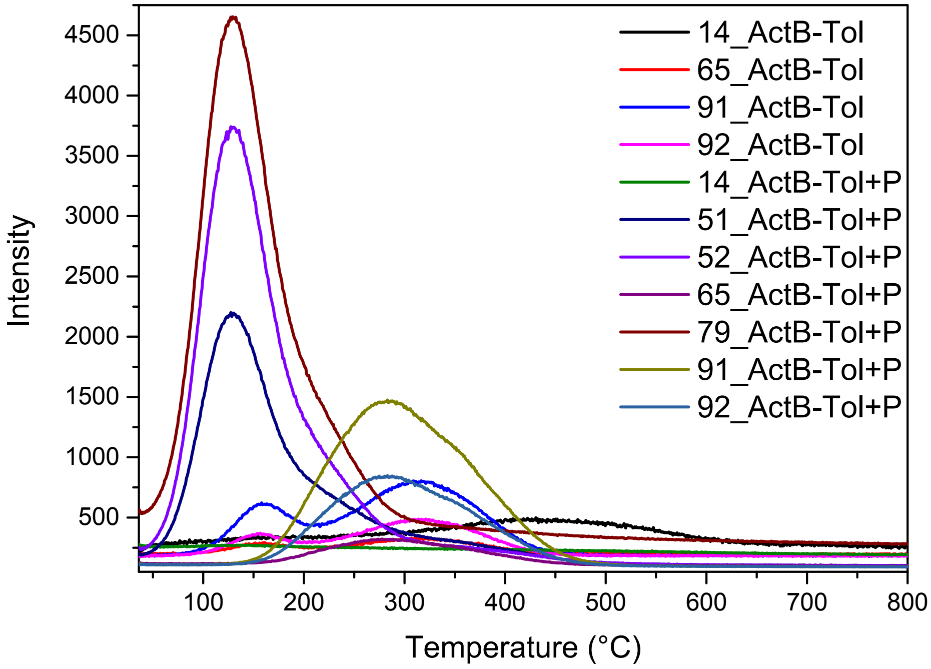

Figure S15. The records of selected values of m/z for **ActB-Tol** and **ActB-Tol** adsorbed with pyridine.

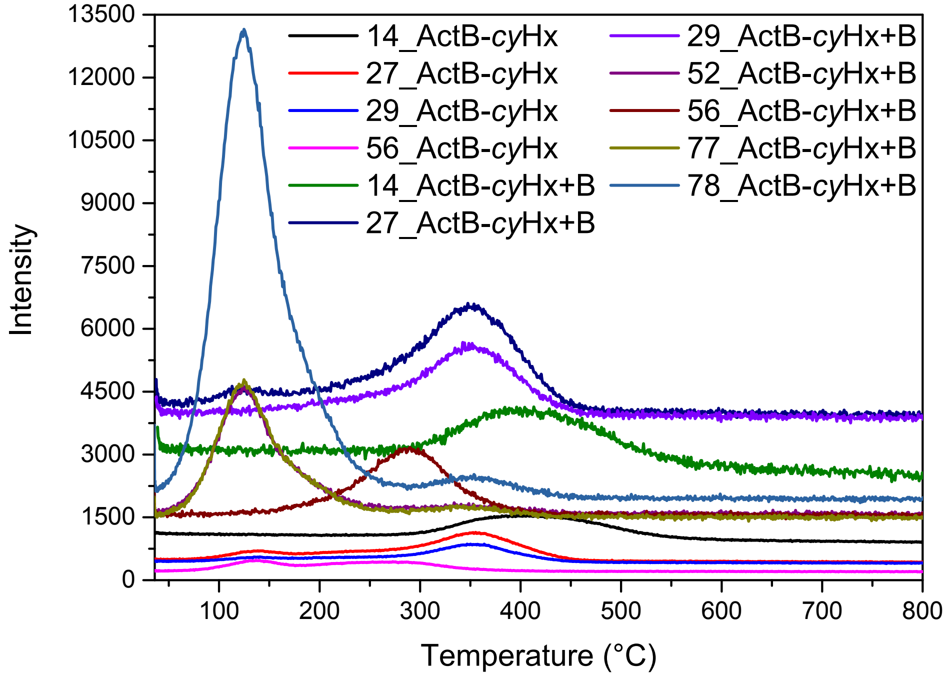

Figure S16. The records of selected values of m/z for **ActB-*cy*Hx** and **ActB-*cy*Hx** adsorbed with benzene.

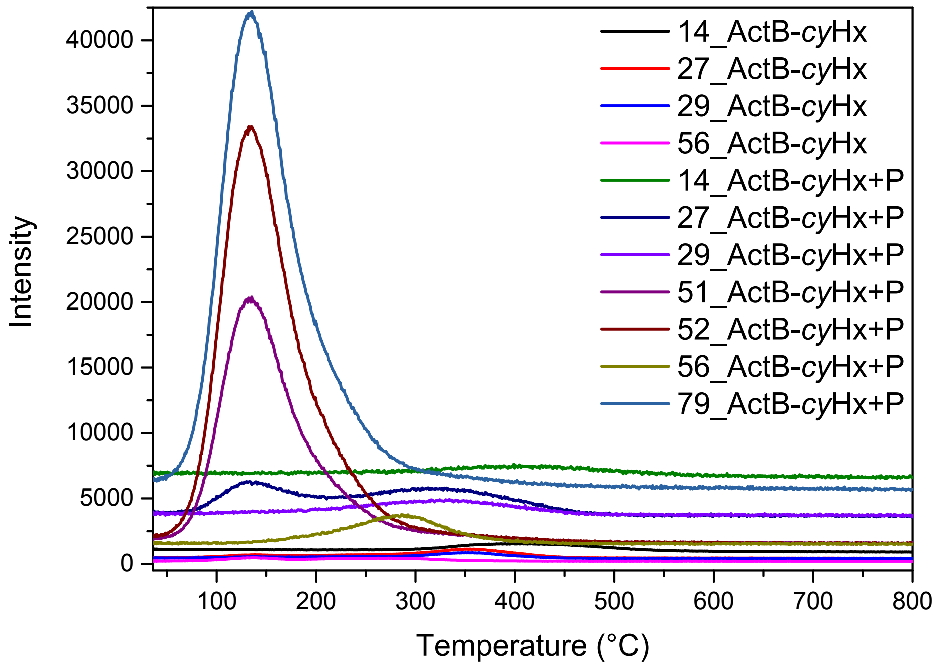

Figure S17. The records of selected values of m/z for **ActB-*cy*Hx** and **ActB-*cy*Hx** adsorbed with pyridine.

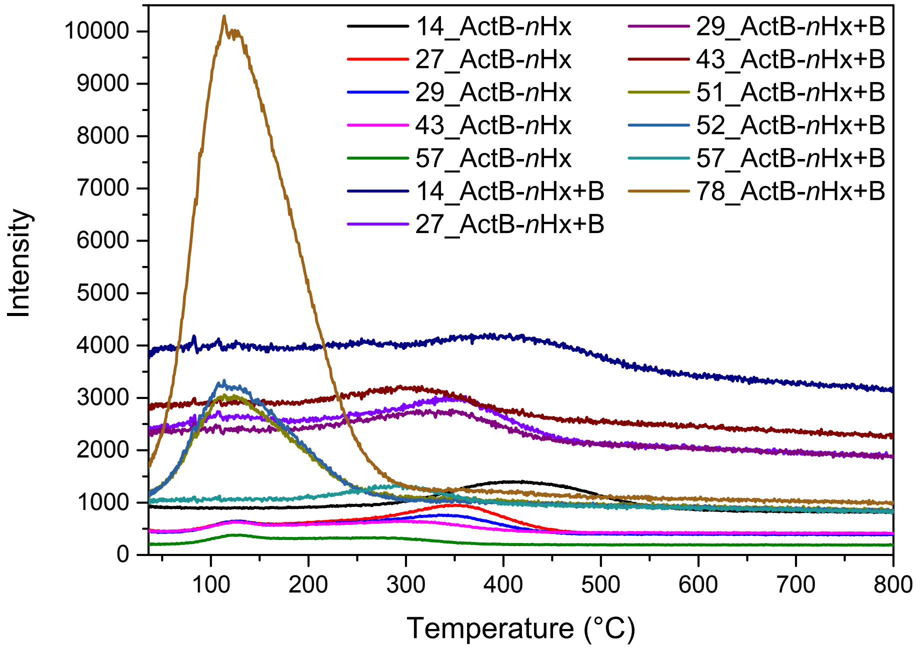

Figure S18. The records of selected values of m/z for **ActB-*n*Hx** and **ActB-*n*Hx** adsorbed with benzene.

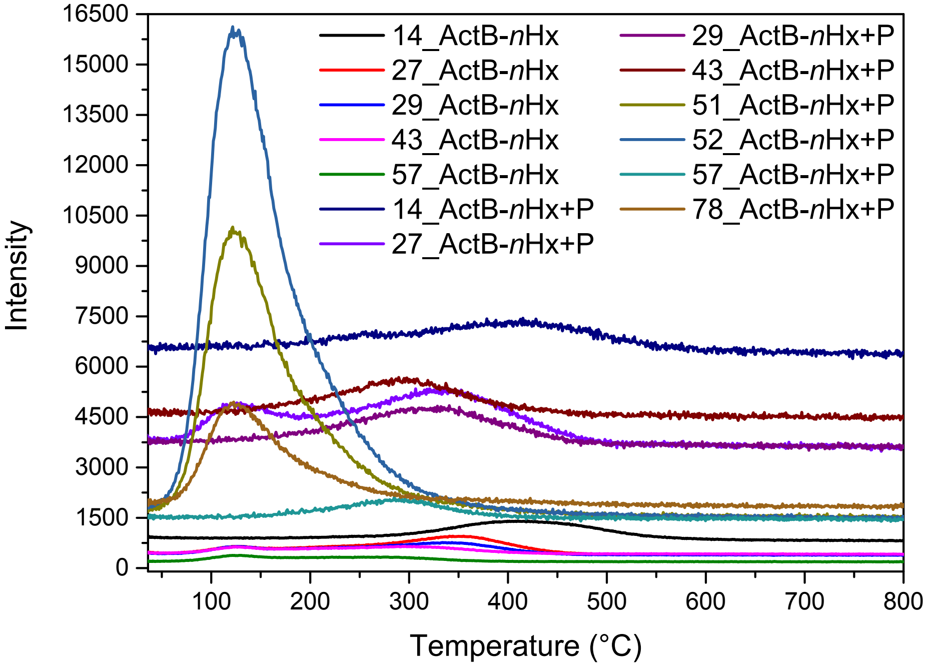

Figure S19. The records of selected values of m/z for **ActB-*n*Hx** and **ActB-*n*Hx** adsorbed with pyridine.

Hydrosilylation/deoxygenation catalysis

General Details. All operations were carried out under air- and moisture-free conditions (argon atmosphere) using Schlenk techniques or in an Inert Purelab He glovebox (under purified nitrogen atmosphere) unless noted otherwise. All solvents were appropriately dried (tetrahydrofuran and toluene were purified by a solvent purification system PureSolv MD 7, innovative Technology, Inc). Other liquid chemicals (silanes, acetophenone, benzaldehyde, cyclohexanone, 2-heptanone) were distilled under argon before use. Solid chemicals were used as received from commercial vendors. Flow reactions were carried out in a microfluidics-based flow reactor (XCube, ThalesNano) in a continuous flow mode. The catalysts were placed into 30 × 8 mm CatCart™ cartridges (approx. 0.2 g). Reaction mixtures were analysed by gas chromatography (Agilent technologies 7820A, BD-5 column), GC–MS (Thermo Focus DSQ, TR-5MS column), and NMR (a Varian Mercury 300, or a Bruker Avance Neo 500 MHz spectrometers). Spectra were referenced to the solvent residual signals ($CDCl_3$: ^1H: 7.26 ppm, ^{13}C: 77.16 ppm; Tol-d_8: ^1H: 2.08 ppm, ^{13}C: 20.43 ppm). Conversions and selectivities of the reactions were determined by GC. IR spectra were measured on a Nicolet Avatar spectrometer using an ATR accessory with an Si crystal, optionally using a gas-tight enclosure for the measurement under inert atmosphere.

General procedure for the hydrosilylation/deoxygenation reactions

ActB catalyst (20 mg) was weighed into a Schlenk tube with a magnetic stirrer in the glovebox, then connected to an Ar-vacuum line and the substrate (1 mmol) and appropriate solvent (2 mL) were added with stirring. The reaction was initiated by the addition of Et_3SiH (1.5 or 3.0 mmol), the reaction vessel was placed into a preheated oil bath and stirred for indicated time period, after which a sample was taken, centrifugated to remove the solid catalyst and immediately analysed by GC or other methods.

Reaction of benzophenone (1) and kinetic profile screening

A mixture of 20 mg **ActB** catalyst, benzophenone (**1**, 182 mg, 1.0 mmol), Et_3SiH (0.478 mL, 3.0 mmol) and 2 mL toluene was stirred at 60 °C for 22 h. The reaction mixture was analysed by GC,

GC-MS and NMR after removal of the catalyst by centrifugation. The crude product was isolated by column chromatography on silica gel using *n*-hexane as eluent, which afforded product **2** as a colourless oil (74 mg, 88% yield). NMR ($CDCl_3$) ^{1}H: δ 3.97 (s, 2 H, CH_2), 7.13-7.32 (m, 10 H, Ph); $^{13}C\{^1H\}$: δ 42.1 (CH_2), 126.2 (CH_p of Ph), 128.6, 129.1 (2x CH of Ph), 141.3 (C_{ipso} of Ph) ppm.

The kinetic profile screening was run in a double scale using 40 mg **ActB** catalyst, benzophenone (**1**, 364 mg, 2.0 mmol), Et_3SiH (0.956 mL, 6.0 mmol), and toluene (4 mL). Samples were taken after 5, 10, 15, 20, 25, 30, 60, 90, 120, 150, 210, 270 min and analysed by GC.

(**2**): MS, m/z (rel. int.): 168 (100, M^+); 152 (48); 139 (5); 115 (6); 91 (15); 82 (3); 65 (8); 51 (15); 39 (9); 32 (20); 28 (55)

(**3**): MS, m/z (rel. int.): 269 (35, M^+-Et); 167 (100); 152 (17); 135 (1), 115 (1); 74 (2), 59 (2); 45 (4)

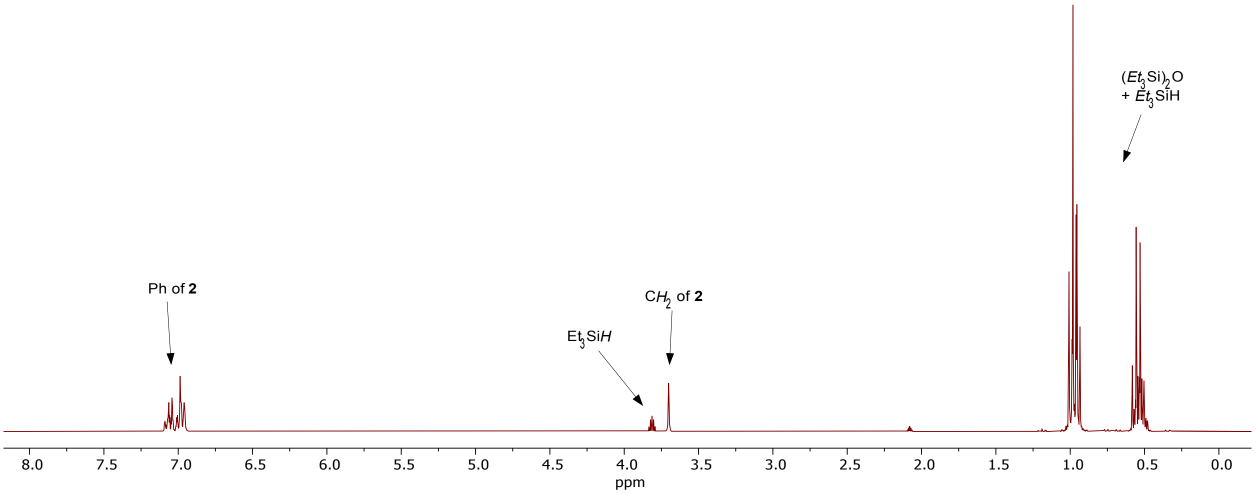

Figure S20. ^{1}H NMR spectra (300 MHz) of a reaction mixture of **1** performed in toluene-d_8 catalysed by **ActB-Tol** at 60 °C with 3 eq Et_3SiH at 22 h.

Reaction of benzophenone (1) catalysed by $B(C_6F_5)_3$

Tris(pentafluorophenyl)boron (BCF, 5 mg, 0.01 mmol) catalyst, benzophenone (**1**, 182 mg, 1.0 mmol), Et_3SiH (0.478 mL, 3.0 mmol) and 2 mL toluene was stirred at 60 °C for 2 h. The reaction mixture was analysed by GC and GC-MS.

Reaction of acetophenone (4)

A mixture of 20 mg **ActB**, acetophenone (**4**, 0.117 mL, 1.0 mmol), appropriate silane (1.5 or 3.0 mmol, respectively), and 2 mL of solvent was stirred at indicated temperature for defined period of time. The solution was centrifugated and the liquid samples were analysed by GC, GC-MS, and NMR.

(**5**): MS, m/z (rel. int.): 207 (49, M^+-Et); 115 (5); 103 (100); 77 (19); 75 (67); 59 (18); 47 (27); 28(18)

(**6**): MS, m/z (rel. int.): 104 (100, M^+); 78 (60); 77(32); 63(18); 51(57); 39(13); 32(10); 28(21)

(**7**): MS, m/z (rel. int.): 106 (18, M^+); 91 (100); 77 (7); 65 (12); 51 (11); 39 (6); 28 (5)

(**8**): MS, m/z (rel. int.): (M^+ not visible); 121 (49); 105 (100); 91 (8); 79 (26); 77 (42); 63 (3); 51 (12); 43 (6); 32 (8); 28 (22)

Table S9. *Product distribution in PhAc (**4**) silylation/deoxygenation reaction by Et_3SiH, using different **ActB** catalysts, at 60 °C, toluene solvent (Scheme 2). Yield (%) determined by GC.*

cat	Et_3SiH	t (h)/T (°C)	4	5	6	7	8
**ActB-Tol**	1.5 eq	22 / 60	22	0	38	39	0
**ActB-Tol**	1.5 eq	44 / 60	10	0	48	39	2
**ActB-Tol**	3.0 eq	22 / 60	22	0	35	31	6
**ActB-Tol**	3.0 eq	44 / 60	5	0	44	38	7
**ActB-*cy*Hx**	1.5 eq	22 / 60	8	67	15	7	0
**ActB-*cy*Hx**	1.5 eq	44 / 60	6	54	26	9	2
**ActB-*cy*Hx**	3.0 eq	22 / 60	4	69	13	8	3
**ActB-*cy*Hx**	3.0 eq	44 / 60	2	54	22	11	6
**ActB-*n*Hx**	1.5 eq	22 / 60	80	19	1	0	0
**ActB-*n*Hx**	1.5 eq	44 / 60	21	78	1	0	0
**ActB-*n*Hx**	3.0 eq	22 / 60	34	62	2	2	0
**ActB-*n*Hx**	3.0 eq	44 / 60	22	70	4	2	0

Reaction of acetophenone (4) catalysed by $B(C_6F_5)_3$

Tris(pentafluorophenyl)boron (BCF, 5 mg, 0.01 mmol) catalyst, acetophenone (**4**, 0.117 mL, 1.0 mmol), Et_3SiH (0.478 mL, 3.0 mmol) and 2 mL toluene was stirred at 60 °C for 2 h and the reaction mixture was analysed by GC.

Table S10. *Product distribution in PhAc (**4**) silylation/deoxygenation reaction by Et_3SiH, using $B(C_6F_5)_3$ catalyst (BCF). Yield (%) determined by GC.*

Cat	Et_3SiH	t (h)/T (°C)	4	5	6	7	8
BCF	3.0 eq	2 / 60	0	25	0	70	1

Table S11. *Product distribution in **ActB-Tol** catalysed PhAc (**4**) silylation/deoxygenation reaction using different silanes at RT and 120 °C, 1.5 eq Et_3SiH, toluene solvent. Yield (%) determined by GC.*

Silane	t (h)/T (°C)	4	5	6	7	other (sum)
$HSiEt_3$	22 / RT	39	0	24	28	8
$HSiEt_3$	22 / 120	0	0	27	60	12
$HSi(OEt)_3$	22 / RT	92	0	0	0	7
$HSi(OEt)_3$	22 / 120	87	0	2	3	7
$HSiPh_3$	22 / RT	97	0	1	1	0
$HSiPh_3$	22 / 120	91	0	2	6	0
H_2SiEt_2	22 / RT	41	0	13	17	28
H_2SiEt_2	22 / 120	0	0	6	57	36
H_2SiPh_2	22 / RT	58	0	1	6	33
H_2SiPh_2	22 / 120	2	0	1	31	65

Table S12. *Solvent screening for the PhAc (**4**) silylation/deoxygenation reaction performed with **ActB-Tol** catalyst, 1.5 eq Et_3SiH, 22 h, 60 °C. Conversion of **4** is shown as determined by GC.*

	toluene	THF	dioxane	MeCN	$PhCF_3$	PhCl	n-hexane	mesitylene	CH_3NO_2
PhAc conversion	78	63	55	20	32	27	25	37	31

Table S13. *Temperature optimization for the PhAc (**4**) silylation/deoxygenation reaction performed with **ActB-Tol** catalyst, 3.0 eq Et_3SiH, 22 h, toluene (Scheme 2). Yield (%) determined by GC.*

t (h)/T (°C)	4	5	6	7	8
22 / 60	22	0	35	31	6
22 / 80	9	0	29	55	5
22 / 100	0	0	12	81	5
22 / 120	0	0	27	60	8

Table S14. *Effect of air-exposure (**ActB-Tol** material left for 1 h in air) on the PhAc (**4**) silylation/deoxygenation reaction. **ActB-Tol** catalyst, 1.5 eq Et_3SiH, 22 h, 60 °C, toluene.*

cat	4	5	6	7	other (sum)
**ActB-Tol**	22	0	38	39	0
**ActB-Tol** (air-exposed)	21	0	31	33	5

Table S15. *Product distribution in PhAc (**4**) silylation/deoxygenation reaction by Et_3SiH, using different **ActB** catalysts, THF solvent (Scheme 2). Yield (%) determined by GC.*

cat	Et_3SiH	t (h)/T (°C)	4	5	6	7	8
**ActB-Tol**	1.5 eq	22 / 60	37	0	44	12	0
**ActB-Tol**	1.5 eq	44 / 60	28	0	50	15	0
**ActB-Tol**	3.0 eq	22 / 60	21	0	51	17	5
**ActB-Tol**	3.0 eq	44 / 60	11	0	58	20	5
**ActB-*cy*Hx**	1.5 eq	22 / 60	9	75	14	2	0
**ActB-*cy*Hx**	1.5 eq	44 / 60	5	51	34	8	2
**ActB-*cy*Hx**	3.0 eq	22 / 60	13	1	37	11	38
**ActB-*cy*Hx**	3.0 eq	44 / 60	8	0	41	13	38
**ActB-*cy*Hx**	1.5 eq	3 / RT	37	59	2	1	1
**ActB-*cy*Hx**	1.5 eq	6 / RT	23	71	4	1	1
**ActB-*cy*Hx**	1.5 eq	22 / RT	0	90	6	2	2
**ActB-*cy*Hx**	3.0 eq	3 / RT	15	81	2	1	1
**ActB-*cy*Hx**	3.0 eq	6/ RT	8	85	4	2	1
**ActB-*cy*Hx**	3.0 eq	22 / RT	0	91	5	3	1

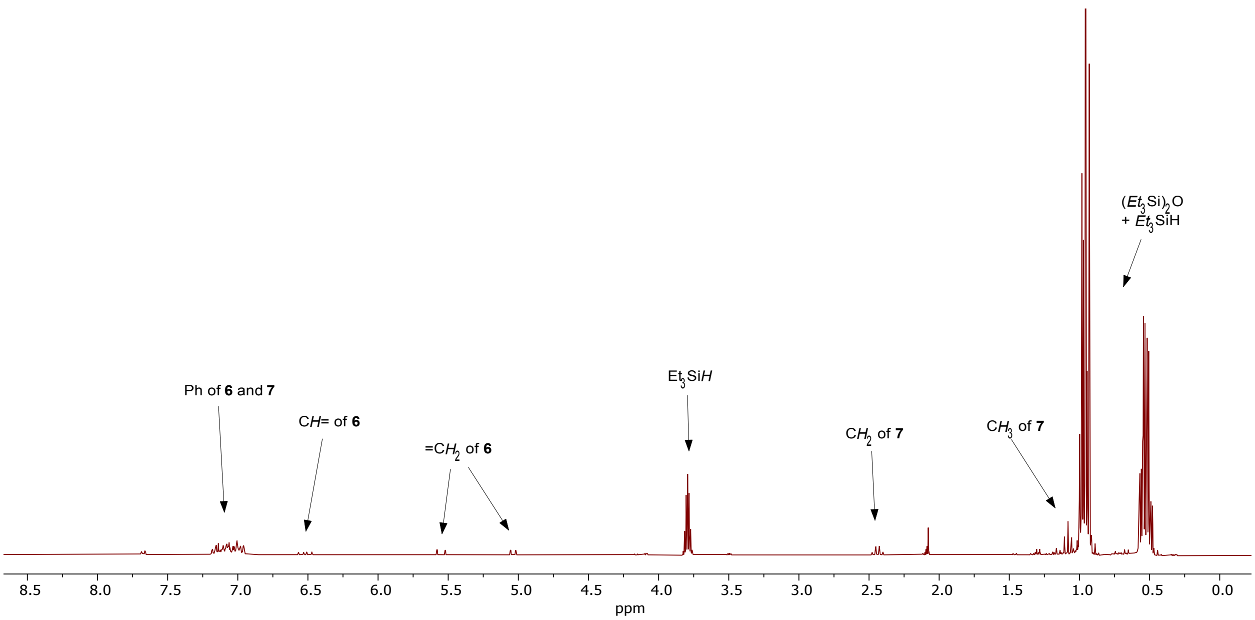

Figure S21. ^{1}H NMR spectra (300 MHz) of a reaction mixture of **4** performed in toluene-d_8 catalysed by **ActB-Tol** at 60 °C with 3 eq Et_3SiH at 22 h.

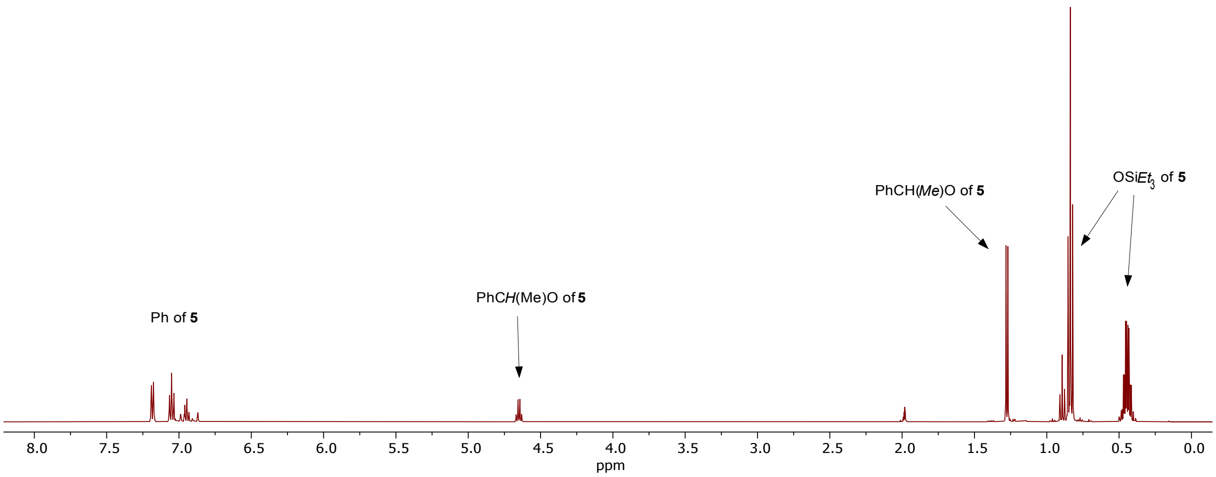

Figure S22. ^{1}H NMR spectra (500 MHz) of a reaction mixture of **4** catalysed by **ActB-cyHx** at 60 °C with 3 eq Et_3SiH at 22 h, volatiles removed, residue dissolved in toluene-d_8.

Reaction of 5 with and without Et_3SiH catalysed by ActB-Tol

The silylether **5** (123 mg, 0.5 mmol), prepared independently by silylation of alcohol **9**, was added to a mixture of Et_3SiH (0.239 mL, 1.5 mmol), **ActB-Tol** (10 mg) and 1 mL toluene. The mixture was stirred at 60 °C for 22 h and then analysed by GC and NMR. In another experiment, the above reaction was repeated without Et_3SiH.

Reaction of 9 with and without Et_3SiH catalysed by ActB-Tol

Rac-1-phenylethanol **9** (61 mg, 0.5 mmol) was added to a mixture of Et_3SiH (0.239 mL, 1.5 mmol), **ActB-Tol** (10 mg) and 1 mL toluene. The mixture was stirred at 60 °C for 22 h and then analysed by GC and NMR. In another experiment, the above reaction was repeated without Et_3SiH.

Reaction of styrene (6) with Et_3SiH catalysed by ActB-Tol

Styrene (**6**, 115 µL, 1.0 mmol) was added to a mixture of Et_3SiH (0.478 mL, 3.0 mmol), **ActB-Tol** (20 mg) and 2 mL toluene. The mixture was stirred at 60 °C for 22 h and then analysed by GC and NMR. No conversion was observed.

Blank experiments with different boron-based materials

Reactions of **1** with Et_3SiH were performed in the same way as that described above for 22 h in toluene using 20 mg of a) *nido*-$B_{10}H_{14}$, b) a thermolyzed sample of *nido*-$B_{10}H_{14}$ (performed without solvent for 24 h at 250 °C, yielding a dark brown powder, insoluble in toluene), c) 5-Ph-$B_{10}H_{13}$, d) $(HNEt_3)_2(B_{12}H_{12})^{2-}$. In all cases, no catalytic activity (i.e., no conversion of the substrate) was observed.

Reproducibility of catalysis using different ActB-Tol batches

The reactions of **1** and **4** with Et_3SiH were performed in the same way as that described above, using two different batches of **ActB-Tol** catalyst. Benzophenone (**1**) was fully converted to the deoxygenation product **2** by both catalysts. The results from the reactions of **4** at different silane stoichiometries and conditions are summarized in Table S16.

Table S16. *Product distribution in PhAc (**4**) silylation/deoxygenation reaction by Et_3SiH, using different batches of **ActB-Tol** catalyst, toluene solvent (Scheme 2). Yield (%) determined by GC.*

cat	Et_3SiH	t (h)/T (°C)	4	5	6	7	8
**ActB-Tol (B1)**	1.5 eq	22 / 60	22	0	38	39	0
**ActB-Tol (B1)**	1.5 eq	44 / 60	10	0	48	39	2
**ActB-Tol (B1)**	3.0 eq	22 / 60	22	0	35	31	6
**ActB-Tol (B1)**	3.0 eq	44 / 60	5	0	44	38	7
**ActB-Tol (B2)**	1.5 eq	22 / 60	24	0	36	37	1
**ActB-Tol (B2)**	1.5 eq	44 / 60	10	0	45	43	2
**ActB-Tol (B2)**	3.0 eq	22 / 60	20	0	38	34	3
**ActB-Tol (B2)**	3.0 eq	44 / 60	8	0	42	38	6

Reaction of benzaldehyde (10)

A mixture of 20 mg **ActB-Tol** or **ActB-*cy*Hx**, benzaldehyde (**10**, 0.102 mL, 1.0 mmol) Et_3SiH (0.478 mL, 3.0 mmol) and 2 mL toluene was stirred in an inert atmosphere at 60 °C or 100 °C and for 22 h or 44 h, respectively. The reaction mixtures were analysed by GC, GC-MS, and NMR.

(**11**): MS, m/z (rel. int.): (M^+ not visible); 107 (11); 91 (100); 77 (9); 65 (41); 51 (38); 39 (18); 28 (2)

(**12**): MS, m/z (rel. int.): 193 (100, M^+-Et); 163 (49); 135 (33); 91 (48); 59 (13); 49 (11); 28 (9)

Table S17. *Hydrosilylation of benzaldehyde (**10**) by Et_3SiH catalysed by **ActB** materials.*

Catalyst	t (h)/T (°C)	10	11	12	other (sum)
**ActB-Tol**	22 / 60	0	72	11	17
**ActB-Tol**	44 / 60	0	76	4	20
**ActB-*cy*Hx**	22 / 60	0	16	77	7
**ActB-*cy*Hx**	44 / 60	0	16	76	8

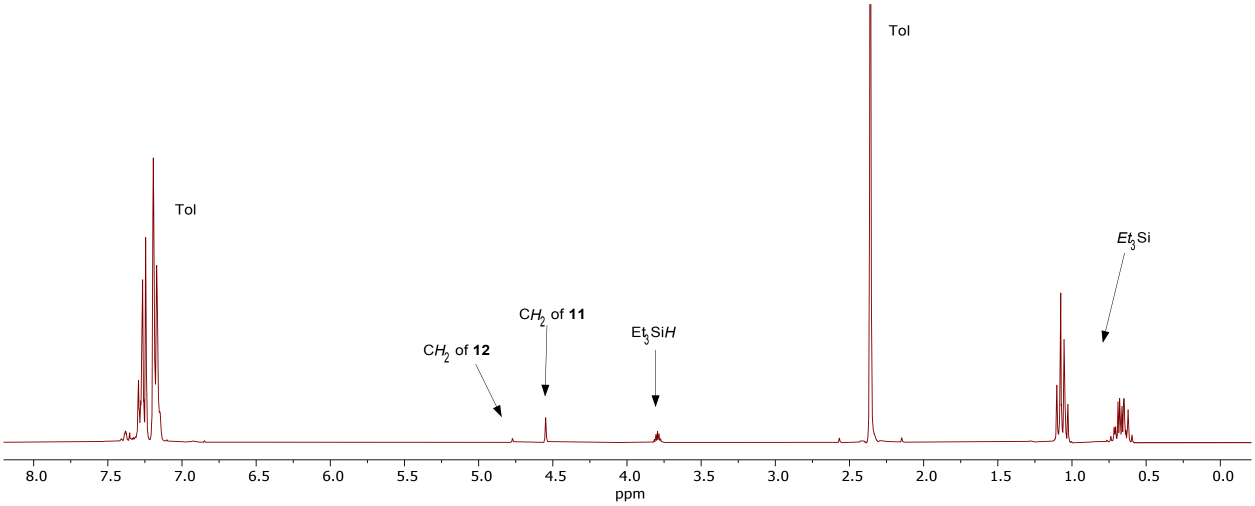

Figure S23. ^{1}H NMR spectra (300 MHz) of a crude reaction mixture of **10** in toluene catalysed by **ActB-Tol** at 60 °C with 3 eq Et_3SiH at 22 h, 0.1 mL of the reaction mixture + 0.5 mL $CDCl_3$.

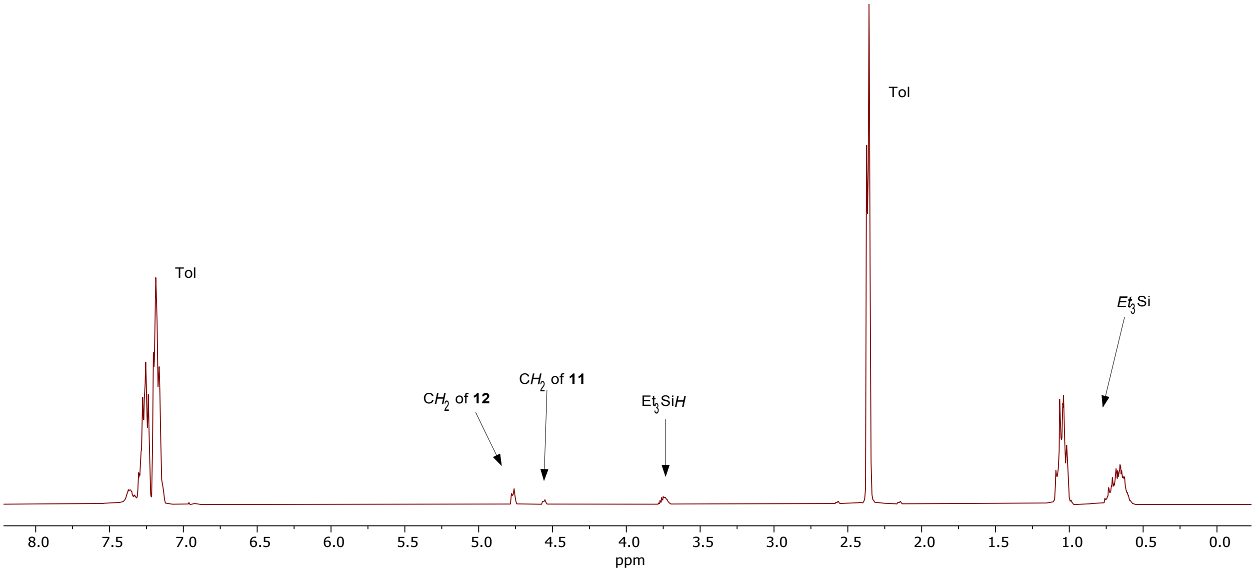

Figure S24. ^{1}H NMR spectra (300 MHz) of a crude reaction mixture of **10** in toluene catalysed by **ActB-*cyHx*** at 60 °C with 3 eq Et_3SiH at 22 h, 0.1 mL of the reaction mixture + 0.5 mL $CDCl_3$.

Reaction of *trans*-chalcone (13)

A mixture of 20 mg **ActB-Tol** or **ActB-*cy*Hx**, *trans*-chalcone (**13**, 208 mg, 1.0 mmol), Et_3SiH (0.239 mL or 0.478 mL, 1.5 or 3.0 mmol, respectively) and 2 mL toluene was stirred at 60 °C for 22 h. Reaction mixtures were analysed by GC, GC-MS, and NMR.

(**14**): MS, m/z (rel. int.): 324 (37, M^+); 295 (5); 219 (77); 193 (65); 163 (10); 147 (8); 135 (41); 115 (74); 103 (32); 91 (46); 77 (27); 65 (11); 59 (100); 45 (7); 28 (10)

(**15**): MS, m/z (rel. int.): 210 (100, M^+); 105 (100); 91 (20); 77 (89); 65 (16); 51 (32); 39 (8); 28 (12)

Table S18. *Hydrosilylation of trans-chalcone (**13**) by Et_3SiH catalysed by **ActB** materials.*

Catalyst	EQ	t (h)/T (°C)	13	14	15	other
**ActB-Tol**	1.5	22 / 60	0	48	37	15
**ActB-Tol**	3.0	22 / 60	0	87	10	3
**ActB-*cy*Hx**	3.0	22 / 60	0	98	1	1

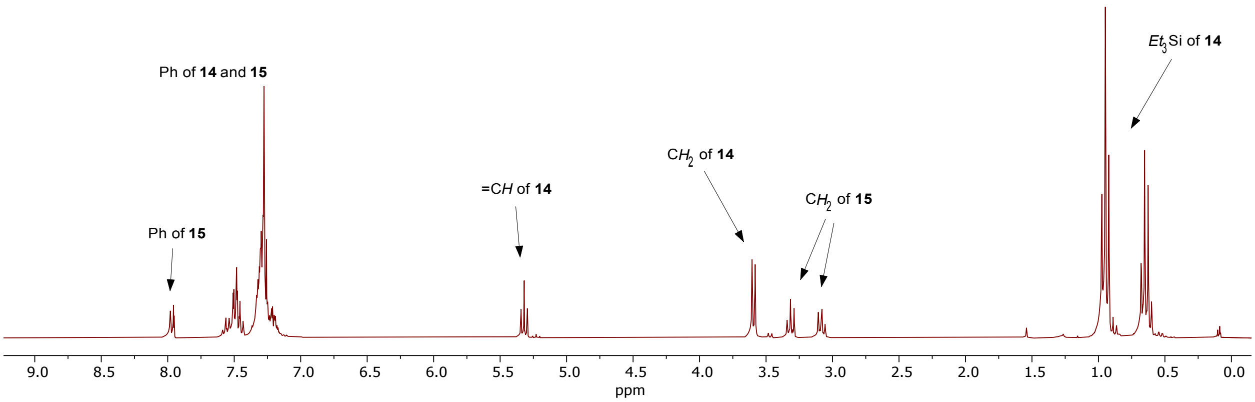

Figure S25. ^1H NMR spectra (300 MHz) of a reaction mixture of **13** catalysed by **ActB-Tol** at 60 °C, volatiles removed and the residue dissolved in $CDCl_3$.

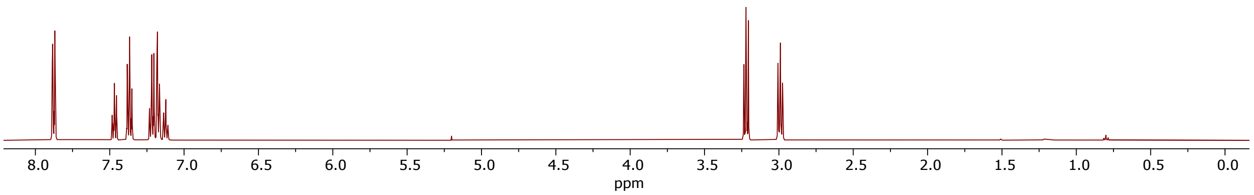

Figure S26. ^{1}H NMR spectra (500 MHz) of compound **15** in $CDCl_3$, which eventually crystallized from NMR sample of crude reaction mixture of **13** catalysed by **ActB-Tol**.

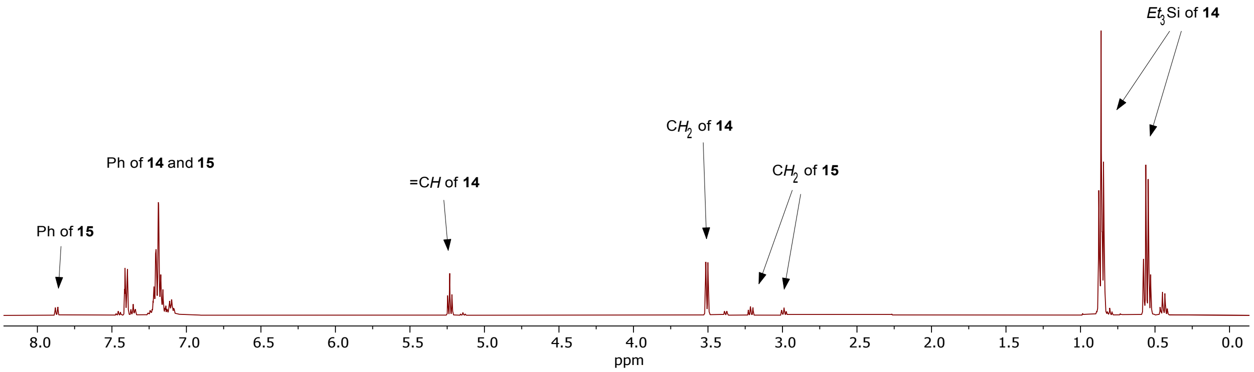

Figure S27. ^{1}H NMR spectra (500 MHz) of a reaction mixture of **13** catalysed by **ActB-cyHx** at 60 °C, volatiles removed and the residue dissolved in $CDCl_3$.

Reaction of benzil (16)

A mixture of 20 mg **ActB-Tol** or **ActB-*cy*Hx**, benzil (**16**, 0.210 mg, 1.0 mmol), Et_3SiH (0.956 mL, 6.0 mmol), and 2 mL toluene was stirred at 60 °C or 110 °C for 22 h. Reaction mixtures were analysed by GC, GC-MS, and NMR.

(**17**): MS, m/z (rel. int.): 413 (1, M^+-Et); 221 (100); 193 (4); 178 (5); 163 (9); 149 (4); 135 (5); 114 (6); 86 (7); 59 (16); 28 (7)

(**18**): MS, m/z (rel. int.): 283 (73, M^+-Et); 178 (17); 167 (69); 150 (32); 135 (24); 117 (100); 115 (35); 103 (57); 87 (13); 75 (6); 59 (76); 47 (33); 28 (14)

(**19**): MS, m/z (rel. int.): (M^+ not visible); 181 (100); 165 (85); 152 (10); 141 (4); 115 (2); 91 (19); 77 (1); 65 (18); 51 (6); 38 (7); 28 (17)

(**20**): MS, m/z (rel. int.): 180 (100, M^+); 178 (72); 165 (46); 152 (16); 139 (5); 115 (5); 102 (10); 89 (6); 63 (20); 51 (27); 39 (21); 28 (5)

(**21**): MS, m/z (rel. int.): 182 (43, M^+); 104 (4); 91 (100); 65 (75); 51 (24); 32 (38); 28 (3)

Table S19. *Hydrosilylation/deoxygenation of benzil by Et_3SiH catalysed by **ActB** materials.*

Catalyst	t (h)/T (°C)	16	17	18	19	20	21
**ActB-Tol**	22 / 60	0	0	68	15	10	5
**ActB-Tol**	22 / 110	0	0	29	15	42	1
**ActB-*cy*Hx**	22 / 60	1	59	39	0	1	0

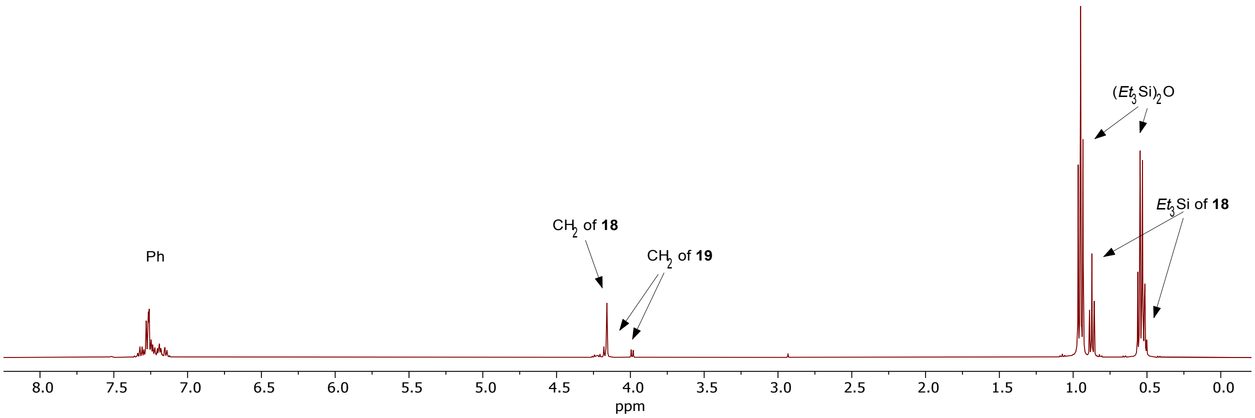

Figure S28. ^{1}H NMR spectra (300 MHz) of a reaction mixture of **16** catalysed by **ActB-Tol** at 60 °C, volatiles removed and the residue dissolved in $CDCl_3$.

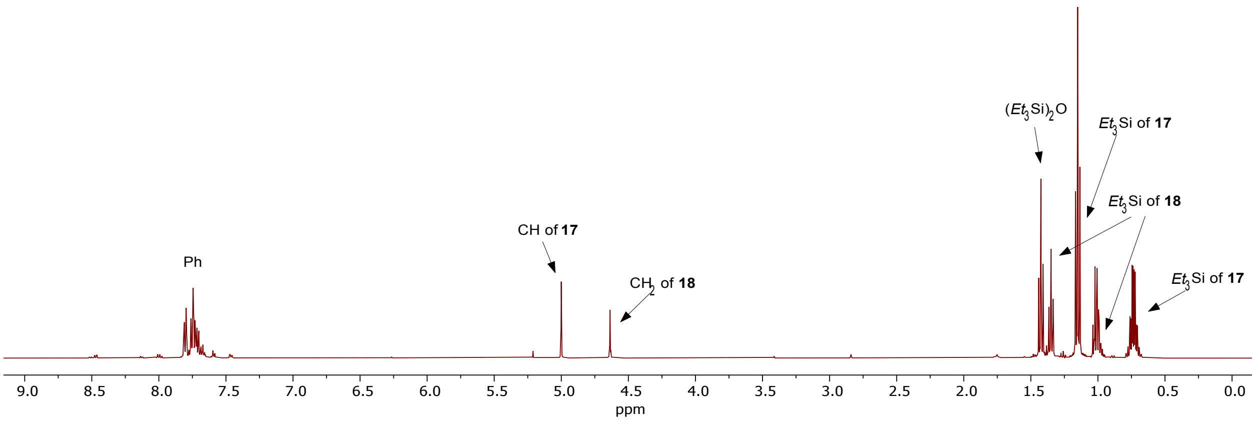

Figure S29. ^{1}H NMR spectra (300 MHz) of a reaction mixture of **16** catalysed by **ActB-*cy*Hx** at 60 °C, volatiles removed and the residue dissolved in $CDCl_3$.

Reaction of cyclohexanone (22)

A mixture of 20 mg **ActB-Tol** or **ActB-*cy*Hx**, cyclohexanone (**22**, 0.103 mL, 1.0 mmol), Et_3SiH (0.478 mL, 3.0 mmol), and 2 mL toluene was stirred at 60 °C or 80 °C for 22 h. Reaction mixtures were analysed by GC, GC-MS, and NMR.

(**23**): MS, m/z (rel. int.): 185 (100, M^+-Et); 103 (60); 87 (20); 75 (48); 55 (60); 41 (56); 29 (26)

(**24**): MS, m/z (rel. int.): 182 (12, M^+); 100 (38); 82 (83); 67 (16); 55 (100); 41 (75); 28 (15)

(**25**): MS, m/z (rel. int.): 84 (79, M^+); 69 (37); 56 (100); 41 (45); 39 (25); 27 (11)

(**26**): MS, m/z (rel. int.): 82 (23, M^+); 67 (8); 56 (59); 41 (23); 32 (59); 28 (100)

Table S20. *Hydrosilylation/deoxygenation of cyclohexanone by Et_3SiH catalysed by **ActB** materials.*

Catalyst	t (h)/T (°C)	22	23	24	25	26
**ActB-Tol**	22 / 60	7	19	42	12	19
**ActB-Tol**	22 / 80	0	2	29	53	8
**ActB-*cy*Hx**	22 / 60	1	81	13	1	3

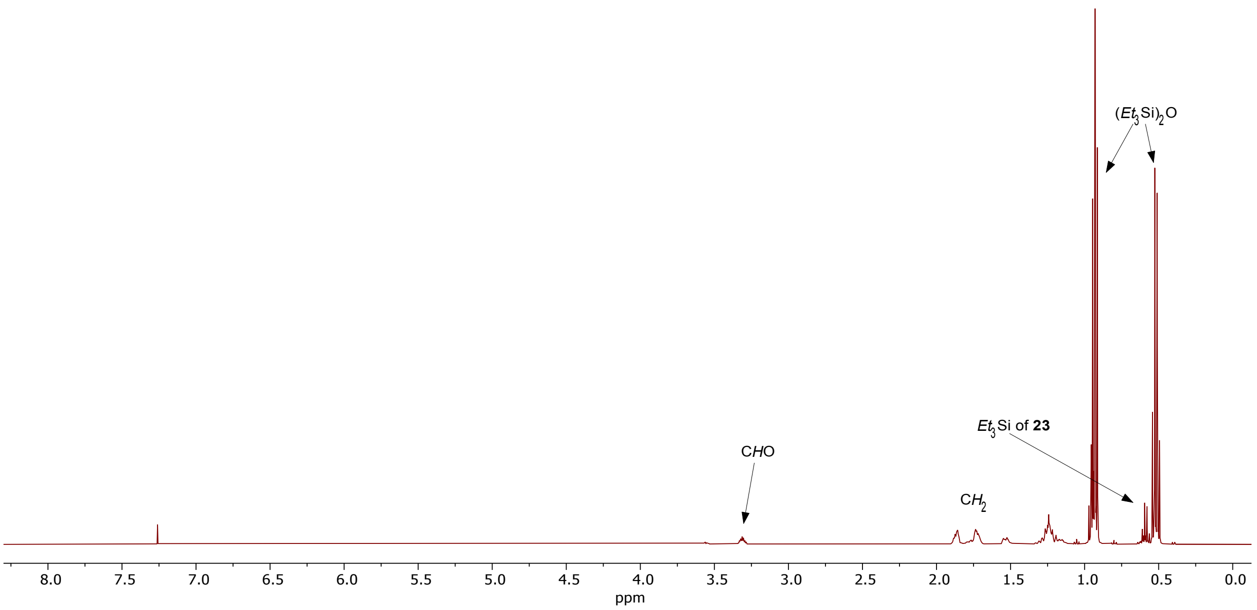

Figure S30. ^{1}H NMR spectra (300 MHz) of a reaction mixture of **22** catalysed by **ActB-Tol** at 60 °C, volatiles removed and the residue dissolved in $CDCl_3$.

Reaction of 2-heptanone (27)

A mixture of 20 mg **ActB-Tol** or **ActB-*cy*Hx**, 2-heptanone (**27**, 0.143 mL), Et_3SiH (0.478 mL, 3.0 mmol), and 2 mL toluene was stirred at 60 °C or 100 °C for 22 h. Reaction mixtures were analysed by GC, GC-MS, and NMR.

(**28**): MS, m/z (rel. int.): 201 (100, M^+-Et); 159 (12); 143 (9); 131 (9); 115 (15); 103 (68); 87 (5); 75 (17); 59 (11); 47 (12); 29 (7)

(**29**): MS, m/z (rel. int.): 100 (9, M^+); 85 (3); 71 (41); 43 (100); 41 (57); 39 (11); 29 (42); 27 (40)

(**30**): MS, m/z (rel. int.): 98 (3, M^+); 83 (4); 54 (18); 45 (86); 32 (41); 28 (100)

(**31**): MS, m/z (rel. int.): (M^+ not visible); 143 (20); 125 (9); 99 (37); 83 (2); 57 (100); 43 (22); 29 (18)

Table S21. *Hydrosilylation/deoxygenation of 2-heptanone by Et_3SiH catalysed by* ***ActB*** *materials.*

cat	t (h)/T (°C)	27	28	29	30	31
**ActB-Tol**	22 / 60	4	70	6	6	13
**ActB-Tol**	22 / 100	0	31	24	25	13
**ActB-*cy*Hx**	22 / 60	1	95	0.5	0.5	3

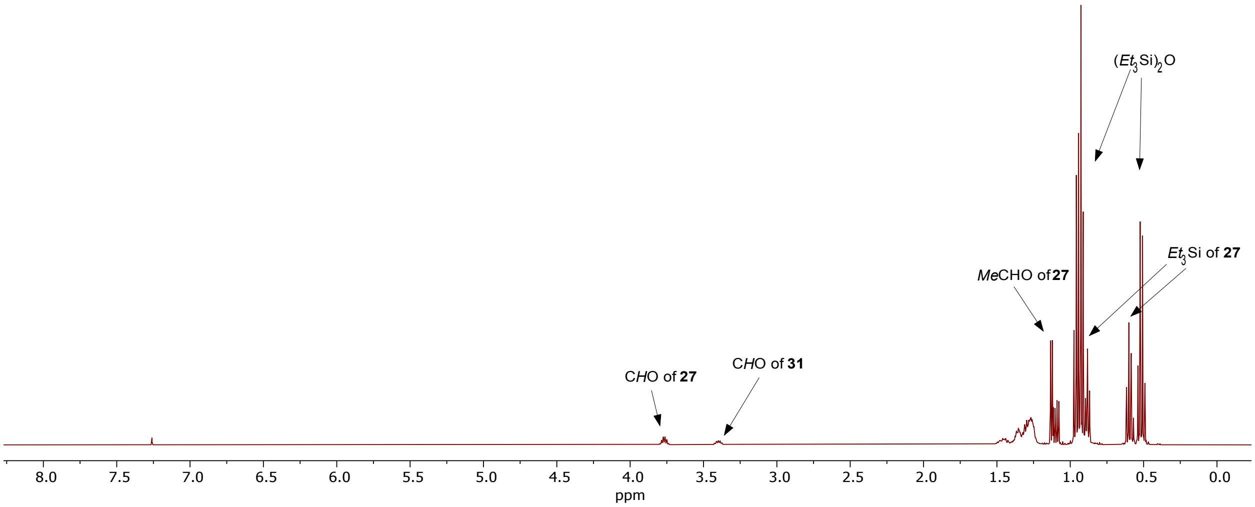

Figure S31. ^{1}H NMR spectra (500 MHz) of a reaction mixture of **27** catalysed by **ActB-Tol** at 60 °C, volatiles removed and the residue dissolved in $CDCl_3$.

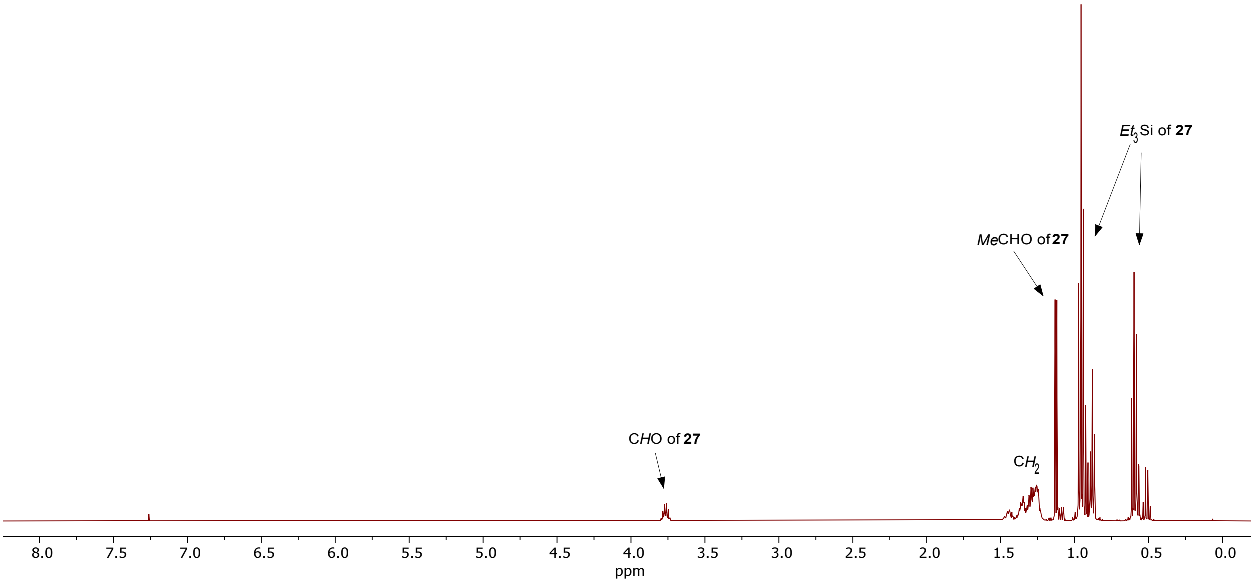

Figure S32. ^{1}H NMR spectra (500 MHz) of a reaction mixture of **27** catalysed by **ActB-cyHx** at 60 °C, volatiles removed and the residue dissolved in $CDCl_3$.

General procedure for the hydrosilylation/deoxygenation reaction in the flow reactor

The reactions were carried out in the microfluidics-based flow reactor (XCubeTM) in continuous flow mode. Studied **ActB** catalysts were mixed with a TMS-protected silica-gel (1:3.1 m/m ratio). This catalyst composition was placed into a 30 × 8 mm CatCartTM cartridge (approx. 0.2 g was used). The catalyst bed was washed continuously with the toluene solutions of substrate (1 mmol), Et_3SiH (3.0 mmol) with 0.1 mL/min flow rate (residence time on the catalyst bed: 3 min). The temperature was set at 100 °C. Conversions and selectivities of the reactions were determined by GC on samples taken at indicated time periods.

Detailed discussion of the hydrosilylation/deoxygenation substrate scope

ActB-Tol and **ActB-*cy*Hx** catalysts were tested in the hydrosilylation/deoxygenation reaction of various carbonyl-containing substrates as shown below. Benzaldehyde (**10** in Scheme S1) was fully converted using **ActB-Tol** predominantly to the ether **11** with a smaller amount of the silylated product **12**, while **ActB-*cy*Hx** gave approximately the opposite selectivity with a preference for the silyl ether **12**. Prolonged reaction time with **ActB-Tol** even increased the ratio of products in favour of **11**.

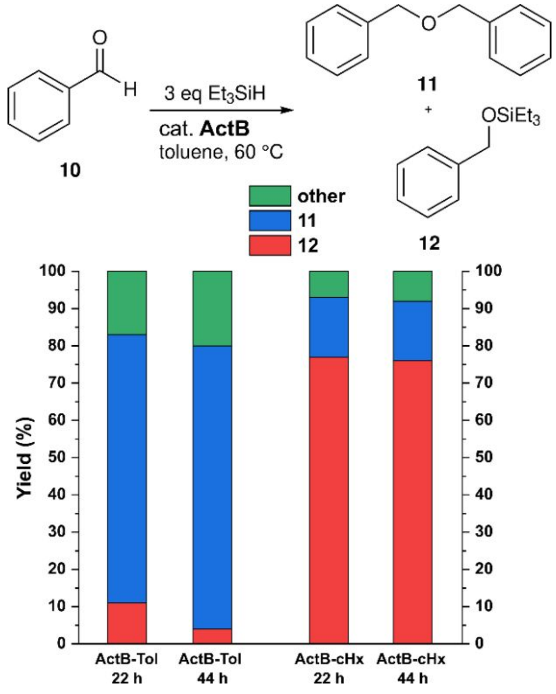

Scheme S1. Hydrosilylation of benzaldehyde (10) catalysed by **ActB** materials.

Trans-chalcone (**13**) was employed as a substrate having a conjugated enone moiety (Scheme S2). Indeed, a conjugated 1,4-addition of hydrosilane that afforded product **14** was observed as a dominant transformation in this case, accompanied by the ketone **15**, the formation of which was supressed by a larger excess of silane. **ActB-*cy*Hx** provided not only full conversion of **13**, but also a high selectivity to the hydrosilylation product **14**.

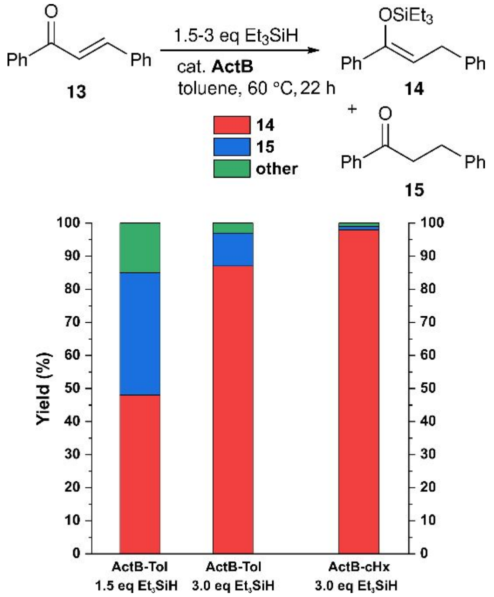

Scheme S2. Hydrosilylation of *trans*-chalcone (**13**) catalysed by **ActB** materials.

Benzil (**16**), a 1,2-diketone moiety, underwent both hydrosilylation and deoxygenation reactivity upon **ActB** catalysis (Scheme S3). Although the starting material was practically fully converted using the **ActB-Tol** catalyst, the reaction yielded a mixture of compounds, including only traces of the double hydrosilylation product **17**, but a substantial amount of the partially deoxygenated **18**, together with ether **19**, *trans*-stilbene **20**, and traces of the corresponding saturated diphenylethane **21**. Higher temperatures and an excess of Et_3SiH was necessary to shift the product distribution, at least in part, towards the fully deoxygenated products. On the other hand, **ActB-*cy*Hx** produced mainly a mixture of double silylated **17** and mono(silylether) **18**.

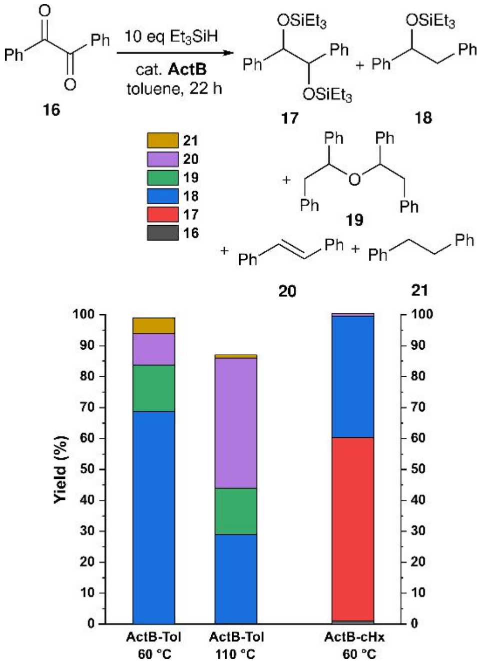

Scheme S3. Hydrosilylation/deoxygenation of benzil (**16**) catalysed by **ActB** materials.

Cyclohexanone (**22**) was chosen as an aliphatic cyclic ketone substrate for the hydrosilylation/deoxygenation reaction (Scheme S4). **ActB-Tol** afforded a mixture of products with the prevailing ether **24** accompanied with the simple hydrosilylation product **23** and deoxygenation products **25** and **26**. At higher temperature, again, the deoxygenation reactivity prevailed. The use of **ActB-*cy*Hx**, on the other hand, led to the formation of silyl ether **23** as the major product, which is consistent with the catalyst's general behaviour.

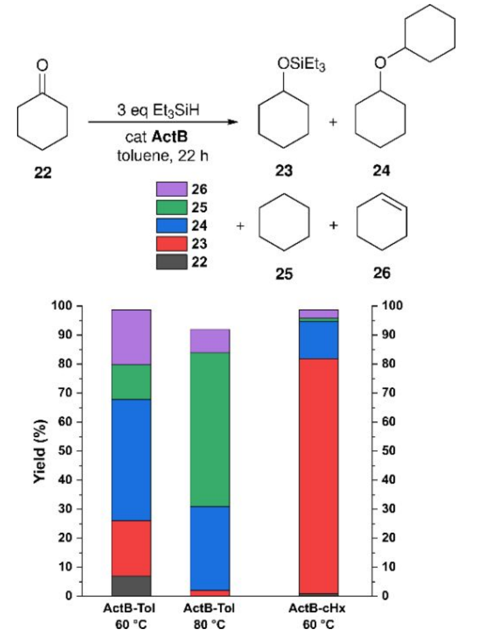

Scheme S4. Hydrosilylation/deoxygenation of cyclohexanone (**22**) catalysed by **ActB** materials.

As an example of an acyclic aliphatic ketone, we also employed 2-heptanone (**27** in Scheme S5). This reaction again demonstrated the tendency for deoxygenation of the **ActB-Tol** catalyst at higher temperatures, 100 °C was necessary for a full conversion of **27** in 22 h, while **ActB-*cy*Hx** gave the hydrosilylation product **28** almost exclusively (95% yield).

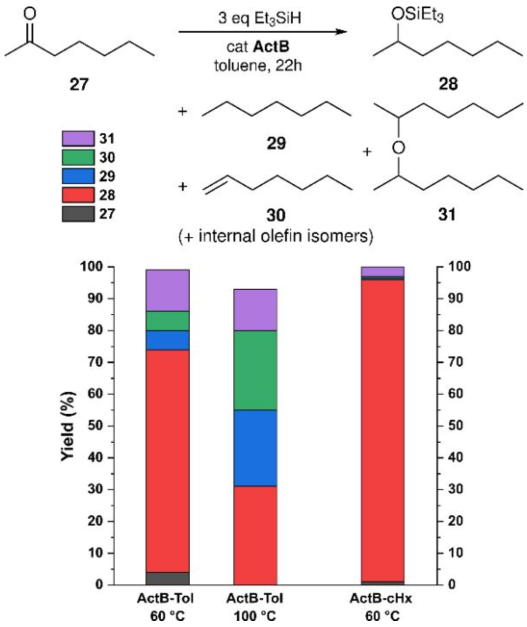

Scheme S5. Hydrosilylation/deoxygenation of 2-heptanone (**27**) catalysed by **ActB** materials.

Ethanol dehydration (gas flow reactor)

The **ActB** catalysts (50 mg, sieved in the 0.2–0.4 mm particle size range) were diluted with glass beads (0.5–1 mm) to keep the volume of the catalyst bed constant and placed in the catalytic reactor. The void space of the reactor was filled with glass beads to achieve a homogeneous distribution of the reaction mixture. The tests were carried out at atmospheric pressure, WHSV=2.2 h^{-1} for light-off experiments and 4.4 h^{-1} for overnight stability tests. Catalytic testing was carried out by injecting absolute ethanol using a NE-300 syringe pump. Ethanol with 5 mol% of pentane (internal standard) was fed in a 25 cm^3 min^{-1} flow of N_2 (3.4 mol% of ethanol in the mixture of N_2, ethanol, and pentane). The temperature was changed stepwise (170, 190, 210 and 240 °C). The heating ramp was set to ~4 °C min^{-1}. Stabilization at the set temperature lasted for 20 min. Analysis of effluent gas was carried out by HP 6890 Gas Chromatograph (6 injections at each temperature within 72 min) equipped with a flame ionization detector (FID) and a TG-BOND U column (30 m long, internal diameter 0.32 mm, film thickness 10 µm).

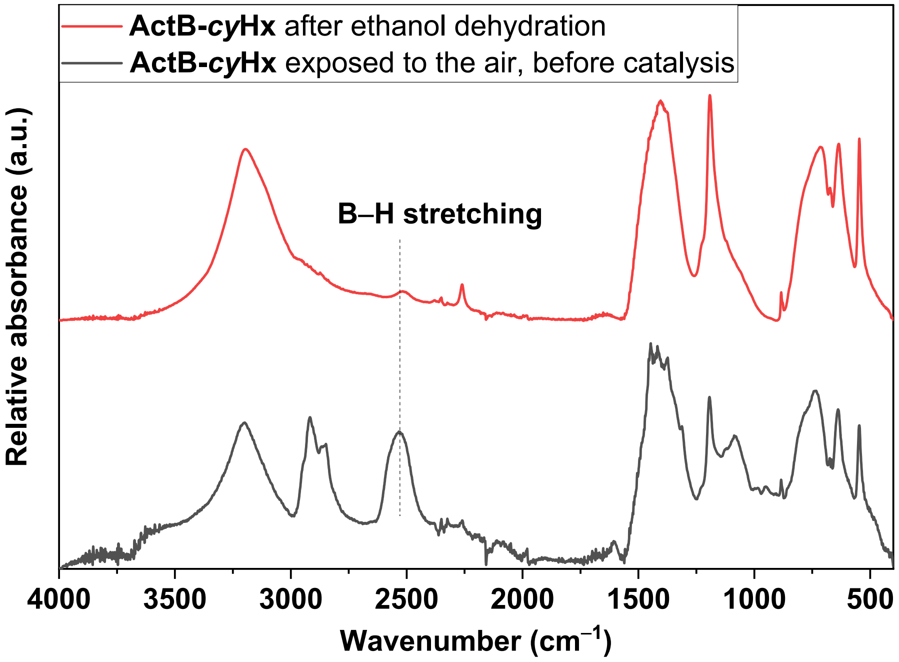

Figure S33. IR spectra of **ActB-*cy*Hx** before (black, down), and after (red, top) ethanol dehydration catalysis.

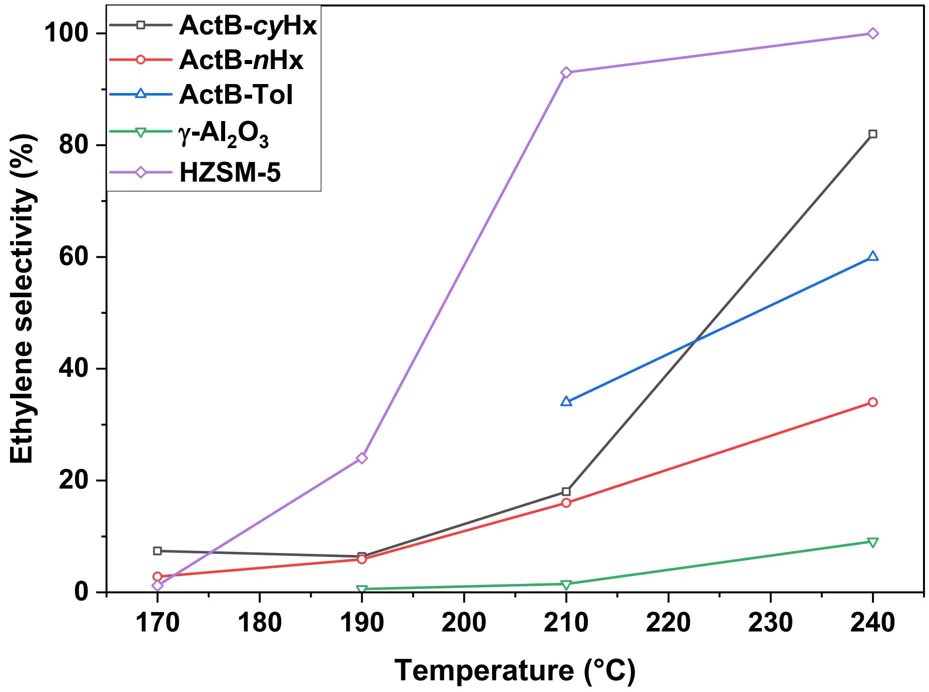

Figure S34. Ethylene selectivity in ethanol dehydration reaction at 170, 190, 210 and 240 °C.

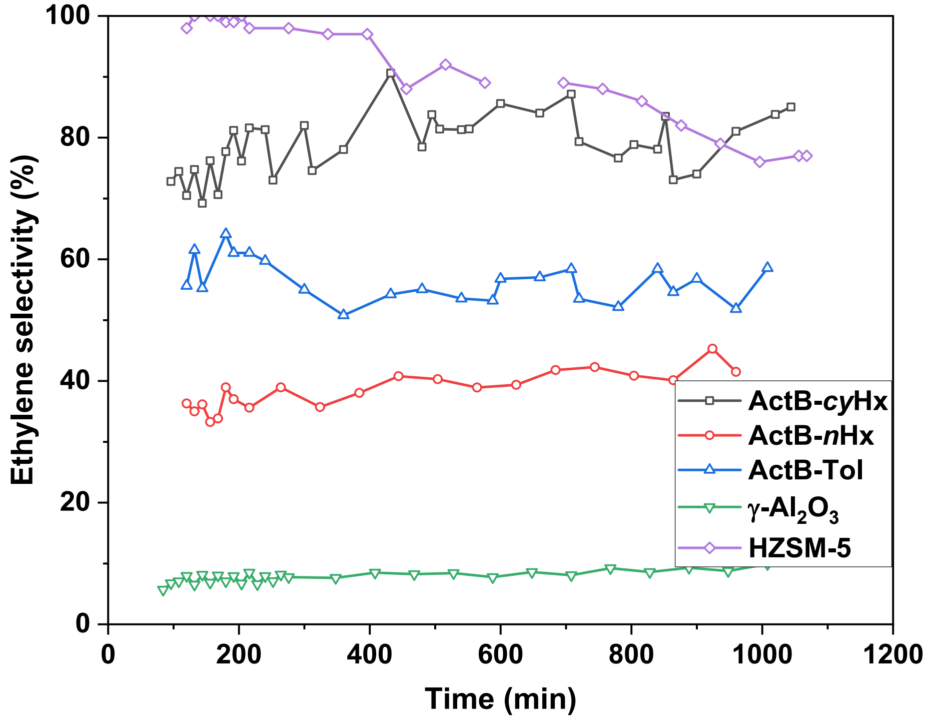

Figure S35. Ethylene selectivity at 240 °C overnight (stability test).

Table S22. Ethanol conversion (C), selectivity to ethylene ($S_{ethylene}$), yield of ethylene ($Y_{ethylene}$), diethyl ether (Y_{DEE}) and ethane (Y_{ethane}) and carbon balance (CB) in ethanol dehydration reaction at 170, 190, 210, and 240 °C. Weight hour space velocity (WHSV) was kept for all measurements 2.2 g g^{-1} h^{-1}.

Temperature (°C)	Sample	C (%)	$S_{ethylene}$ (%)	$Y_{ethylene}$ (%)	Y_{DEE} (%)	Y_{ethane} (%)	CB (%)
170	**ActB-*cy*Hx**	2.9	7.4	0.2	4.8	0.2	102
	**ActB-*n*Hx**	3.6	2.8	0.1	1.8	0.1	99
	**ActB-Tol**	~0	-	-	-	-	108
	γ-Al_2O_3	~0	-	-	-	-	109
	HZSM-5	44	1.2	0.5	53	-	110
190	**ActB-*cy*Hx**	22	6.4	1.4	12	0.5	92
	**ActB-*n*Hx**	6.7	5.9	0.4	3.4	0.4	98
	**ActB-Tol**	4.7	46	2.2	8.7	0.1	106
	γ-Al_2O_3	11	0.6	0.1	7.4	-	96
	HZSM-5	80	24	19	54	-	98
210	**ActB-*cy*Hx**	71	18	13	53	2	97
	**ActB-*n*Hx**	13	16	2.1	8.9	0.4	98
	**ActB-Tol**	25	34	8.6	24	0.5	107
	γ-Al_2O_3	20	1.5	0.3	18	-	99
	HZSM-5	97	93	92	4.0	-	94
240	**ActB-*cy*Hx**	95	82	78	13	3.6	100
	**ActB-*n*Hx**	49	34	17	32	0.2	99
	**ActB-Tol**	57	60	34	31	1.1	111
	γ-Al_2O_3	43	9.1	3.9	40	-	101
	HZSM-5	100	100	100	-	-	104

Table S23. Ethanol conversion (C) and selectivity to ethylene ($S_{ethylene}$) at 240 °C overnight (stability test). WHSV equals 4.4 g g^{-1} h^{-1}.

Time	ActB-*cy*Hx		ActB-*n*Hx		ActB-Tol		γ-Al_2O_3		HZSM-5*	
	C (%)	$S_{ethylene}$ (%)	C (%)	$S_{ethylene}$ (%)	C (%)	$S_{ethylene}$ (%)	C (%)	$S_{ethylene}$ (%)	C (%)	$S_{ethylene}$ (%)
120	92	70	44	36	51	56	36	8.0	98	98
132	93	75	43	35	55	62	33	6.5	99	100
144	92	70	43	36	51	55	36	8.1	98	101
156	93	76	44	33	51	55	33	6.8	97	100
168	92	71	42	34	40	66	36	8.0	99	100
180	93	78	45	39	45	64	34	7.1	95	99
192	93	81	42	37	47	61	36	7.9	94	99
216	93	82	44	36	48	61	37	8.5	93	98
264	92	73	48	39	49	60	36	8.2	93	98
324	92	75	46	36	49	55	29	7.6	92	97
384	93	78	51	38	46	51	34	8.5	90	97
444	94	90	56	41	50	54	33	8.2	88	88
504	93	77	56	40	52	55	35	8.4	84	92
564	87	81	54	39	50	54	33	7.8	82	90
624	94	86	54	39	52	57	34	8.6	83	89
684	94	84	60	42	55	57	34	8.1	83	89
744	93	79	61	42	52	53	36	9.2	83	88
804	93	79	57	41	50	52	31	8.6	82	86
864	94	73	57	40	51	55	35	9.3	79	82
924	93	74	61	45	55	57	33	8.8	76	82
960	93	81	60	42	50	52	35	9.9	76	79

*WHSV = 17.6 g g^{-1} h^{-1}

References

( ) Amoureux, J.P.; Fernandez, C.; Z Filtering in MQMAS NMR. Steuernagel S. *J. Magn. Reson.* **1996**, *123*, 116-118.
(2) Equbal, A.; Bjerring, M.; Madhu, P. K.; Nielsen, N. C. Improving spectral resolution in biological solid-state NMR using phase-alternated rCW heteronuclear decoupling. *Chem. Phys. Lett.* **2015**, *635*, 339-344.
(3) Brus, J. Heating of samples induced by fast magic-angle spinning. *Solid State Nucl. Magn. Reson.* **2000**, *16*, 151-160.
